# EIF3B stabilizes MAP2K2 to activate the ERK pathway and promote the progression of laryngeal squamous cell carcinoma

**DOI:** 10.1038/s41420-025-02634-2

**Published:** 2025-07-21

**Authors:** Jie Tan, Xueshi Li, Yuguang Wang, Lin Wang, Xingguo Zhao, Yixu Wang, Meng Cui

**Affiliations:** 1 Department of Otorhinolaryngology Head and Neck Surgery, Peking University People’s Hospital, Peking University, Beijing, PR China; 2 Department of Head and Neck Thyroid, Affiliated Cancer Hospital of Zhengzhou University & Henan Cancer Hospital, Zhengzhou, PR China

**Keywords:** Head and neck cancer, Cell biology, Ubiquitylation

## Abstract

To elucidate the role of eukaryotic translation initiation factor 3 subunit B (EIF3B) in laryngeal squamous cell carcinoma (LSCC) progression and its regulatory mechanism. Integrated bioinformatics analysis (GEO, TCGA), immunohistochemistry (IHC), lentiviral-mediated gene knockdown/overexpression, co-immunoprecipitation (Co-IP), Western blotting (WB), and in vivo xenograft models were employed. Clinically, our findings revealed an upregulation of EIF3B expression in LSCC, with its abnormally high levels significantly correlating with poor survival outcomes among patients. Functionally, ablation of EIF3B potently inhibited cancer cell proliferation, colony formation, and migratory abilities. Mechanistically, EIF3B stabilized MAP2K2 via direct interaction with its P3 domain, inhibiting VHL-mediated ubiquitination at K169. Notably, MAP2K2 kinase activity was essential for EIF3B-driven ERK phosphorylation and downstream oncogenic signaling. Moreover, EIF3B overexpression accelerated tumor growth in xenograft models, which was rescued by MAP2K2 knockdown. In Conclusion, EIF3B promotes LSCC progression by stabilizing MAP2K2, activating the ERK/MAPK pathway, and disrupting VHL-mediated proteostasis. Targeting the EIF3B-MAP2K2 axis may offer therapeutic strategies for LSCC.

## Introduction

Laryngeal squamous cell carcinoma (LSCC) is one of the most common malignant tumors of the head and neck, and more than 80,000 patients die from the disease every year [1]. LSCC has the characteristics of high invasion and easy metastasis, and is not easy to be controlled [2]. In addition, the prognosis of LSCC is poor, with a 5-year survival rate of less than 50% [3, 4]. At present, the main existing treatments are surgery, radiotherapy and chemotherapy [5]. Partial laryngectomy can retain the laryngeal function of patients with early LSCC, but the quality of pronunciation will be affected. Radiotherapy and chemotherapy can be used in some early LSCC, but most patients will have impaired function of pharynx mucosa and glands, dry mouth and dysphagia, resulting in a significant decline in life quality [6]. At the same time, radiotherapy and chemotherapy can increase the risk of thyroid cancer and other head and neck cancers [7]. Due to the lack of effective biomarkers for early diagnosis, most LSCC are diagnosed as advanced [8]. For advanced or recurrent LSCC, the current laryngeal function retention rate is extremely low. Therefore, it is urgent to explore the molecular mechanism of LSCC and find promising therapeutic targets to improve the clinical prognosis of LSCC patients.

As the initial step of protein synthesis, there are a variety of translation initiation cytokines involved in the process of translation [9]. Eukaryotic translation initiation cytokines (EIFs) play a central role in protein synthesis [10]. As a core member of the EIF family, EIF3 is composed of different protein subunits and almost participates in the whole process of translation initiation [11]. Eukaryotic translation initiation factor 3 subunit B (EIF3B, Gene ID: 8662) is considered to be a key scaffold protein in the EIF3 complex and exerts a crucial role in translation regulation and cell growth [12]. Previous studies have shown that ectopic expression of EIF3B can lead to malignant transformation of immortalized fibroblasts [13]. Moreover, EIF3B regulates the proliferation and apoptosis of chronic myeloid leukemia cell lines by regulating the expression of C3G [14]. Accumulated evidence indicated that EIF3B is overexpressed in various tumors and is significantly related to poor prognosis as well as tumor development. For example, high EIF3B expression was indicated to be an independent survival biomarker for patients with liver cancer [15]. Wang et al. demonstrated that EIF3B is associated with poor outcomes in gastric cancer patients and promotes cancer progression via the PI3K/AKT/mTOR signaling pathway [16]. Furthermore, Liu et al. reported that EIF3B may be a potential biomarker to improve the monitoring and treatment of non-small cell lung cancer [17].

In this study, the expression level of EIF3B in LSCC was revealed by Gene Expression Omnibus (GEO), Cancer Genome Atlas (TCGA) database and immunohistochemical (IHC). In addition, the role of EIF3B in LSCC was determined by functional loss/gain assays in vitro and in vivo. Collectively, the clinical significance, function and possible mechanism of EIF3B in LSCC were investigated. The study highlighted the significance of EIF3B in tumor progression and implicated EIF3B as a promising candidate target for LSCC treatment.

## Results

### The high expression of EIF3B in LSCC is correlated with survival

**Fig. 1:**
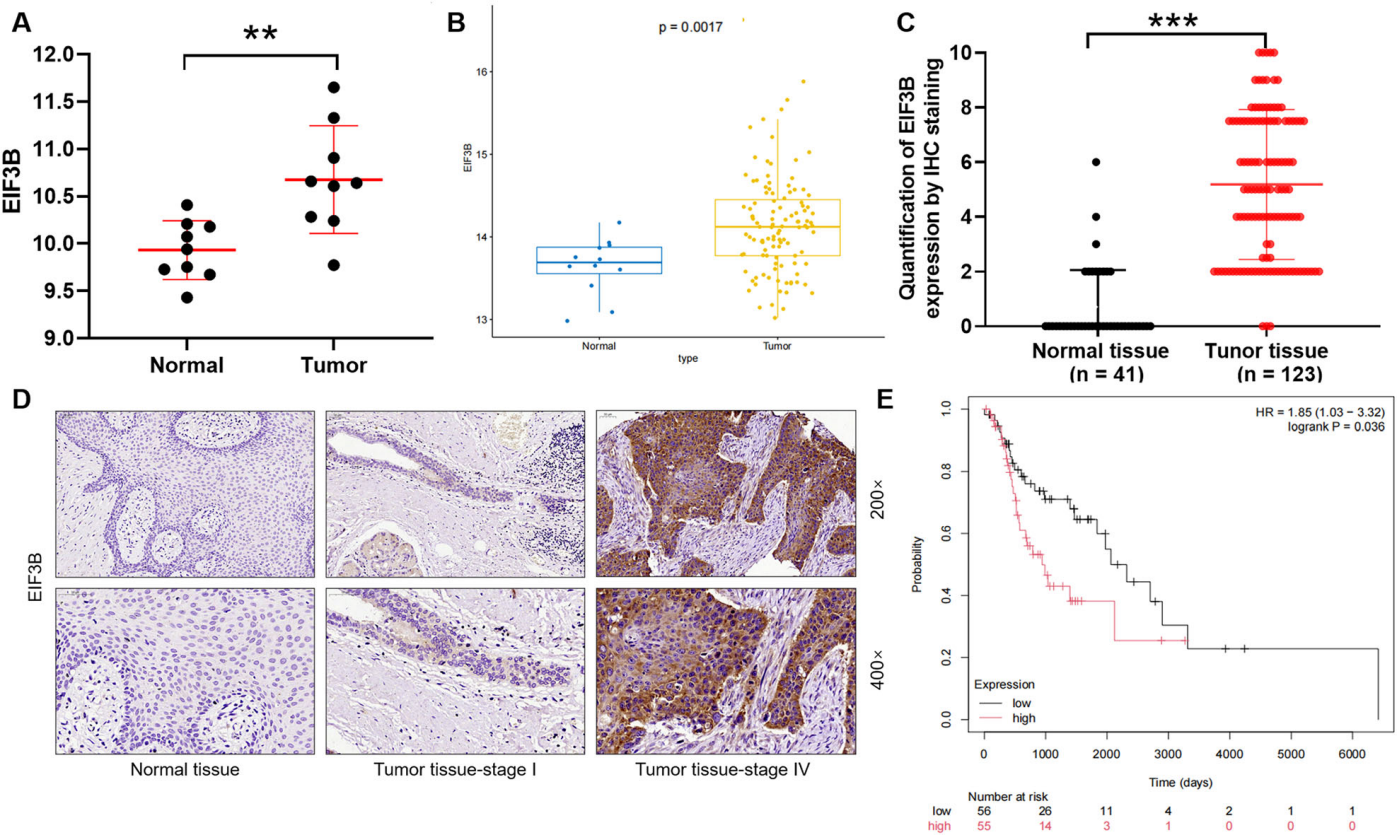
Correlation between EIF3B expression and LSCC. **A** Expression of EIF3B in LSCC tissue samples (*n* = 9) and paired non-tumor tissue samples (*n* = 9) was analyzed based on GEO database. **B** EIF3B expression was analyzed based on the Cancer Genome Atlas (TCGA) database of 111 tumor and 12 normal samples for LSCC. **C** Quantification of tissue IHC staining results to distinguish high or low expression levels of EIF3B. **D** IHC staining experiments on tumor tissues and paired normal tissues of clinical LSCC patients were conducted to clarify the expression intensity of EIF3B. **E** The correlation between EIF3B expression and survival of LSCC patients was performed based on the TCGA database.

From Gene Expression Omnibus (GEO) database, we identified that EIF3B was upregulated in LSCC tissue samples (*n* = 9) compared with paired non-tumor tissue samples (*n* = 9) (Fig. 1A). Moreover, we performed expression profiling and survival analysis based on the Cancer Genome Atlas (TCGA) database of 111 tumor and 12 normal samples for LSCC. The results indicated that the expression level of EIF3B was extremely abundant in LSCC (Fig. 1B). Meanwhile, we conducted IHC staining experiments on tumor tissues (n = 123) and paired normal tissues (*n* = 41) of clinical LSCC patients to clarify the expression intensity of EIF3B. According to the total score results, higher than 5 was defined as high expression of EIF3B, otherwise it was low expression. The high expression of EIF3B accounted for 45.5% of tumor tissues (*n* = 123), and only 2.4% of normal tissues (*n* = 41) (Fig. 1C). As illustrated in Fig. 1D, the signal intensity of EIF3B in LSCC tissues was stronger than that in normal tissues, which confirmed the above analysis results. In addition, we performed survival analysis based on TCGA database of 111 tumor and 12 normal samples for LSCC. The results indicated that the expression level of EIF3B was negatively correlated with the survival of this patient (Fig. 1E). Furthermore, the relationship between EIF3B expression and tumor characteristics of clinical LSCC patients (*n* = 123) was analyzed. Our data indicated that the expression level of EIF3B was positively correlated with age, T Infiltrate, lymphatic metastasis, tumor size, and stage (Table 1). Consistently, the results of Spearman Correlation suggested that its aberrantly high expression associated with poor prognostic factor of LSCC patients (Table 2). Collectively, EIF3B expression was significantly correlated with tumor pathological features, which may serve as a diagnostic marker.

**Table 1:** Relationship between EIF3B expression and tumor characteristics in patients with LSCC.

Features	No. of patients	EIF3B expression		*p* value
		low	high	
All patients	123	67	56	
Age (years)				0.027
<62	59	26	33	
≥62	64	41	23	
Gender				0.509
Male	118	65	53	
Female	5	2	3	
Differentiation				0.986
Low	11	5	6	
Medium	37	22	15	
High	73	40	33	
T Infiltrate				0.014
T1	38	27	11	
T2	51	25	26	
T3	21	11	10	
T4	13	4	9	
lymphatic metastasis (N)				0.007
N0	79	50	29	
N1	13	6	7	
N2	31	11	20	
Tumor size				0.010
≤ 2cm	64	42	22	
> 2cm	59	25	34	
Stage				0.003
I	29	23	6	
II	29	14	15	
III	26	15	11	
IV	39	15	24

**Table 2:** Relationship between EIF3B expression and tumor characteristics in patients with LSCC.

			EIF3B
Age (years)		Spearman correlation	−0.201
		Significance (double-tailed)	0.026
		*N*	123
Tumor size		Spearman correlation	0.233
		Significance (double-tailed)	0.009
		*N*	123
T Infiltrate		Spearman correlation	0.224
		Significance (double-tailed)	0.013
		*N*	123
lymphatic metastasis (*N*)		Spearman correlation	0.244
		Significance (double-tailed)	0.006
		*N*	123
Stage		Spearman correlation	0.267
		Significance (double-tailed)	0.003
	*N*		123

### EIF3B is essential for the proliferation, migration and tumor formation of LSCC

**Fig. 2:**
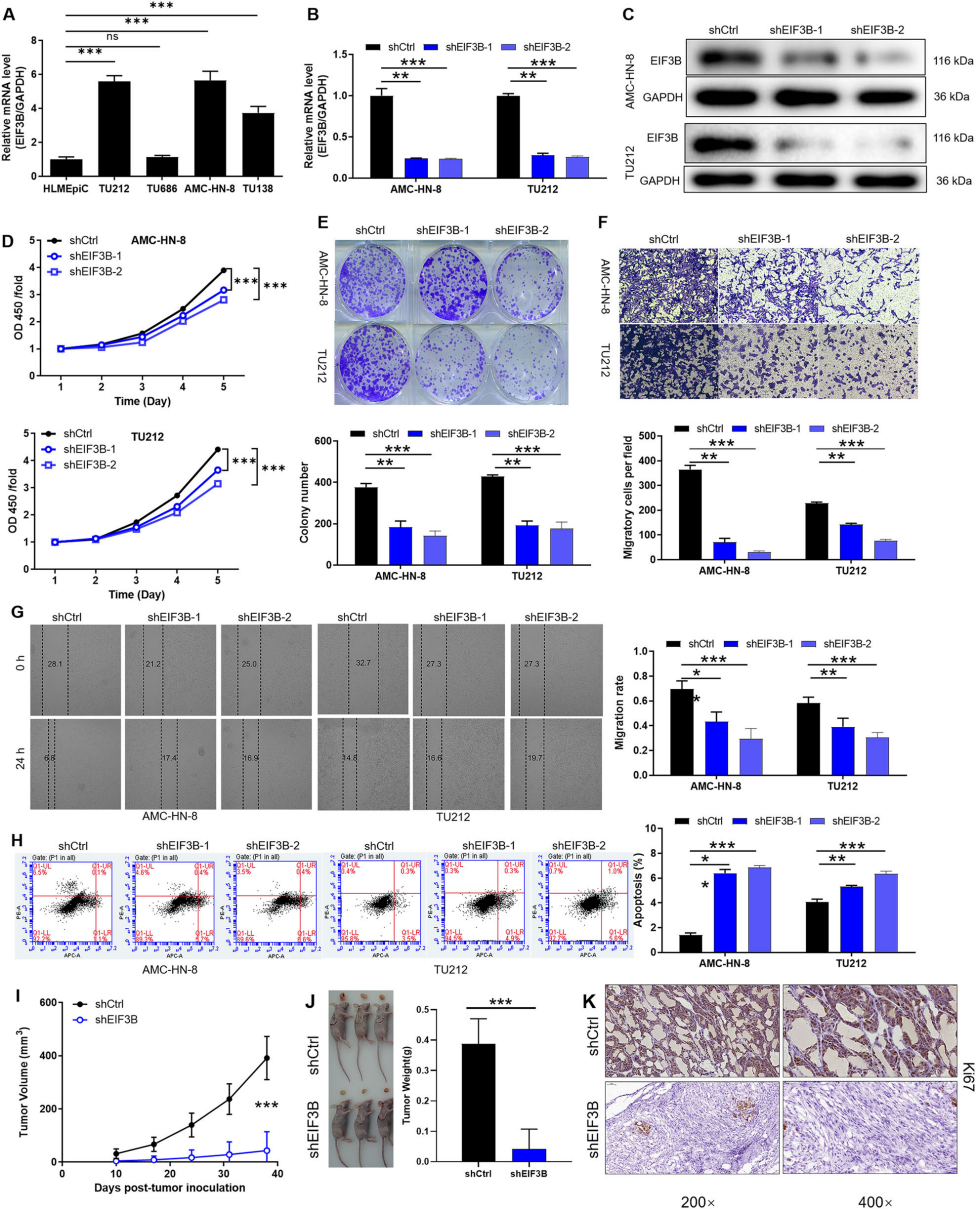
Knockdown of EIF3B inhibits malignant biological behavior of LSCC cells. **A** The relative mRNA expression of EIF3B in human nasopharyngeal epithelial cells NP-69 and LSCC cell lines AMC-HN-8, TU212, TU686 was evaluated using qPCR. The specificity and validity of the lentivirus-mediated shRNA knockdown of EIF3B expression were verified by qPCR (**B**) and WB analysis (**C**). **D** Cell proliferation of AMC-HN-8 and Tu212 cells with or without knockdown of EIF3B was evaluated by MTT assay. **E** The ability of cell clone formation was observed in AMC-HN-8 and Tu212 cells with or without knockdown of EIF3B. Cell migration of AMC-HN-8 and Tu212 cells with or without knockdown of EIF3B was evaluated by Transwell assay (**F**) and wound-healing assay (**G**). **H** Flow cytometry analysis based on Annexin V-APC staining was utilized to detect cell apoptotic ratio in AMC-HN-8 and Tu212 cells. After injecting AMC-HN-8 cells (shCtrl, shEIF3B) into the mouse xenograft model, the tumor volume (**I**) and tumor weight (**J**) were detected respectively. **K** The tumor tissues of each group of mice were performed IHC staining to identify the Ki67 expression. The representative images were selected from at least 3 independent experiments. The data were presented as the mean ± SD (*n* = 3). **P < 0.01, ***P < 0.001.

The relative mRNA expression levels of EIF3B were quantitatively assessed in human nasopharyngeal epithelial cells (NP-69) and laryngeal squamous cell carcinoma (LSCC) cells (AMC-HN-8 and TU212) using qPCR. Our findings revealed that EIF3B expression was significantly upregulated in AMC-HN-8 and TU212 cells compared to NP-69 cells (Fig. 2A). Subsequently, a lentiviral vector expressing shRNA specifically targeting EIF3B (shEIF3B-1 and shEIF3B-2) was constructed to knock down EIF3B expression in AMC-HN-8 and TU212 cells (Fig. S1A). Successful transfection was confirmed by observing GFP expression levels exceeding 80% in both cell lines, indicating efficient lentiviral delivery (Fig. S1B). As expected, qPCR analysis demonstrated a substantial reduction in EIF3B mRNA levels in AMC-HN-8 and TU212 cells with shEIF3B-1 and shEIF3B-2, when compared to control cells transduced with a non-targeting shRNA (shCtrl) (Fig. 2B). This downregulation was further corroborated at the protein level, with the EIF3B protein abundance markedly decreased in the shEIF3B-treated groups (Fig. 2C).

To validate the functional consequences of EIF3B knockdown, in vitro phenotypic assays were performed on AMC-HN-8 and TU212 cells. Notably, cell proliferation was effectively suppressed in both cell lines following EIF3B knockdown (Fig. 2D). Furthermore, the shEIF3B-treated cells exhibited smaller and fewer colony-forming units compared to their respective controls (Fig. 2E), indicating impaired clonogenic potential. Additionally, the migration ability of EIF3B down-regulated LSCC cells AMC-HN-8 and TU212 decreased sharply within 24 h after starvation treatment (Fig. 2F, G), underscoring the critical role of EIF3B in facilitating the aggressive behavior of these cancer cells. Consistent with these findings, cell viability was significantly decreased, while apoptosis was induced in EIF3B-knockdown cells (Fig. 2H).

Moreover, knockdown EIF3B and control AMC-HN-8 cells were injected subcutaneously into nude mice to analyze the effects on tumor formation. After a period of monitoring, SHEIF3B-treated cells showed smaller tumor bodies compared to controls (Fig. 2I, J), indicating impaired tumorigenic potential. Consistent with these findings, tumor tissue with EIF3B knockdown showed weaker Ki67 expression (Fig. 2K), suggesting impaired growth. Taken together, the in vitro and in vivo data highlight the critical role of EIF3B in promoting the proliferation, migration, and tumorigenic behavior of LSCC cells.

### EIF3B stabilizes the expression of MAP2K2 by attenuating VHL-mediated ubiquitination of MAP2K2

RNA sequencing was employed to comprehensively identify differentially expressed genes (DEGs) in AMC-HN-8 cells between the shCtrl and shEIF3B groups. Applying stringent criteria of |Fold Change | ≥ 2.0 and False Discovery Rate (FDR) < 0.05, hierarchical clustering analysis revealed distinct transcriptional profiles between the two groups, highlighting significant differences. Specifically, we identified 2533 genes as upregulated and 1802 genes as downregulated in AMC-HN-8 cells upon EIF3B knockdown (shCtrl vs shEIF3B, *n* = 3), as depicted in Fig. S2A. To validate the RNA-seq findings, we selected a subset of top-ranked DEGs, including MAP2K2, for further confirmation using qPCR and WB in AMC-HN-8 cells. Our results consistently demonstrated that MAP2K2 exhibited the most pronounced downregulation among the analyzed DEGs, as shown in Fig. S2B and Fig. S2C, reinforcing its key role in the transcriptional and translational consequences of EIF3B knockdown.

**Fig. 3:**
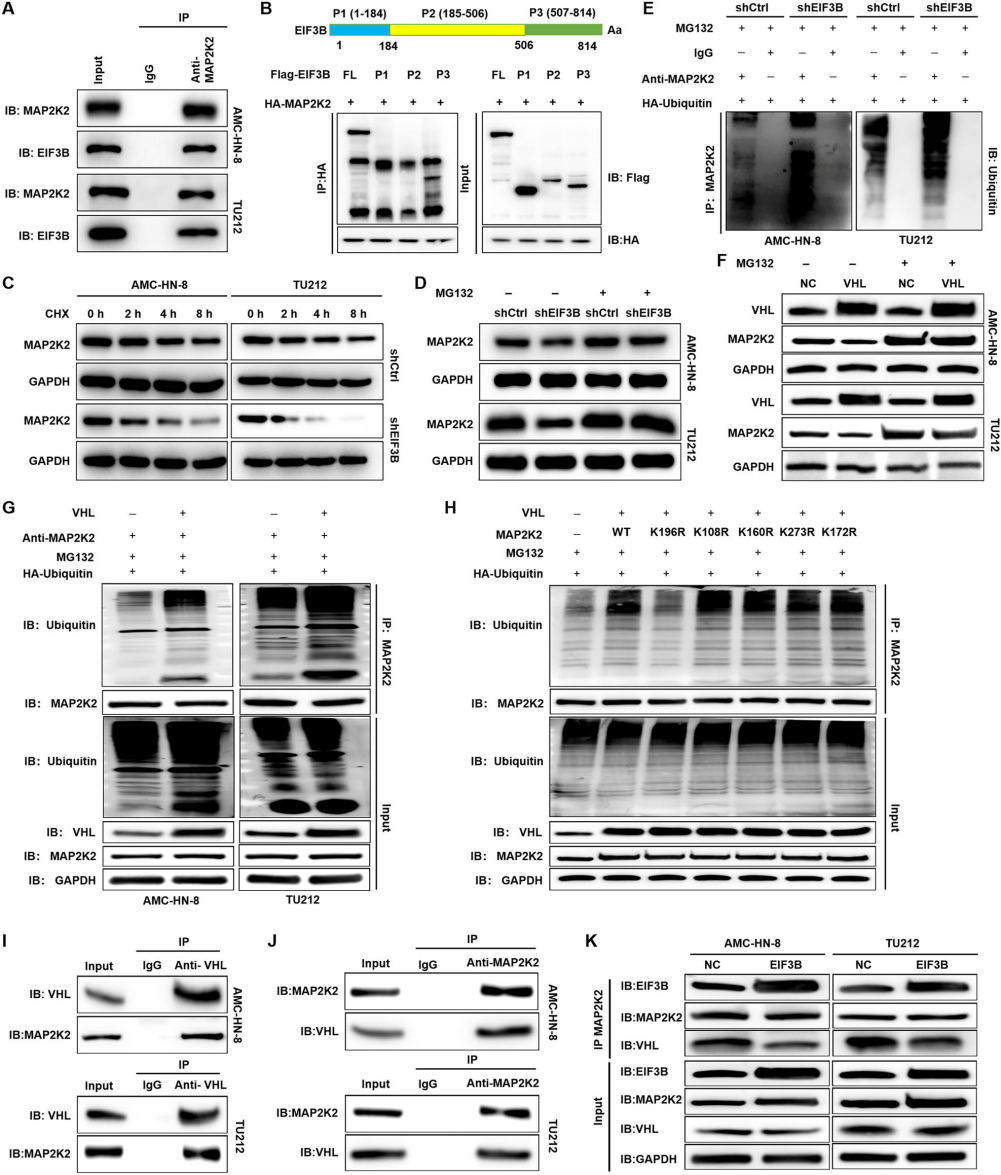
EIF3B stabilizes the expression of MAP2K2 by attenuating VHL-mediated ubiquitination of MAP2K2. **A** IgG antibody and anti-MAP2K2 antibody were used for Co-IP to determine the interaction between EIF3B and MAP2K2. **B** Plasmids containing FL (full length), P1 (1-184), P2 (185-506), and P3 (507-814) domain of EIF3B were co-expressed with HA-MAP2K2 in HEK293 T cells. Lysates were immunoprecipitated with S-beads. **C** AMC-HN-8 and Tu212 cells with or without EIF3B knockdown were treated with CHX (0.2 mg/mL), and the stability of MAP2K2 protein was examined. **D** AMC-HN-8 and Tu212 cells with or without EIF3B knockdown were treated with proteasome inhibitor MG-132 10 μM for 8 h, and the stability of MAP2K2 protein was examined. **E** After EIF3B knockdown and control AMC-HN-8 and Tu212 cells were treated with 10 μM MG132, the ubiquitination of MAP2K2 was detected by specific antibodies. **F** VHL overexpression and control AMC-HN-8 and Tu212 cells were treated with MG-132, and the stability of MAP2K2 protein was examined. **G** After VHL overexpression and control AMC-HN-8 and Tu212 cells were treated with 10 μM MG132, the ubiquitination of MAP2K2 was detected by specific antibodies. **H** Ubiquitination assay of MAP2K2 in VHL overexpression and control HEK293T cells with HA-Ubiquitin, Flag-WT, Flag-MAP2K2-K108R/K160R/K172R/K196R/K273R and treated with 10 μM MG132 for 6 h. **I**, **J** The AMC-HN-8 and Tu212 cell lysates were immunoprecipitated with VHL-antibody and anti-MAP2K2 antibody, and western blotting was performed with anti-MAP2K2 antibody and VHL antibody to confirm the specific interaction between VHL and MAP2K2. **K** WB detection was performed using VHL-antibody and anti-MAP2K2 antibody in EIF3B overexpression and control AMC-HN-8 and Tu212 cells to evaluate the interaction between VHL and MAP2K2.

Furthermore, our findings revealed the existence of endogenous EIF3B within MAP2K2 immunoprecipitants derived from AMC-HN-8 and TU212 cells, validating the specific interplay between MAP2K2 and EIF3B (Fig. 3A). Upon dissecting the structural composition of EIF3B, we identified three distinct domains: P1 (amino acids 1-184), P2 (amino acids 185-506), and (amino acids 507-814). Co-immunoprecipitation assays utilizing purified recombinant proteins revealed a tight interaction between MAP2K2 and the P3 domain of EIF3B, while no such interaction was observed with P1 or P2 (Fig. 3B).

To delve into the potential role of EIF3B in regulating MAP2K2 protein stability, we conducted cycloheximide (CHX) chase experiments. By inhibiting protein translation with CHX in AMC-HN-8 and Tu212 cells, we observed that the knockdown of EIF3B significantly accelerated the degradation of MAP2K2 protein (Fig. 3C). Notably, this destabilizing effect of EIF3B knockdown on MAP2K2 was partially mitigated when the cells were treated with the proteasome inhibitor MG-132 for 8 h (Fig. 3D), suggesting the involvement of the ubiquitin-proteasome system in this regulation. Subsequent investigations into the regulation of MAP2K2 ubiquitination by EIF3B in AMC-HN-8 and Tu212 cells confirmed that the downregulation of EIF3B markedly promoted the ubiquitination of MAP2K2 (Fig. 3E). Overall, the results not only underscore the specific interaction between the P3 domain of EIF3B and MAP2K2 but also demonstrate that the loss of EIF3B leads to ubiquitination-mediated degradation of MAP2K2.

Ubibrowser database predictions identified von Hippel-Lindau (VHL), an E3 ubiquitin ligase, as a potential regulator of MAP2K2 ubiquitination. Overexpression of VHL destabilized endogenous MAP2K2 protein levels in AMC-HN-8 and Tu212 cells following MG-132 treatment, as demonstrated by Western blot analysis (Fig. 3F). Consistently, VHL overexpression significantly increased MAP2K2 polyubiquitination in MG-132-treated cells (Fig. 3G). To map the ubiquitination sites on MAP2K2, we analyzed predicted lysine residues (K108, K160, K172, K196, K273) using GPS-Uber. Sequential mutagenesis of these residues (K108R, K160R, K172R, K196R, K273R) revealed that only the K169R mutant attenuated VHL-mediated MAP2K2 ubiquitination (Fig. 3H), identifying lysine 169 (K169) as the critical ubiquitination site regulated by VHL.

Using MAP2K2 or VHL antibodies for immunoprecipitation followed by Western blotting with reciprocal antibodies, we observed a direct interaction between VHL and MAP2K2 in AMC-HN-8 and Tu212 cells (Fig. 3I, J). Notably, EIF3B overexpression reduced VHL protein levels (Fig. 3K), suggesting that EIF3B disrupts the VHL-MAP2K2 interaction. Collectively, these findings demonstrate that EIF3B stabilizes MAP2K2 by impeding VHL-mediated ubiquitination at K169, thereby maintaining elevated MAP2K2 levels to promote oncogenic progression.

### EIF3B promotes the proliferation and migration of LSCC cells through MAP2K2

**Fig. 4:**
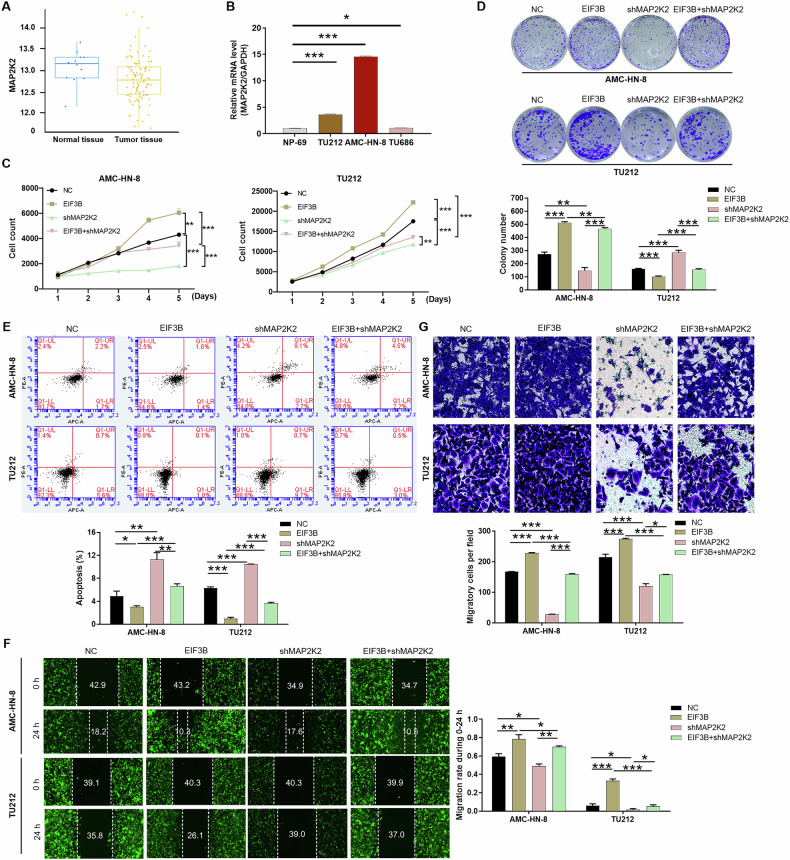
Knockdown of MAP2K2 alleviates the promotion effects of EIF3B overexpression in LSCC cells. **A** MAP2K2 expression was analyzed based on the Cancer Genome Atlas (TCGA) database of 111 tumor and 12 normal samples for LSCC. **B** The relative mRNA expression of MAP2K2 in human nasopharyngeal epithelial cells NP-69 and LSCC cell lines AMC-HN-8, TU212, TU686 was evaluated using qPCR. AMC-HN-8 and Tu212 cells transfected with control plasmids, MAP2K2 overexpression plasmids, NC, and simultaneous shEIF3B and MAP2K2 overexpression were subjected to the detection of cell proliferation (**C**), colony formation (**D**), cell apoptosis (**E**), cell migration by wound-healing assay (**F**) and Transwell assay (**G**). The representative images were selected from at least 3 independent experiments. The data was presented as the mean ± SD (*n* = 3). *P < 0.05, **P < 0.01, ***P < 0.001.

Utilizing the TCGA database, we discovered a remarkably high expression of MAP2K2 in LSCC tissues (Fig. 4A). Consistently, LSCC cell lines (AMC-HN-8 and TU212) exhibited higher relative mRNA levels of MAP2K2 compared to the nasopharyngeal epithelial cells (NP-69) (Fig. 4B). To elucidate the underlying molecular mechanism linking EIF3B and MAP2K2, we conducted a series of in vitro cellular functional assays. Subsequently, we engineered AMC-HN-8 and TU212 cells with MAP2K2-knockdown (shMAP2K2) and EIF3B-overexpression (EIF3B) constructs, following the previously described methodology (Fig. S3A–S3D). Our findings revealed that AMC-HN-8 and TU212 cells overexpressing EIF3B exhibited enhanced proliferation and migration capabilities, along with reduced apoptosis rates (Fig. 4C–G). Conversely, MAP2K2 knockdown significantly impeded the malignant progression of AMC-HN-8 cells, manifesting as decreased proliferation, increased apoptosis, and slowed migration (Fig. 4C–G). Notably, the promoting effects of EIF3B overexpression on AMC-HN-8 cells were partially mitigated by MAP2K2 knockdown (Fig. 4C–G). As a result, the data demonstrated that EIF3B promotes the proliferation and migration of LSCC cells through MAP2K2.

### EIF3B modulates ERK phosphorylation in LSCC cells via the kinase activity of MAP2K2

**Fig. 5:**
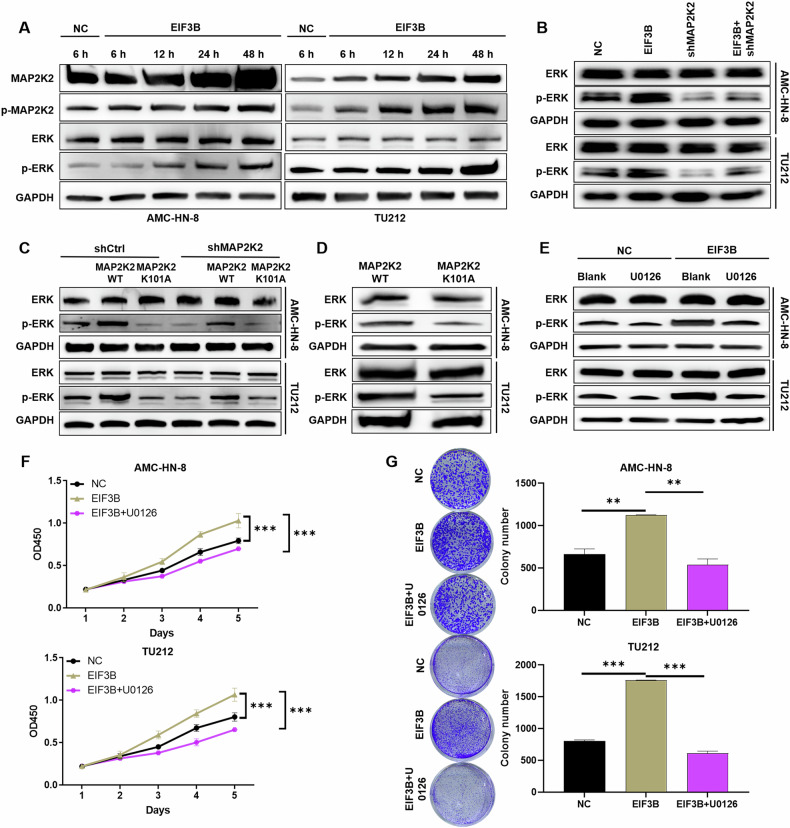
EIF3B modulates ERK phosphorylation in LSCC cells via the kinase activity of MAP2K2. **A** Cell lysates were collected at 6, 12, 24, and 48 h in AMC-HN-8 and TU212 cells that induced EIF3B overexpression and control. The total levels and phosphorylation levels of MAP2K2 and ERK were detected by WB. **B** The total levels and phosphorylation levels of ERK were detected by WB in AMC-HN-8 and Tu212 cells with EIF3B overexpression or MAP2K2 knockdown. **C** Wild-type MAP2K2 (WT) or kinase death mutant (K101A) was re-expressed in MAP2K2 knockdown and control AMC-HN-8 and TU212 cells. The total level and phosphorylation level of ERK were detected by WB. **D** Purify MAP2K2 (WT or K101A) and ERK proteins. In the presence of ATP, MAP2K2 and ERK were incubated, and total level and phosphorylation level of ERK were detected by WB. **E** The cells AMC-HN-8 and TU212 with EIF3B overexpression and control were pretreated with the MEK inhibitor U0126 (10 μM) for 1 h, and total level and phosphorylation level of ERK were detected. The proliferation (**F**) and clonal formation (**G**) of EIF3B overexpressed AMC-HN-8 and Tu212 cells were examined by MAPK/ERK signaling pathway inhibitor (U0126). The representative images were selected from at least 3 independent experiments. The data were presented as the mean ± SD (*n* = 3). **P < 0.01, ***P < 0.001.

Delving deeper into the downstream regulatory network of EIF3B, we uncovered MAP2K2 as a pivotal player, implicated in diverse signaling pathways and functions, notably the ERK/MAPK signaling cascade (Fig. S4A). Overexpression of EIF3B in AMC-HN-8 and TU212 cells significantly increased phosphorylation levels of both MAP2K2 and ERK in a time-dependent manner, peaking at 48 h post-transfection (Fig. 5A). Consistent with these findings, EIF3B knockdown markedly reduced ATP-dependent ERK phosphorylation in these cells (Fig. S4B). Notably, MAP2K2 depletion abrogated the EIF3B-induced ERK activation, confirming that MAP2K2 mediates EIF3B’s effects on ERK phosphorylation (Fig. 5B).

To determine whether MAP2K2 acts as a direct upstream kinase for ERK in this context, we re-expressed wild-type MAP2K2 (WT) or a kinase-dead mutant (K101A) in EIF3B-knockdown cells. In vitro kinase assays revealed that WT MAP2K2, but not the K101A mutant, restored ERK phosphorylation in EIF3B-depleted cells (Fig. 5C, D), demonstrating that MAP2K2’s catalytic activity is essential for ERK activation.

Pharmacological blockade of MEK with U0126 (10 μM) completely suppressed EIF3B-induced ERK phosphorylation in AMC-HN-8 and TU212 cells (Fig. 5E), confirming that EIF3B drives ERK activation through the canonical MEK-ERK axis. Functional validation using clonogenic and proliferation assays showed that U0126 treatment markedly attenuated the pro-proliferative effects of EIF3B overexpression (Fig. 5F, G). Collectively, these data establish a linear signaling axis where EIF3B enhances ERK/MAPK signaling via MAP2K2-mediated MEK activation, thereby driving tumor cell proliferation.

### EIF3B is dependent on MAP2K2 to drive LSCC tumor growth

**Fig. 6:**
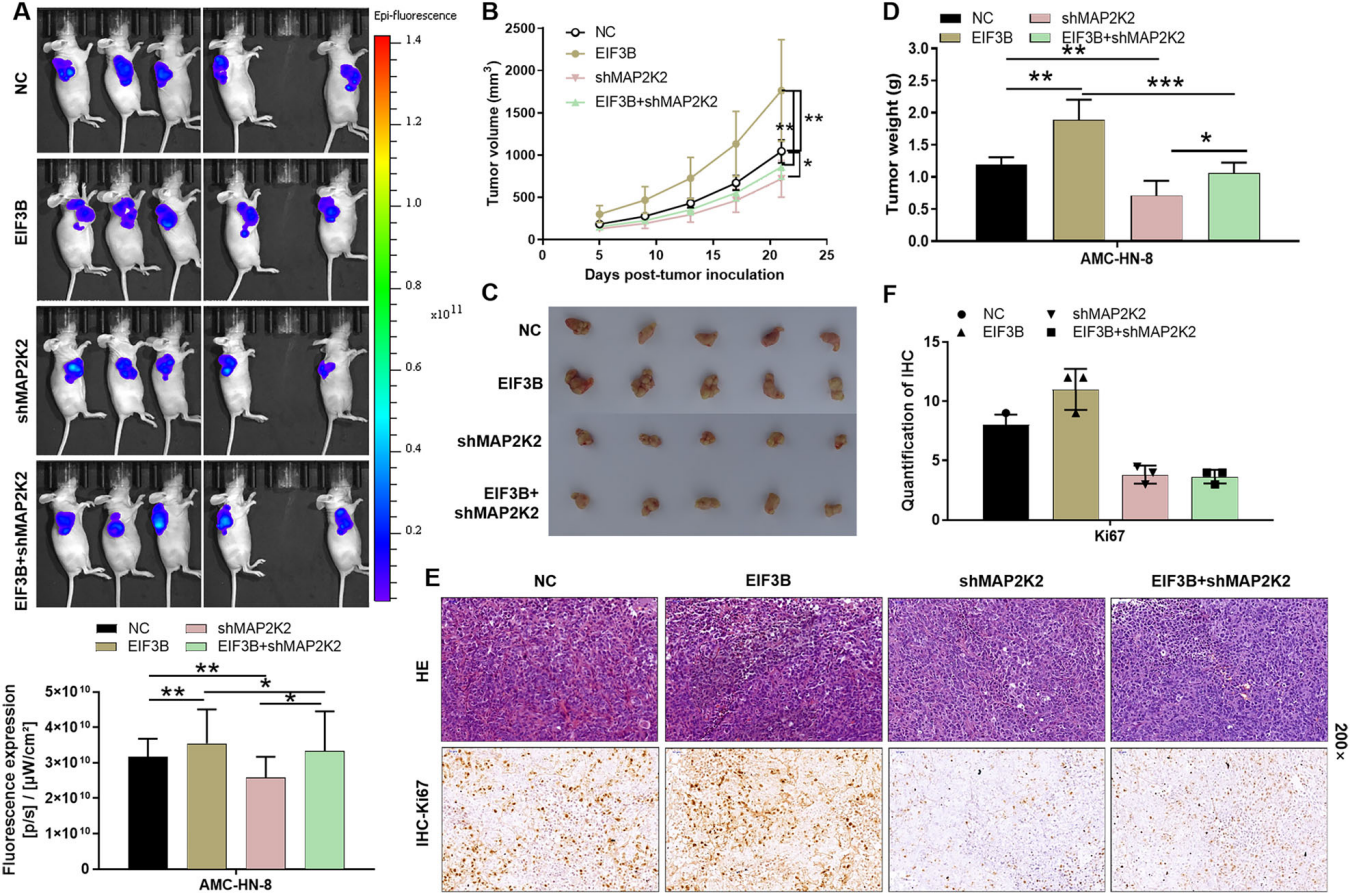
MAP2K2 knockdown attenuates the promotion of EIF3B overexpression to tumor growth. **A**–**D** After injecting AMC-HN-8 cells (NC, EIF3B, shMAP2K2, EIF3B+ shMAP2K2) into the mouse xenograft model, the fluorescence intensity (**A**), tumor volume (**B**, **C**), and tumor weight (**D**) were detected respectively. **E** The tumor tissues of each group of mice were stained with hematoxylin and eosin (H&E) and IHC staining to identify the Ki67 expression in tumors. **F** Quantification of Ki67 immunohistochemical staining in tumor sections from different groups. The data were presented as the mean ± SD (*n* = 5). *P < 0.05, **P < 0.01, ***P < 0.001.

To validate the in vivo roles of EIF3B and MAP2K2, we established subcutaneous xenograft tumor models in mice. As depicted in Fig. 6A, the total fluorescence intensity served as a surrogate marker for tumor burden, revealing that EIF3B overexpression intensified fluorescence intensity compared to the negative control (NC), whereas MAP2K2 knockdown diminished it. After 21 days of continuous observation, mice with EIF3B overexpression exhibited significantly smaller tumor volumes (Fig. 6B, C), corroborated by lighter tumor weights compared to the control group (Fig. 6D). Conversely, tumor size was markedly reduced in the MAP2K2-downregulated group (Fig. 6B–D).

To histologically confirm the tumor nodules, tumor tissues from each group were subjected to hematoxylin and eosin (H&E) staining. Furthermore, IHC staining for Ki67, a marker of cellular proliferation, was performed. Representative images revealed that Ki67 expression was enhanced in the EIF3B overexpression group, indicative of increased proliferative activity, whereas it was attenuated in the MAP2K2 knockdown group (Fig. 6E). Collectively, our findings suggest that MAP2K2 knockdown counteracts the tumor-promoting effects of EIF3B overexpression, reinforcing the importance of the EIF3B-MAP2K2 axis in regulating tumor growth.

## Discussion

Our study identifies EIF3B as a critical oncoprotein in LSCC, with its overexpression linked to aggressive tumor behavior and poor prognosis (Fig. 1). This aligns with emerging evidence of EIF3B’s oncogenic roles in other cancers, where EIF3B overexpression correlates with metastasis and reduced survival. Notably, EIF3B’s prognostic value in LSCC surpasses that of conventional markers like EGFR, which shows moderate correlation with tumor size but lacks specificity for aggressive subtypes [18].

The dependency of LSCC proliferation on the EIF3B-MAP2K2-ERK axis is underscored by the reduction in clonogenic survival (Fig. 5G) following U0126 treatment. This mirrors findings in HCC, where targeting the TGFBI/MAPK/ERK pathway (downstream of EIF3B) suppressed migration and invasion [18]. However, our study uniquely identifies EIF3B as an upstream regulator of MAP2K2 stability, distinct from its canonical role in translation. For instance, in gastric cancer, EIF3B activates the PI3K/AKT/mTOR pathway by enhancing mTORC1 signaling, indirectly modulating MAPK activity [16]. In contrast, our work identifies a direct, kinase-dependent interaction between EIF3B and MAP2K2. In addition, Xu et al. reported that EIF3B promotes CEBPB translation, activating MAPK signaling to drive invasion and metastasis [19]. Here, EIF3B facilitates CEBPB mRNA 5′UTR binding, enhancing its translation—a mechanism distinct from our observed stabilization of MAP2K2. In prostate cancer, CircPDE5A regulates EIF3C (a paralog of EIF3B) to suppress m6A methylation of EIF3C mRNA, indirectly influencing MAPK signaling [20]. This underscores EIF3 family members’ context-dependent roles in MAPK regulation. Additionally, Wu et al. reported that FOXA1-dependent PUS1 regulates EIF3b stability in a non-enzymatic pathway mediating prostate cancer bone metastasis [21]. Collectively, our study reveals a novel mechanism where EIF3B stabilizes MAP2K2 (Gene ID: 5605), a key upstream kinase in the ERK/MAPK pathway (Figs. 3–5). This interaction occurs via EIF3B’s P3 domain, disrupting VHL-mediated ubiquitination and proteasomal degradation of MAP2K2 (Fig. 3H–K). The dependence on MAP2K2’s kinase activity for ERK activation (Fig. 5C, D) underscores its catalytic role in propagating oncogenic signals. This contrasts with its classical role as a translation initiation factor but aligns with emerging evidence of EIF3B’s multifunctionality in cancer biology.

The interplay between EIF3B and the ubiquitin-proteasome system (via VHL) represents a paradigm shift in MAPK regulation. Unlike stress-induced MAPK activation (e.g., oxidative stress triggering JNK/p38) [22], which relies on transient ubiquitination, EIF3B-mediated MAP2K2 stabilization involves chronic suppression of VHL-mediated degradation. This mechanism is analogous to the role of MDM2 in stabilizing p53 under oncogenic stress [23], but here, EIF3B disrupts a tumor suppressor (VHL) to sustain MAP2K2 levels. Such a dual function—simultaneously acting as a translation factor and a proteostasis regulator—has not been reported for other EIF3 family members.

Nevertheless, there are still some limitations in this study. The subcutaneous xenograft model (Fig. 6A–E) provides preliminary evidence but lacks immune and stromal components critical for LSCC progression. Orthotopic models in immunocompetent mice or patient-derived xenografts (PDXs) would better recapitulate tumor heterogeneity and microenvironmental interactions. For instance, PDX models of HNSCC have demonstrated improved predictive accuracy for therapeutic responses [24]. On the other hand, exploring EIF3B inhibitors with existing MAPK pathway blockers (e.g., trametinib) could synergistically enhance efficacy, building on preclinical data from HCC where EIF3B knockdown synergized with mTOR inhibitors. Additionally, combining EIF3B inhibitors with immunotherapies (e.g., anti-PD-1) may restore T-cell activity in EIF3B-high tumors, which exhibit immunosuppressive phenotypes [25]. Future work will address cell-type specificity and optimize combinatorial strategies to maximize clinical impact.

## Conclusion

Our study elucidates a novel, non-canonical role for EIF3B in LSCC progression, positioning it as a dual-function oncoprotein with translational and proteostatic roles. By integrating mechanistic, preclinical, and translational insights, we provide a framework for developing EIF3B-centric therapies. Future work will address cell-type specificity and optimize combinatorial strategies to maximize clinical impact.

## Materials and methods

### Bioinformatics analysis

Comprehensive analysis of long non-coding RNA and mRNA expression profiles in advanced LSCC was identified [26]. LSCC tissue samples (*n* = 9) and paired non-tumor tissue samples (*n* = 9) were selected to screen significant differentially expressed genes (DEGs) through the Gene Expression Omnibus (GEO) database (https://www.ncbi.nlm.nih.gov/geo/query/acc.cgi?acc=GSE84957). The expression level of EIF3B in LSCC was revealed by GEO database. Moreover, we performed expression profiling and survival analysis based on the Cancer Genome Atlas (TCGA) database of 111 tumor and 12 normal samples for LSCC.

### Tissue chip collection and immunohistochemical (IHC) staining

The LSCC patients who participated in the study signed a written informed consent, and the study protocol was approved by the Ethics Committee of Peking University People’s Hospital. Moreover, the tumor tissues (*n* = 123) and adjacent tissues (*n* = 41) of LSCC patients used in this study are accompanied with detailed pathological data. Xylene was used for paraffin section dewaxing 15 min per time, and 100% alcohol for hydration 10 min. PBS-H_2_O_2_ with 0.1% Tween 20 was added. After 20 min, the slides were washed three times with PBS, 5 min each time. Citric acid buffer was added for antigen retrieval, with heating at 120 °C for 20 min. After washing, the slides were incubated with anti-EIF3B (1:100, Abcam, Cat # ab124778) overnight. Afterwards, biotinylated secondary antibody IgG (1: 400, Abcam, USA, Cat # ab6721) was added to continue incubation for 1 h. As soon as the sections developed, they were immersed in deionized water and stained with DAB followed by hematoxylin for visualization. All tissues were pictured with microscopic and viewed with ImageScope and CaseViewer. IHC scores were determined by staining percentage scores (classified as: 1 (1–24%), 2 (25–49%), 3 (50–74%), 4 (75–100%)) and staining intensity scores (scored as 0: signal less color, 1: brown, 2: light yellow, 3: dark brown). To distinguish the high or low expression of EIF3B between LSCC tissues and para-cancerous tissues, the median was selected as cut off-value to reduce the impact of outliers.

### Cell culture

Human nasopharyngeal epithelial cells NP-69 and LSCC cell lines AMC-HN-8, TU212, TU686 were obtained from Cell Bank of the Chinese Academy of Sciences (Shanghai, China). All of the cells were cultured at 37 °C in Dulbecco’s modified Eagle’s medium (DMEM) and supplemented with 10% fetal bovine serum (FBS) in the 5% CO_2_ atmosphere. These cell lines were identified by STR profiling and detected for mycoplasma contamination.

### Lentivirus construction and cell transfection

Short hairpin RNAs (shRNA) of human EIF3B, MAP2K2, and control sequence (shCtrl) were designed for knockdown or overexpression experiments. shEIF3B-1: 5’-GGGAGAGAAATTCAAGCAAA-3’, shEIF3B-2: 5’-GAGTGGGATATTCCAGAGAAA-3’, shEIF3B-3: 5’-GAAGAAAGAGCGAGATGGACA-3’; shMAP2K2-1: 5’-GCTGAAAGAGGCCAAGAGGAT-3’, shMAP2K2-2: 5’-CCGAGAGAAGCACCAGATCAT-3’, shMAP2K2-3: 5’-CGAGGCAAACCTGGTGGACCT-3’; shCtrl: 5’-CCGGGAAGAAAGAGCGAGATGGACACTCGAGT GTCCATCTCGCTCTTTCTTCTTTTTG-3’. The small interfering RNAs target sequences were inserted into lentiviral vector LV-002 or LV-007 (Shanghai Yibeirui, China) using the T4 DNA ligase enzyme (NEB). After AMC-HN-8 and Tu212 cells (2 × 10^5^ cells/well) were seeded in a six-well plate for 24 h culture, they were transfected with 100 µL BR-V-108 vector (1 × 10^7^ TU/well) additive with green fluorescent protein (GFP). After cultured for 72 h, the fluorescence was observed by microscope. The cells with recombinant lentiviruses that knocked down EIF3B, MAP2K2 were named shEIF3B, shMAP2K2, respectively. The recombinant lentiviruses overexpressed EIF3B was named as EIF3B, simultaneous EIF3B upregulation + MAP2K2 downregulation was EIF3B+shMAP2K2 group. The shCtrl, Control, and NC(OE + KD) were the corresponding control groups.

### RNA extraction and qPCR

RNA was extracted from cells using Trizol reagent (Invitrogen, CA, USA) according to the manufacturer’s instructions. RNA (2.0 μg) was transcribed into cDNA with M-MLV RT kit (Promega Corporation, Madison, Wisconsin, USA). The qPCR was performed on the Roche Light Cycler® 96 real-time PCR platform, and mRNA level expression of target gene was quantified using the 2^−ΔΔCT^ method. GAPDH was used as the internal controls for the quantification of target gene (primer sequences were detailed in Table S1).

### Western blotting (WB)

After AMC-HN-8 and Tu212 cells transfection for 5 days, RIPA buffer (Millipore) was used to collect proteins, and protein concentration was measured using BCA protein kit (Takara, Otsu, Japan). Proteins were denatured in Sodium dodecyl sulfate (SDS) sample buffer, followed by separating on SDS-polyacrylamide electrophoresis (PAGE) gel using electrophoresis. After that, they were transferred onto polyvinylidene difluoride (PVDF) membrane, and incubated with primary antibodies (Table S2) overnight at 4 °C. Membranes were incubated with horseradish peroxidase (HRP)-conjugated secondary antibodies IgG (Goat Anti-Rabbit) at room temperature for 1 h. After the membranes were washed in TBST, and the blots were visualized by enhanced chemiluminescence ECL kit (Thermo Fisher Scientific, NY, USA).

### Co-Immunoprecipitation (Co-IP) assay

After AMC-HN-8 and Tu212 cells transfection for 5 days, RIPA buffer (Millipore) was used to collect proteins, and protein concentration was measured using BCA protein kit (Takara, Otsu, Japan). The protein was co-incubated with the required antibody overnight and then co-immunoprecipitated with 20 μL beads. The protein-antibody-beads complex was eluted, and SDS-PAGE electrophoresis was carried on. After that, they were incubated with the corresponding first antibody and second antibody to analyze the results of the blots by enhanced chemiluminescence ECL kit (Thermo Fisher Scientific, NY, USA).

### MTT assay

After AMC-HN-8 and Tu212 cells transfection for 5 days, they at a density of 2 × 10^3^ cells/well were seeded in a 96-well plate for 24 h culture. Total 20 µL of 5 mg/mL MTT (Genview, Beijing, China, Cat. # JT343) was added for 4 h, DMSO (Shanghai Test Chemical Reagent Co., Ltd., Shanghai, China) was added to dissolve the methylaz crystals. Finally, the absorbance at OD490 nm was quantified through multifunctional microplate reader (Tecan Group, Ltd., Mannedorf, Switzerland).

### Celigo cell counting assay

After AMC-HN-8 cells transfection for 5 days, they at a density of 2 × 10^3^ cells/well were seeded in a 96-well plate for 48 h culture. Next day, the Celigo test reading board was carried out at the same time every day and monitored continuously for 5 days. By observing the green fluorescence in the pore plate scanned each time, the number of cells was estimated, and the cell proliferation curve was drawn.

### Colony formation assay

After AMC-HN-8 and Tu212 cells transfection for 5 days, they at a density of 2 × 10^3^ cells/well were seeded into 6-well plate for 8 days, and the culture medium was exchanged every 3 days. Following, the cell clones were first fixed with 4% paraformaldehyde and then stained by Giemsa, the clones were photographed with a digital camera, and colony forming rate was evaluated.

### Detection of cell apoptosis

After AMC-HN-8 and Tu212 cells transfection for 5 days, they at a density of 2 × 10^3^ cells/well were inoculated in a 6-well plate and harvested until cell density reached 85%. Following, 200 μL 1×binding buffer was added to the resuspended cells. After the cells were precipitated by pre-cooled D-Hanks (PH = 7.2 ~ 7.4), Annexin V-APC and PI were added at 37 °C without light for 10 min. Cell apoptosis rate was calculated by flow cytometry (FACS) in 3 randomly selected visual fields.

### Transwell assay

After AMC-HN-8 and Tu212 cells transfection for 5 days, they were seeded into well-hydrated chamber (3422 Corning) for 24 h. The internal chamber contained 100 μL/well cells without FBS, and external chamber 600 μL/well cells was filled with medium supplemented with 30% FBS. Next, migrated cells were fixed by 4% formaldehyde and stained by Giemsa. Finally, the cells were photographed, and analyzed the migration rate.

### Wound-healing assay

After AMC-HN-8 and Tu212 cells transfection for 5 days, they at a density of 5×10^4^ cell/well (100 μL/well) were added to the 12-well plates for incubation until cell fusion reached 80%. Following, scratch was made by 96-wounding replicator (VP scientific) across the cell monolayer. Subsequently, photographs of the wound were captured by a fluorescence microscope at pre-set time points (0 h, 24 h and 48 h) and migration percentage was evaluated utilizing Image Pro Plus.

### RNA sequencing and ingenuity pathway analysis (IPA)

The screening of downstream target genes was identified (shCtrl vs shEIF3B) using RNA sequencing and Ingenuity Pathway Analysis (IPA). DEGs were determined between the control and experimental groups based on thresholds of |Fold Change | ≥ 2.0 and FDR < 0.05. In addition, IPA (Qiagen, Hilden, Germany) based on all DEGs to analyze rich functional annotations.

### Subcutaneous xenograft tumor mouse model

The mice experiment was approved and performed according to the animal protection guidelines and Committee of Peking University People’s Hospital. The BALB/c nude mice were four-week-old male, which were purchased from Shanghai Shrek Experimental Animal Co., Ltd to construct mice xenograft model. About 4 × 10^6^ AMC-HN-8 cells were subcutaneous injected into each mouse. The fluorescence imaging was performed using IVIS Spectrum Imaging System (Perkin Elmer), images were collected and analyzed. After that, tumor volume and mice weight were collected 2-3 times per week. The tumor volume was calculated through π/6 × L × W × W, where L represented the long diameter and W represented the short diameter. Finally, the mice were sacrificed, the tumor was removed for photographing and weighing. The mice tissue sections were incubated with Ki67 (1:200, Abcam, USA, Cat # ab16667), stained with DAB and hematoxylin. Finally, images were collected by optical microscope and analyzed.

### Statistical analysis

Statistical analyses were performed using SPSS 19.0 with GraphPad Prism 8.0 software, and data were presented as the mean ± standard deviation. The independent Student’s *t*-test was used to analyze the statistical significance between two groups, and P < 0.05 was considered statistically significant. Differences between different groups were assessed using the independent Student *t*-test, and Onaway ANOVA. The correlation between EIF3B and the clinicopathological characteristics of patients with LSCC was analyzed using χ^2^ or Fisher’s exact test.

## Supplementary information

Supplementary Tables

Supplementary Figures

WB images
