# Supplementary material for: EIF3B stabilizes MAP2K2 to activate the ERK pathway and promote the progression of laryngeal squamous cell carcinoma
Source: Cell Death Discov. 2025 Jul 21;11:333. doi: 10.1038/s41420-025-02634-2 (PMC12280010; doi:10.1038/s41420-025-02634-2)
Supplement: Supplementary file 1 — Supplementary Tables [file 41420_2025_2634_MOESM1_ESM.docx]

Table S1 Primers used in qPCR

| Gene | Forward primer sequence (5’-3’) | Reverse primer sequence (5’-3’) |
| --- | --- | --- |
| GAPDH | TGACTTCAACAGCGACACCCA | CACCCTGTTGCTGTAGCCAAA |
| EIF3B | CCTGAAGAGGATGGGAAGACA | AAGAGGTTGACCCGGAATG |
| CDK5 | GCACAAGAACATCGTCAGGC | AGCTCCCCATTCTTTACAATCTCA |
| CCNE1 | CTGGATGTTGACTGCCTTGAA | CGCACCACTGATACCCTGAAA |
| PPP2R5D | AGTTTGACCCAGAGGAAGATGA | GGAAATCAGGAGACTCAAGGAA |
| CDC25C | GAAACTGGTCACCTGGATTCTTC | AACCATTCGGAGTGCTACAAAG |
| EGR1 | CACCTGACCGCAGAGTCTTTT | TGGTTTGGCTGGGGTAACTG |
| FOS | CAGACTACGAGGCGTCATCC | TCTGCGGGTGAGTGGTAGTA |
| MAPK3 | ATTGTGCAGGACCTGATGGA | ACGTTGGCGGAGTGGATGTA |
| MAPKAP1 | ATCTCCTGGTCTGACATCCAAA | TTCTTCGCTTCACTGCCTTC |
| SFN | TGGACAGCCACCTCATCAA | GCGGTAGTAGTCACCCTTCAT |
| TCF3 | GAAGCAGCAGCACGTTTGGT | AAGGAGGATGCAGATGGGAG |
| TGFB1 | CAATTCCTGGCGATACCTCA | AAGGCGAAAGCCCTCAAT |
| YWHAZ | GTAGGAGCCCGTAGGTCA | TCGAGCCATCTGCTGTTT |
| MAP2K2 | TTGTGAACGAGCCACCTCC | AGGTGTGGTTTGTGAGCATCT |

Table S2 Antibody information for WB

| Name | Size  (kDa) | Diluted  multiples | Source | Company | Number |
| --- | --- | --- | --- | --- | --- |
| EIF3B | 116 | 1:2000 | Rabbit | abcam | ab124778 |
| CD133 | 97 | 1:1000 | Rabbit | abcam | ab19898 |
| MELK | 75 | 1:1000 | Rabbit | abcam | ab108529 |
| SOX2 | 34 | 1:1000 | Rabbit | abcam | ab92494 |
| TLR4 | 95 | 1:1000 | Mouse | Santa cruz | SC-293072 |
| MCP-1 | 25 | 1:1000 | Rabbit | abcam | ab25124 |
| MIP-1α | 11 | 1:1000 | Rabbit | abcam | ab25128 |
| AKT | 60 | 1:2000 | Mouse | systems | MAB2055-sp |
| p-AKT | 60 | 1:500 | Rabbit | systems | AF887-sp |
| mTOR | 289 | 1:3000 | Mouse | san eagle | 66888-1-Ig |
| p-mTOR | 289 | 1:1000 | Rabbit | CST | 5536 |
| PI3K | 85 | 1:500 | Goat | systems | AF2998-SP |
| PIK3CA | 110 | 1:1000 | Rabbit | abcam | ab40776 |
| CCND1 | 36 | 1:1000 | Rabbit | CST | 2978 |
| CDK6 | 37 | 1:1000 | Rabbit | abcam | ab151247 |
| EGR1 | 60 | 1:1000 | Rabbit | bioss | bs-1076R |
| MAP2K2 | 44 | 1:5000 | Mouse | PTG | 67410-1-Ig |
| MAPK3 | 42,44 | 1:2000 | Rabbit | R&D | AF1576-sp |
| MAPKAP1 | 59 | 1:500 | Rabbit | PTG | 15463-1-AP |
| RPS6KA1 | 83 | 1:500 | Rabbit | PTG | 16463-1-AP |
| GAPDH | 37 | 1:3000 | Rabbit | Bioworld | AP0063 |

| Secondary antibody | Dilution |  | Company | Catalog No. |
| --- | --- | --- | --- | --- |
| HRP Goat Anti-Mouse IgG | 1:3000 |  | Beyotime | A0216 |
| HRP Goat Anti- Rabbit IgG | 1:3000 |  | Beyotime | A0208 |
