# Supplementary material for: EIF3B stabilizes MAP2K2 to activate the ERK pathway and promote the progression of laryngeal squamous cell carcinoma
Source: Cell Death Discov. 2025 Jul 21;11:333. doi: 10.1038/s41420-025-02634-2 (PMC12280010; doi:10.1038/s41420-025-02634-2)
Supplement: Supplementary file 2 — Supplementary Figures [file 41420_2025_2634_MOESM2_ESM.docx]

Supplementary Figures


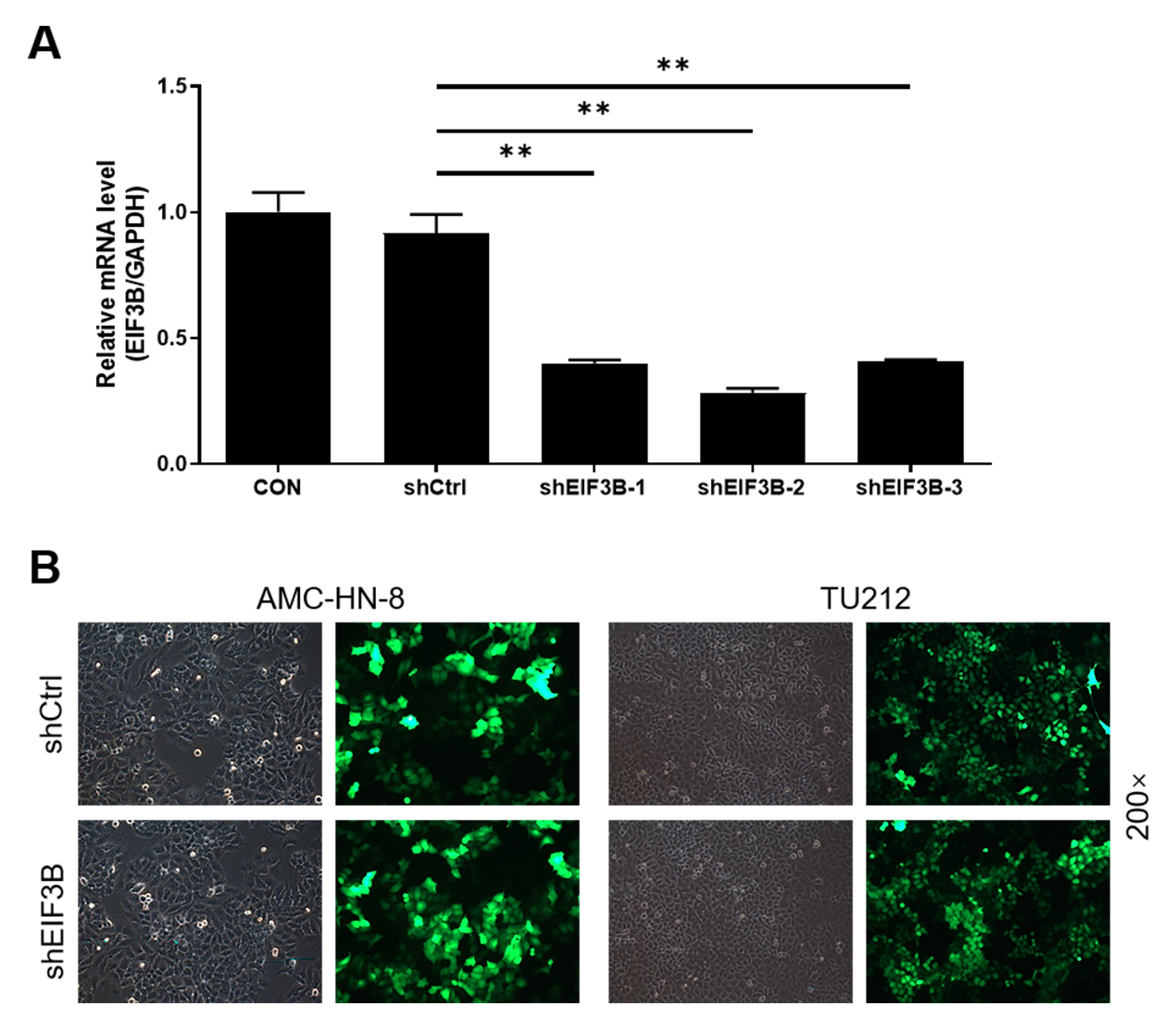


Figure S1. Construction of EIF3B knockdown LSCC cell model. (A) Effectively targeted EIF3B sequences were screened by qPCR. (B) Transfection efficiencies of EIF3B knockdown for cells were evaluated by expression of green fluorescent protein 72 h post-infection. The representative images were selected from at least 3 independent experiments. The data was presented as the mean ± SD (n = 3). *P<0.05, **P<0.01.


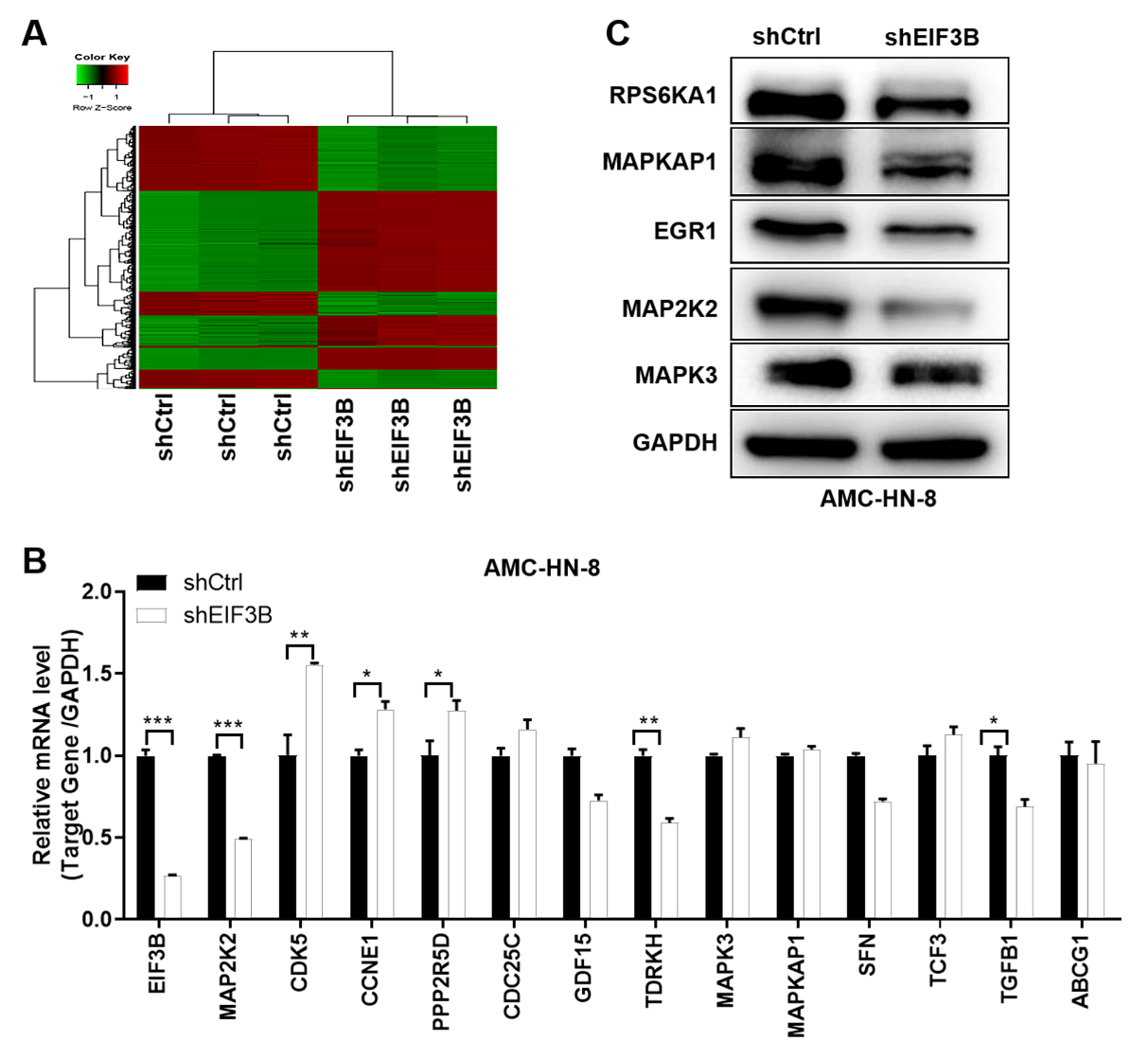


Figure S2. The effects of EIF3B knockdown and the control cell AMC-HN-8 on the expression of downstream genes. (A) A Prime View Human Gene Expression Array was performed to identify the DEGs between shEIF3B and shCtrl groups of AMC-HN-8 cells. (B-C) The expression of these top-ranked DEGs in AMC-HN-8 cells (shCtrl vs shEIF3B) were further detected by PCR (B) and WB (C). The representative images were selected from at least 3 independent experiments. The data was presented as the mean ± SD (n = 3). *P<0.05, **P<0.01, ***P<0.001.


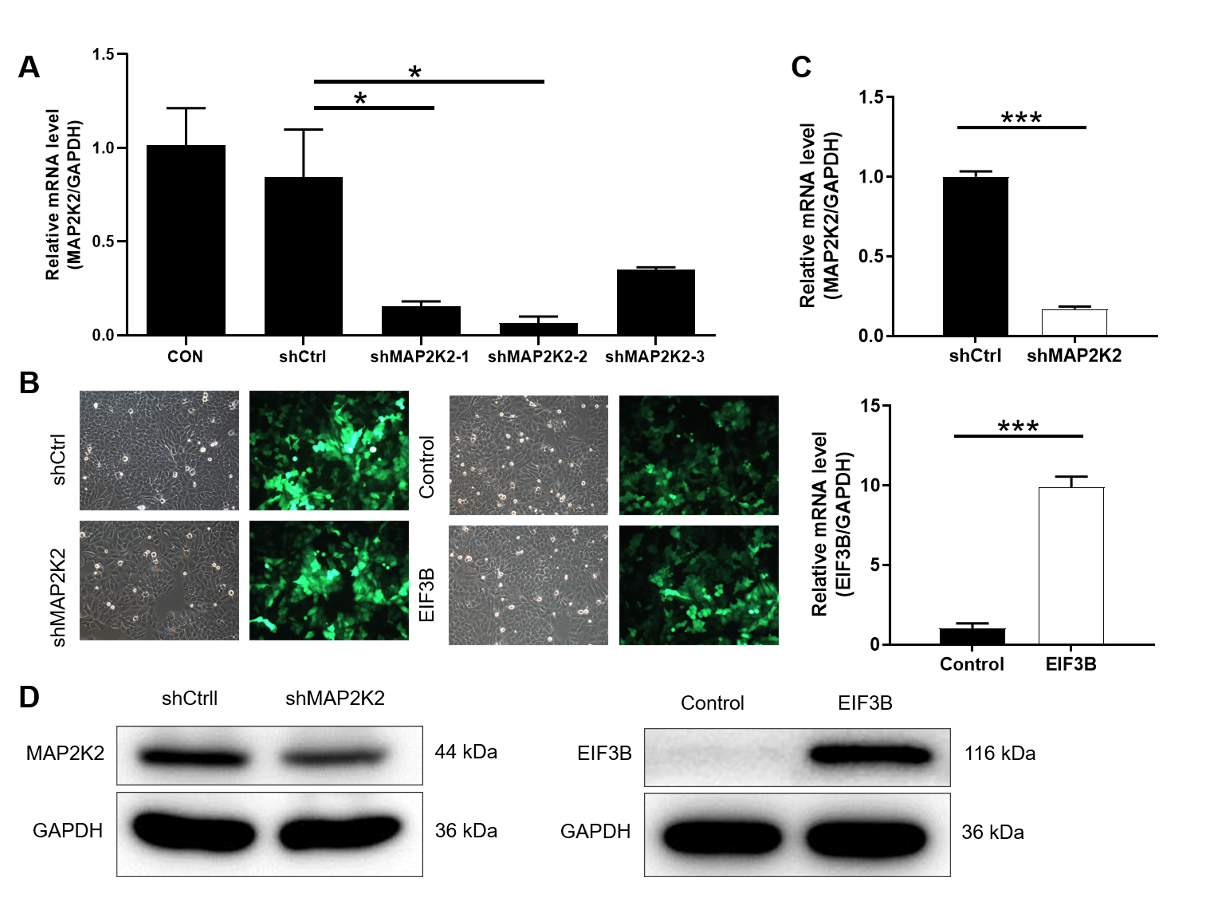


Figure S3. Construction of EIF3B overexpression and MAP2K2 knockdown LSCC cell models. (A) Effectively targeted MAP2K2 sequences were screened by qPCR. (B) Transfection efficiencies of MAP2K2 knockdown for cells were evaluated by expression of green fluorescent protein 72 h post-infection. (C-D) Expression of EIF3B and MAP2K2 in AMC-HN-8 cells was verified by qPCR and WB analysis. The representative images were selected from at least 3 independent experiments. The data was presented as the mean ± SD (n = 3). *P<0.05, ***P<0.001.


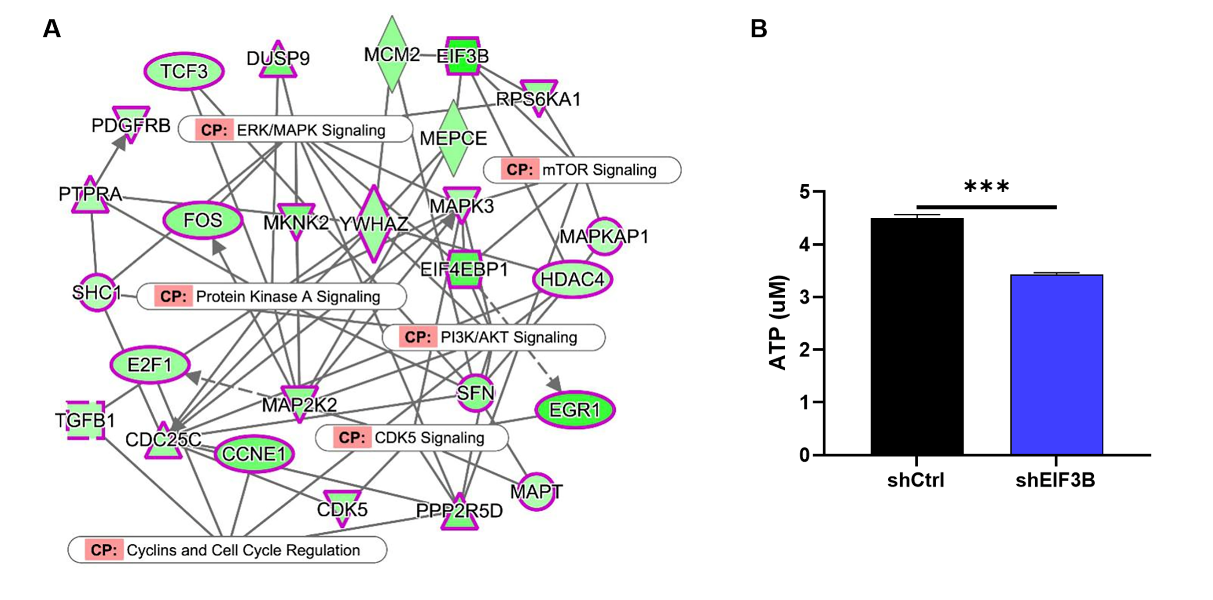


Figure S4. Exploration on the downstream regulatory network of EIF3B in LSCC. (A) Regulation network diagram of EIF3B and MAP2K2 interaction. (B) The phosphorylation level of ERK in EIF3B knockdown and control LSCC cells was detected by ELISA.
