# Supplementary material for: EIF3B stabilizes MAP2K2 to activate the ERK pathway and promote the progression of laryngeal squamous cell carcinoma
Source: Cell Death Discov. 2025 Jul 21;11:333. doi: 10.1038/s41420-025-02634-2 (PMC12280010; doi:10.1038/s41420-025-02634-2)

AMC-HN-8

Figure 2C

EIF3B

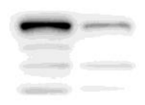

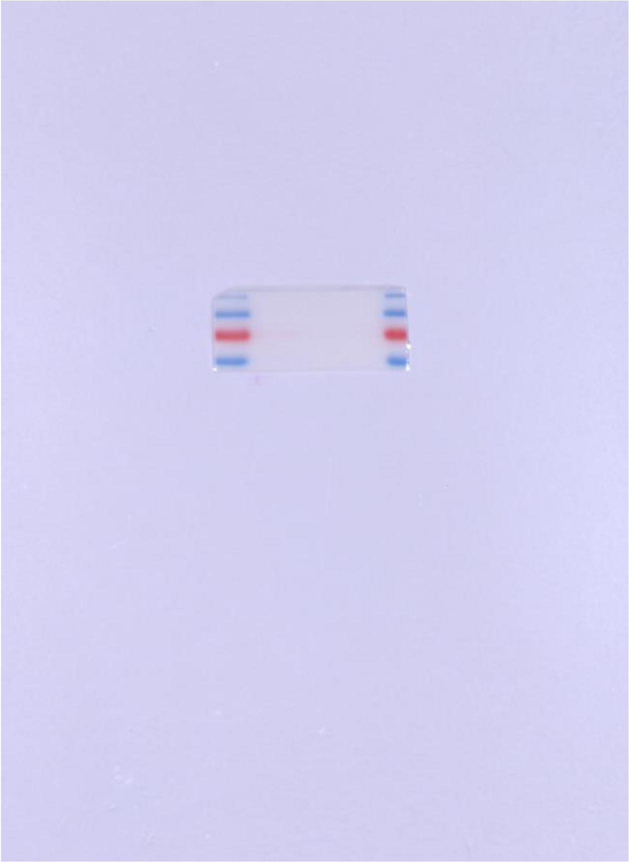

GAPDH

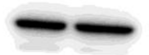

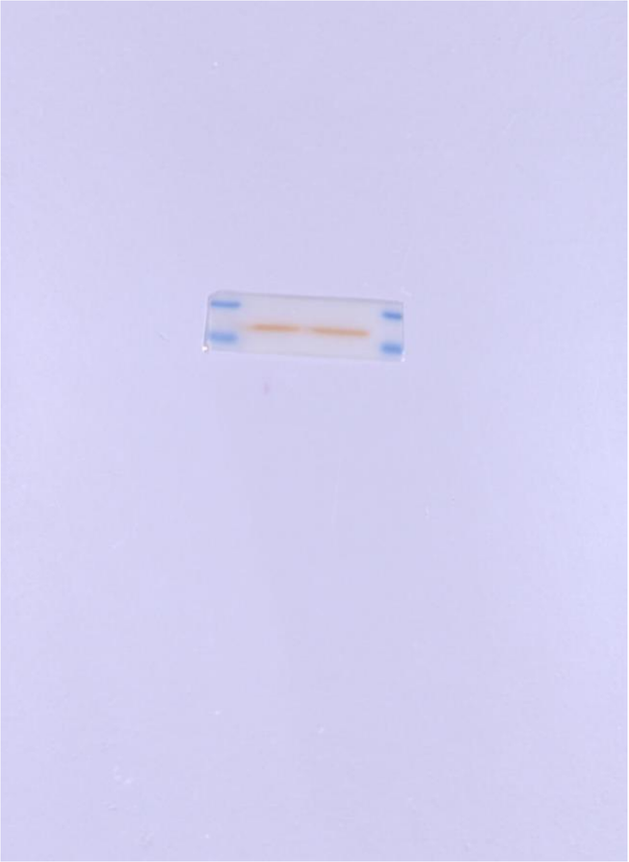

TU212

Figure 2C

EIF3B

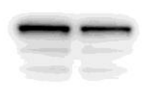

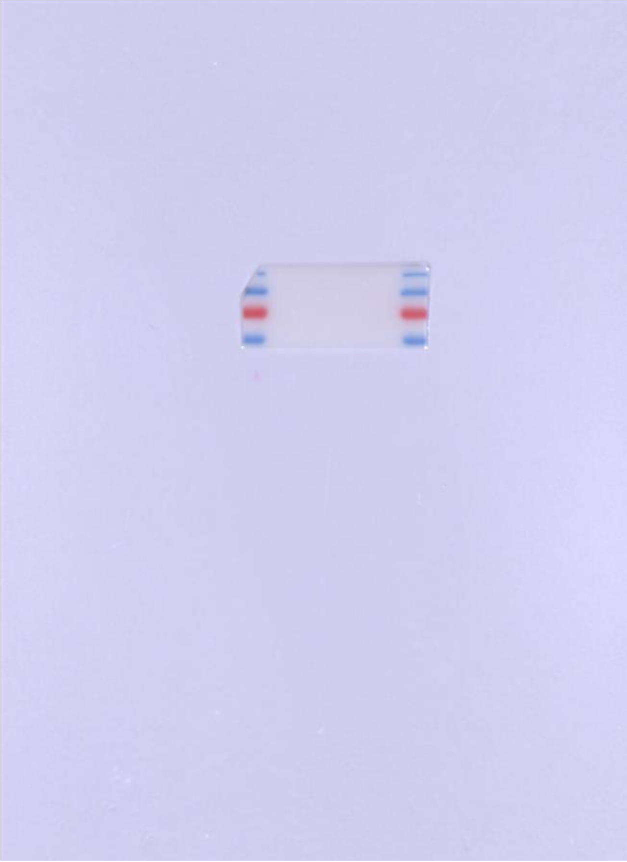

GAPDH

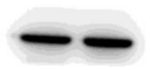

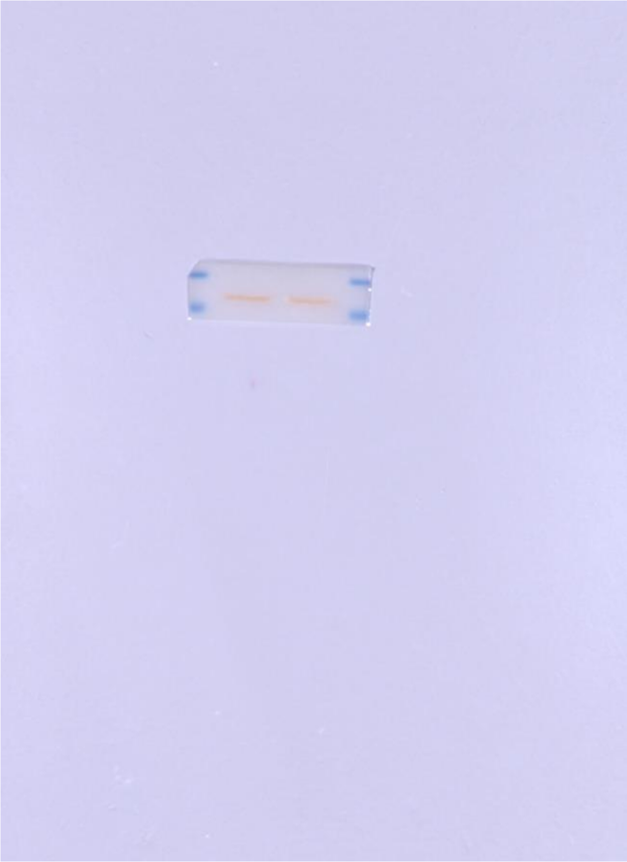

AMC-HN-8

Figure 3A

EIF3B

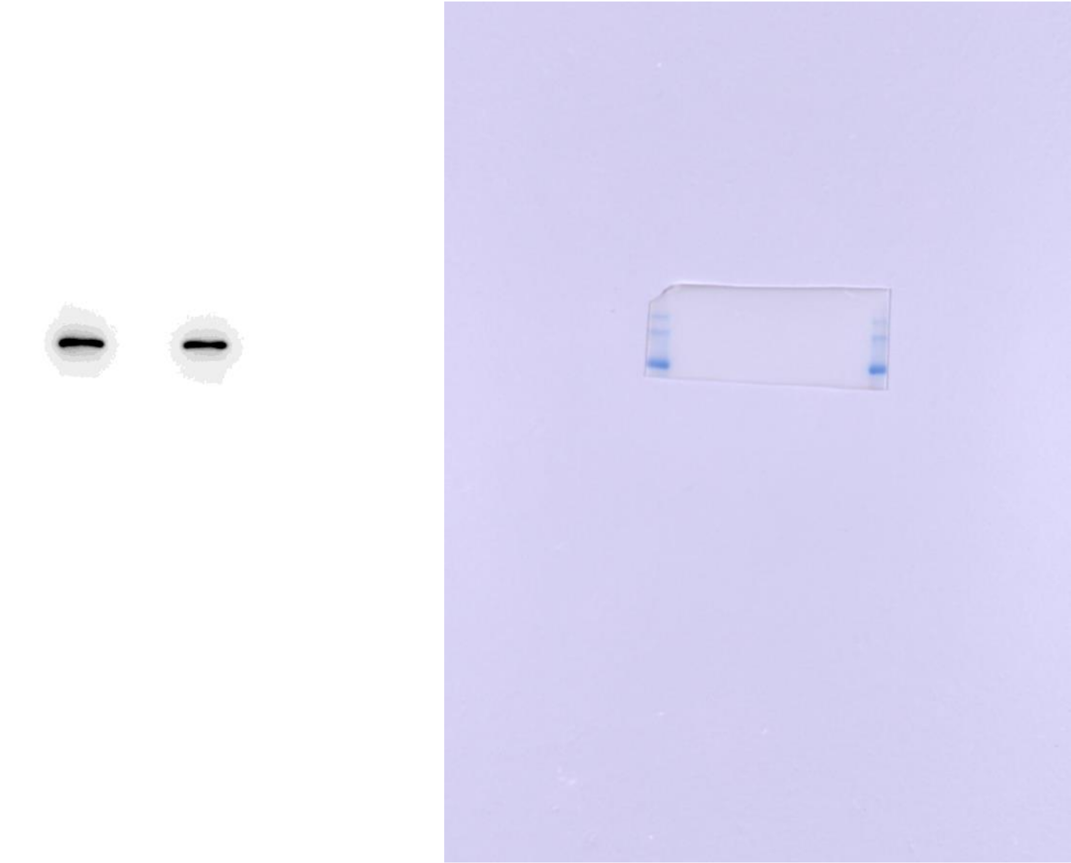

MAP2K2

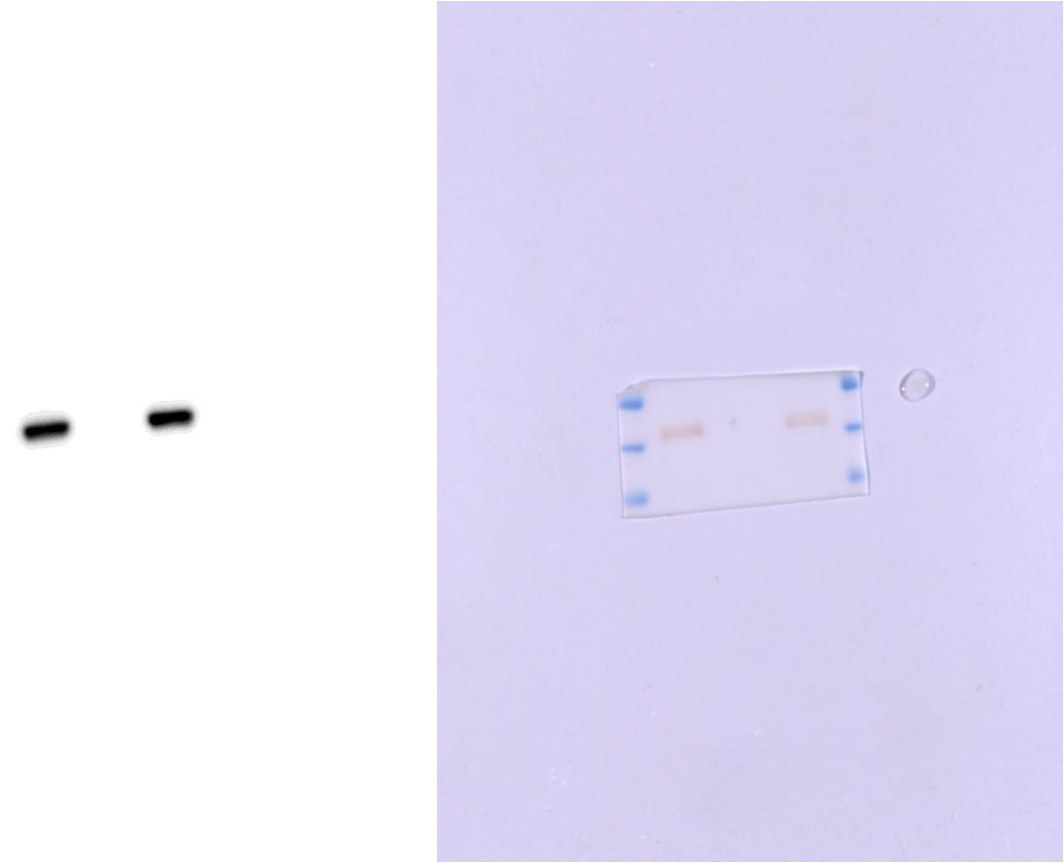

TU212

Figure 3A

EIF3B

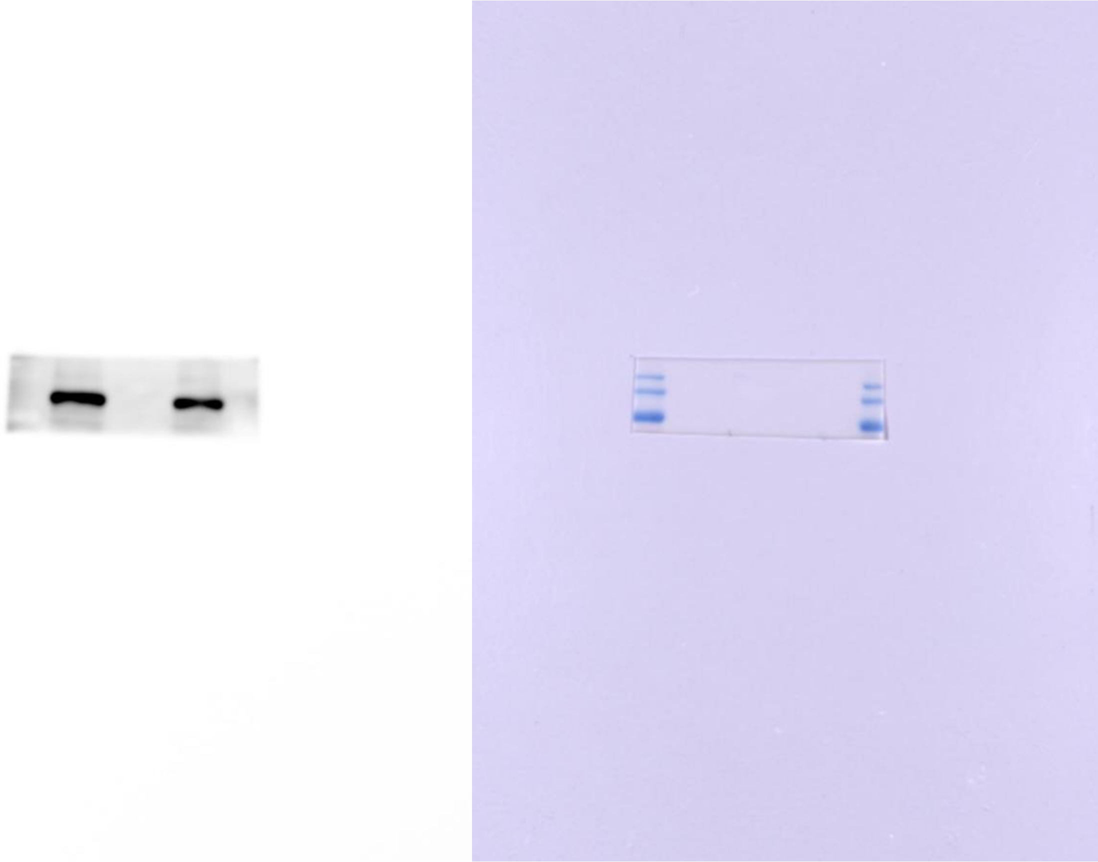

MAP2K2

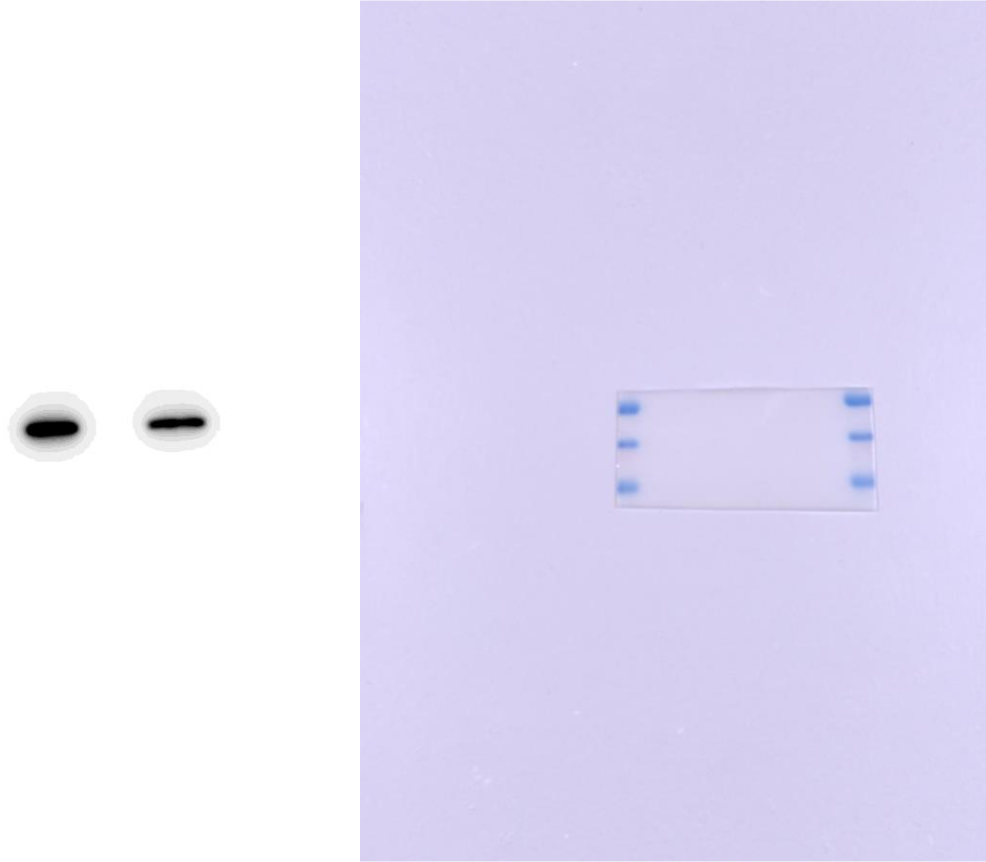

FLAG

Figure 3B

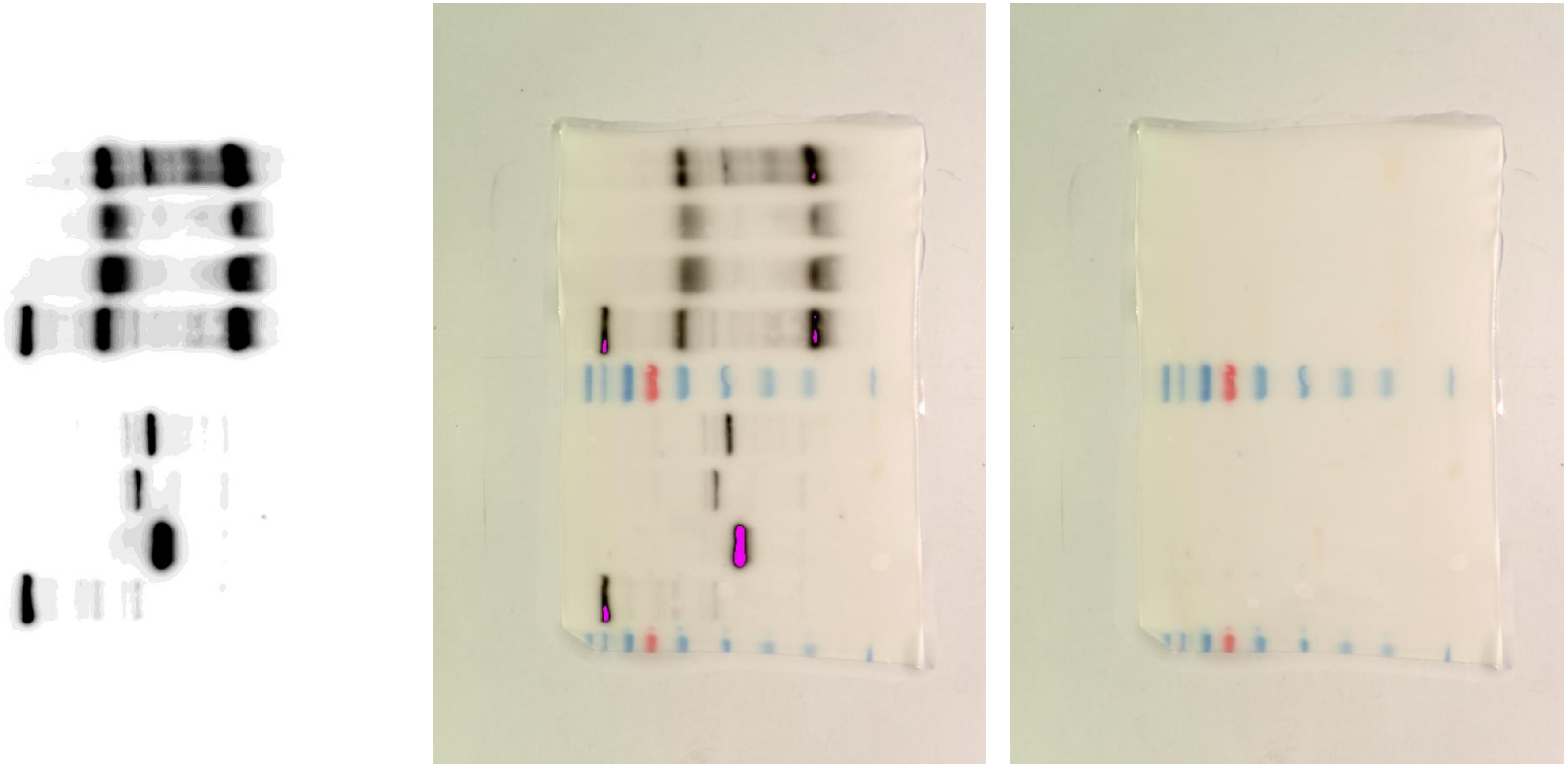

Figure 3B

HA

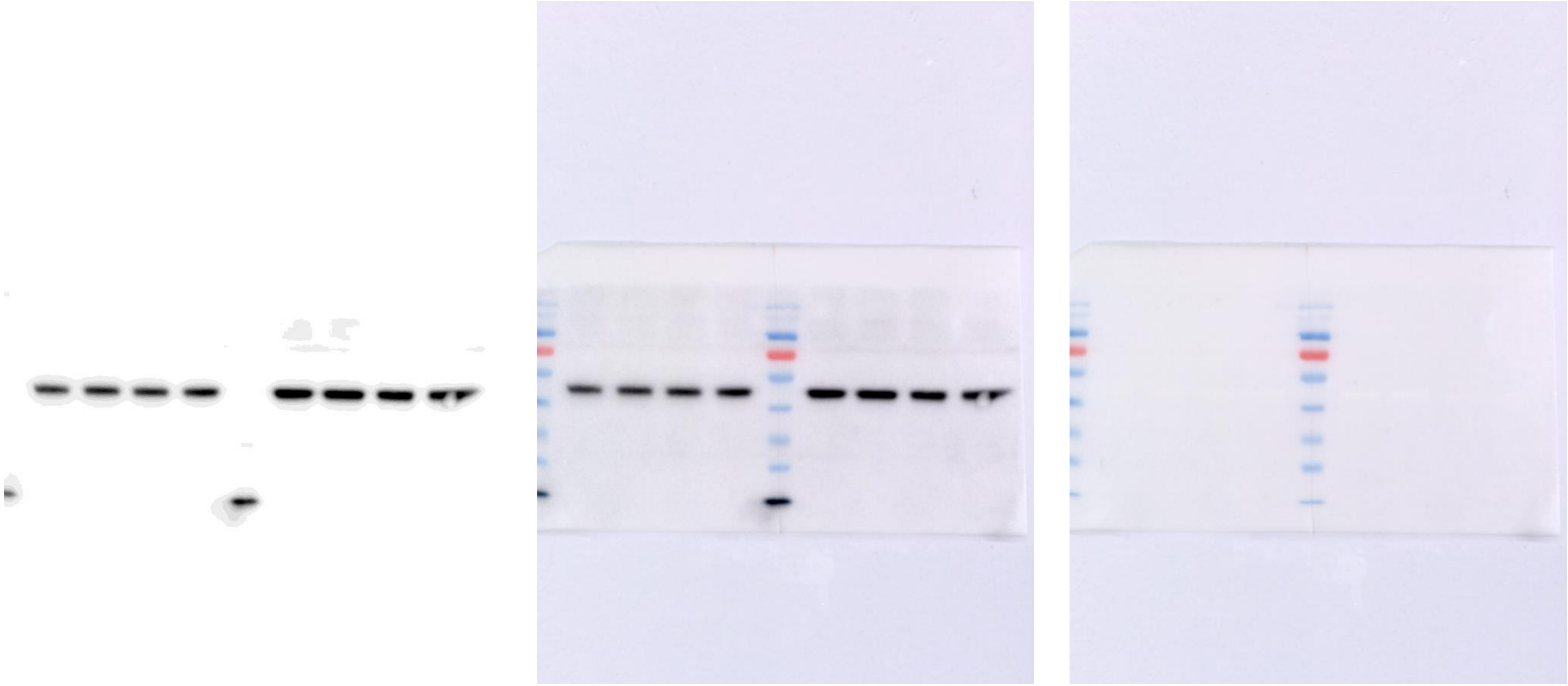

AMC-HN-8

Figure 3C

MAP2K2-shCtrl

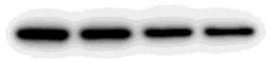

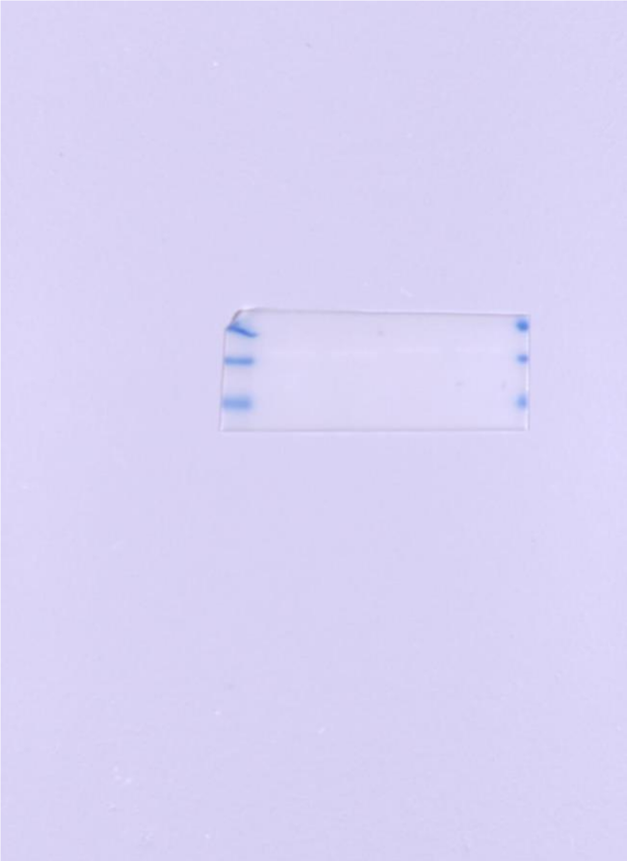

MAP2K2-shEIF3B

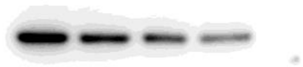

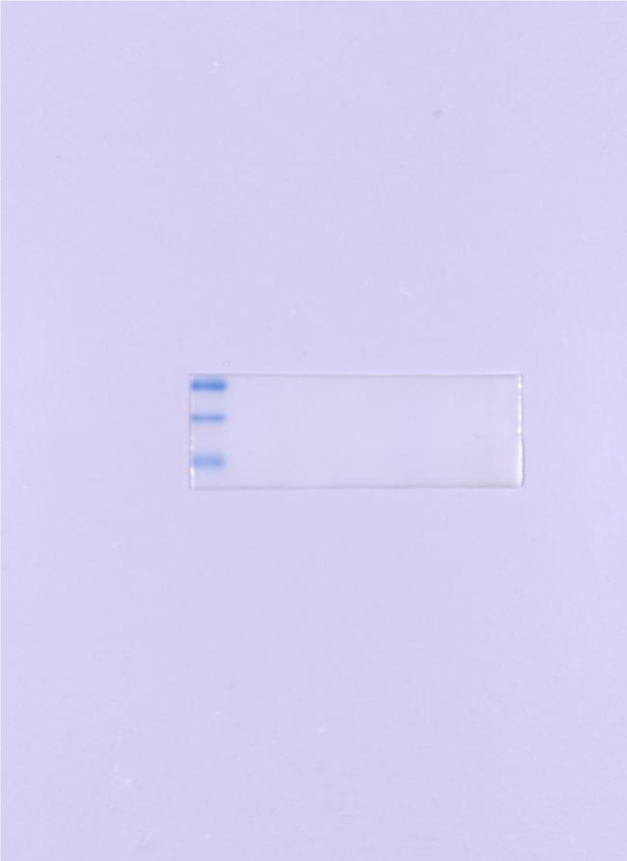

AMC-HN-8

Figure 3C

GAPDH-shCtrl

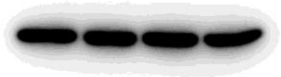

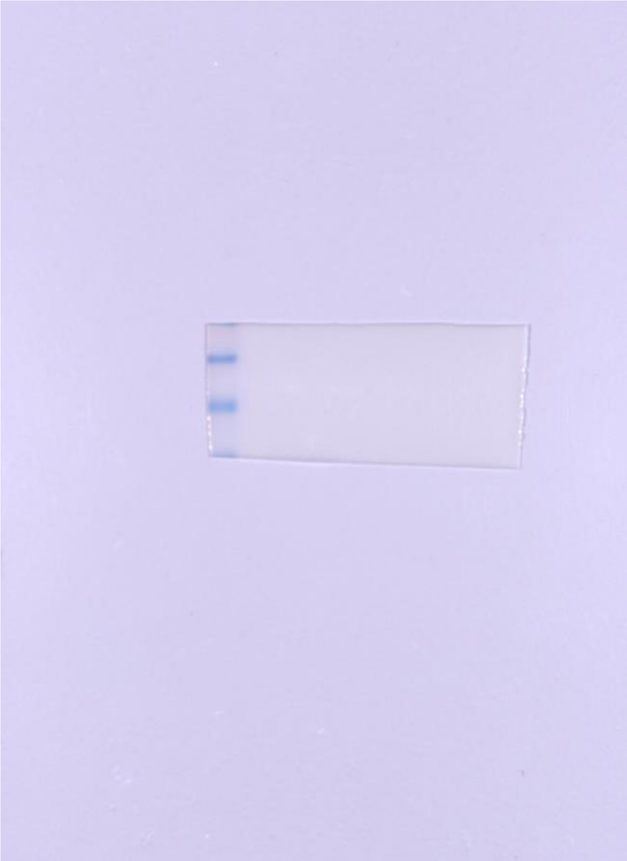

GAPDH-shEIF3B

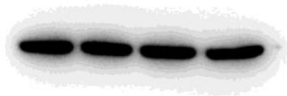

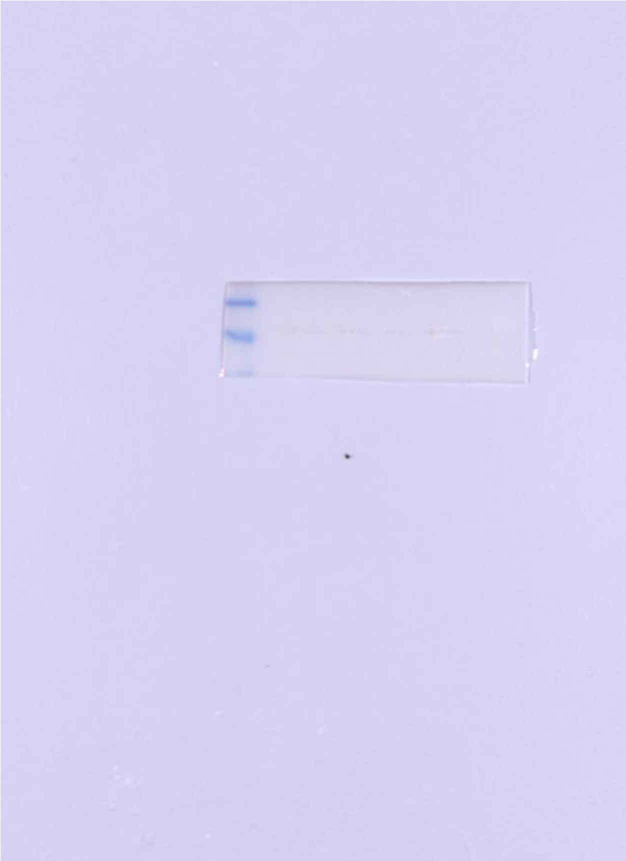

TU212

Figure 3C

MAP2K2-shCtrl

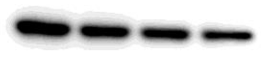

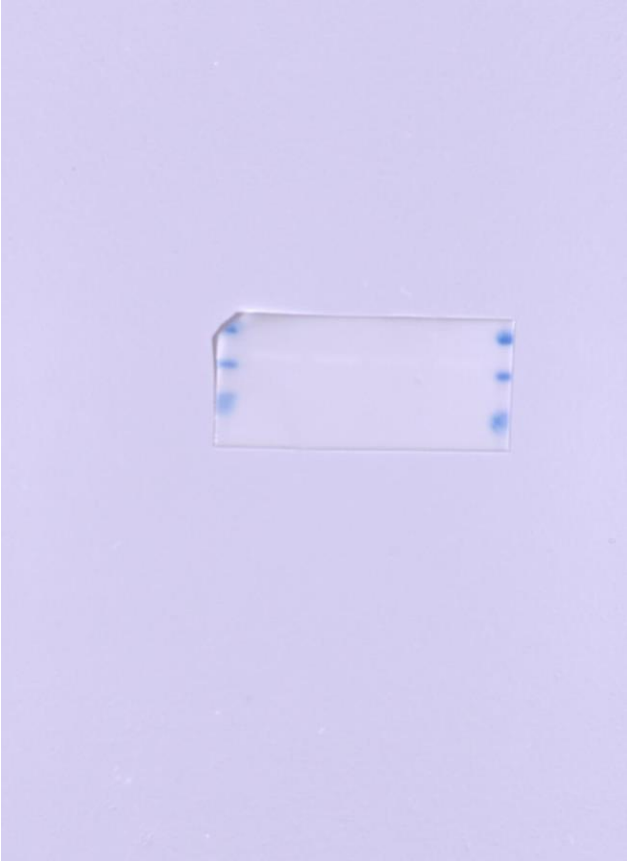

MAP2K2-shEIF3B

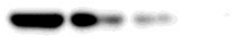

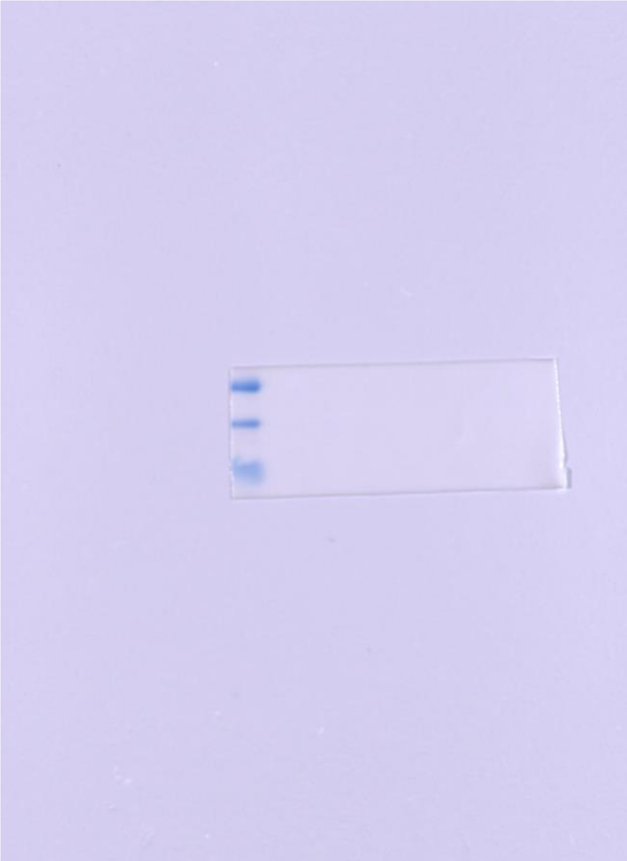

TU212

Figure 3C

GAPDH-shCtrl

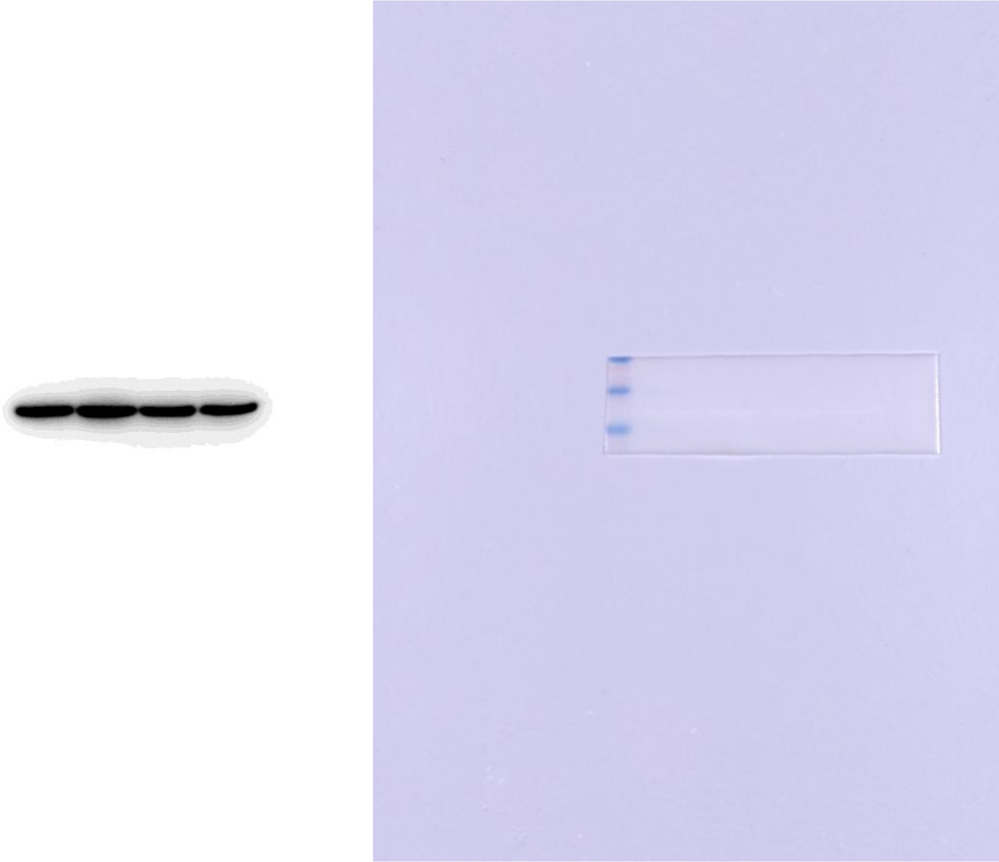

GAPDH-shEIF3B

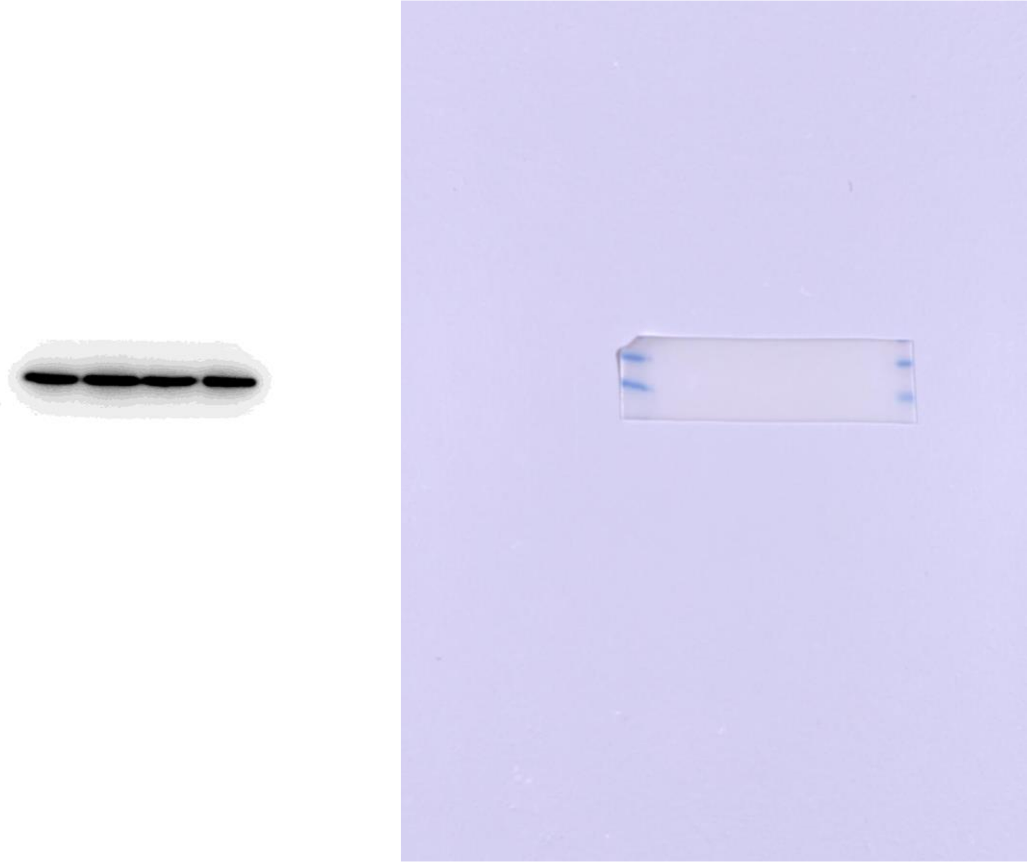

Figure 3D

AMC-HN-8

MAP2K2

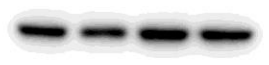

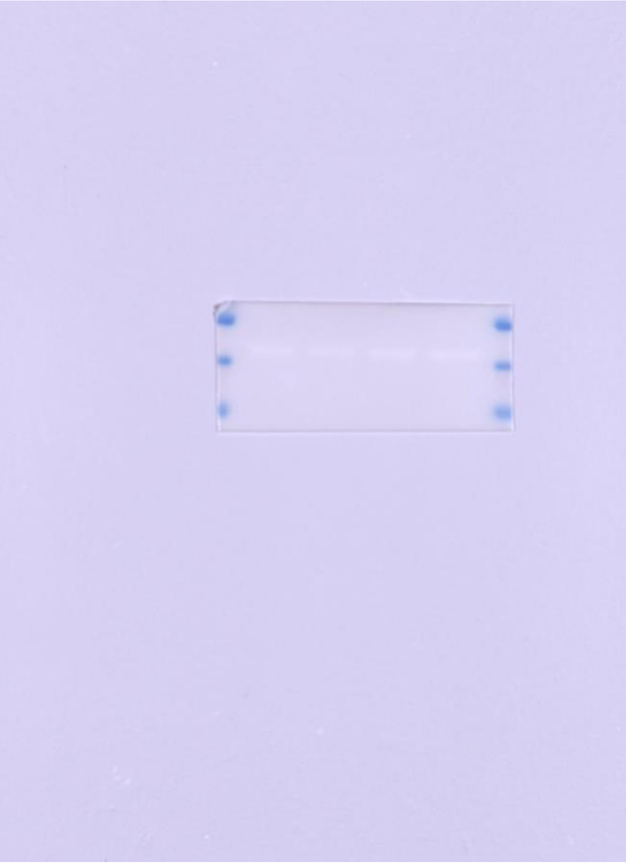

GAPDH

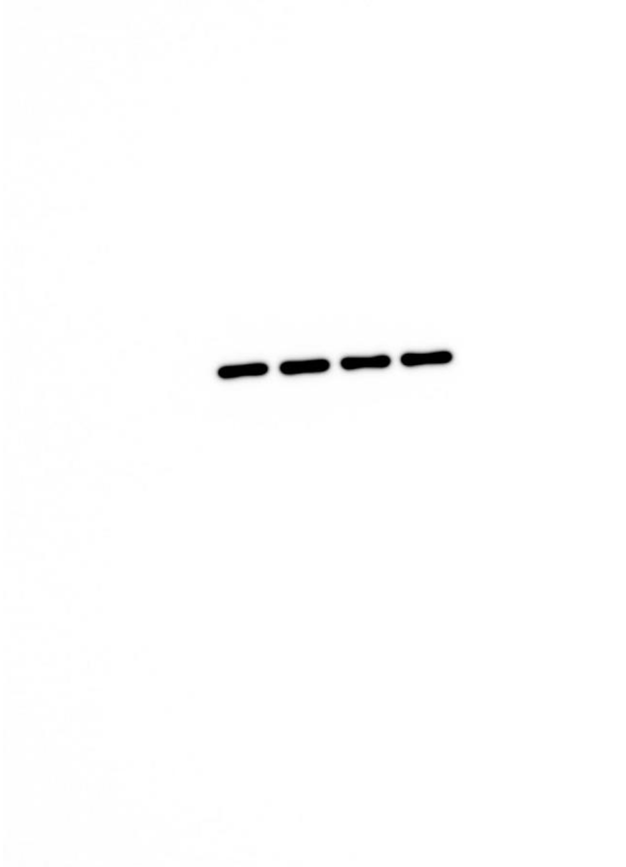

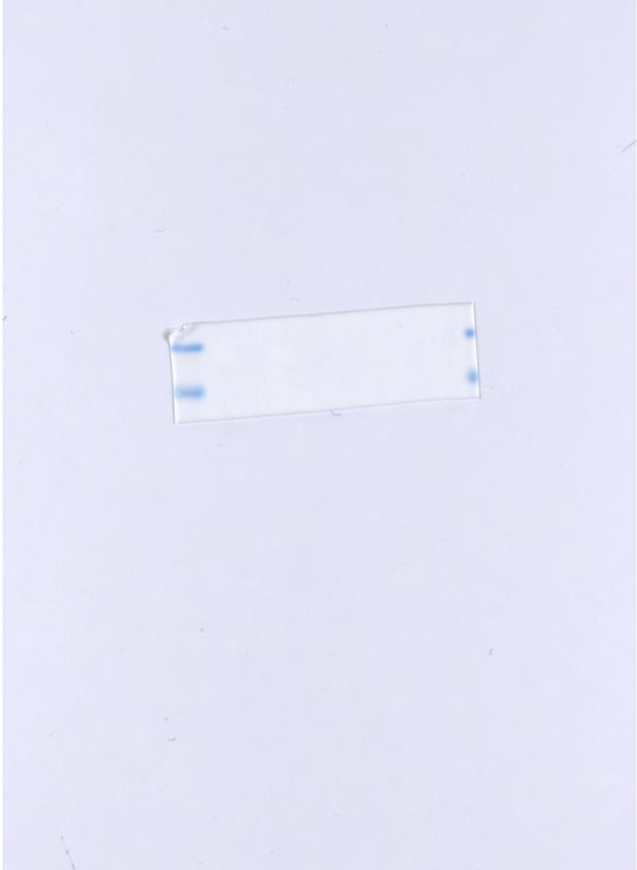

TU212

Figure 3D

MAP2K2

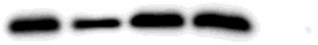

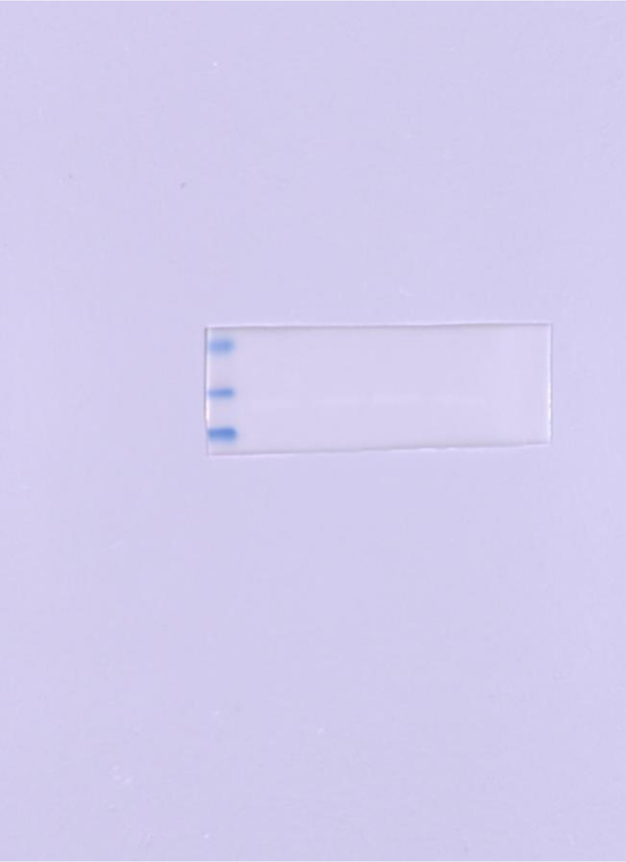

GAPDH

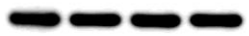

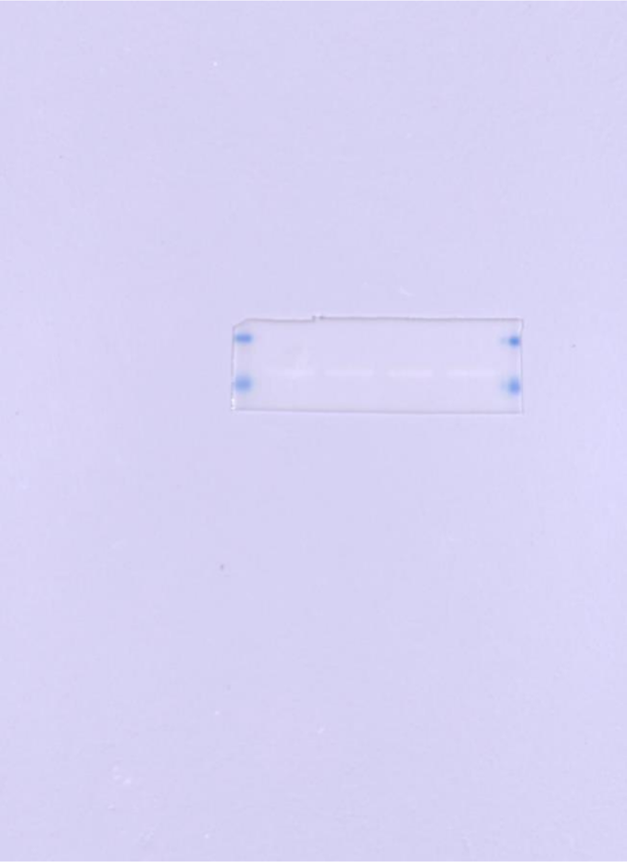

AMC-HN-8

Fig. 3E

TU212

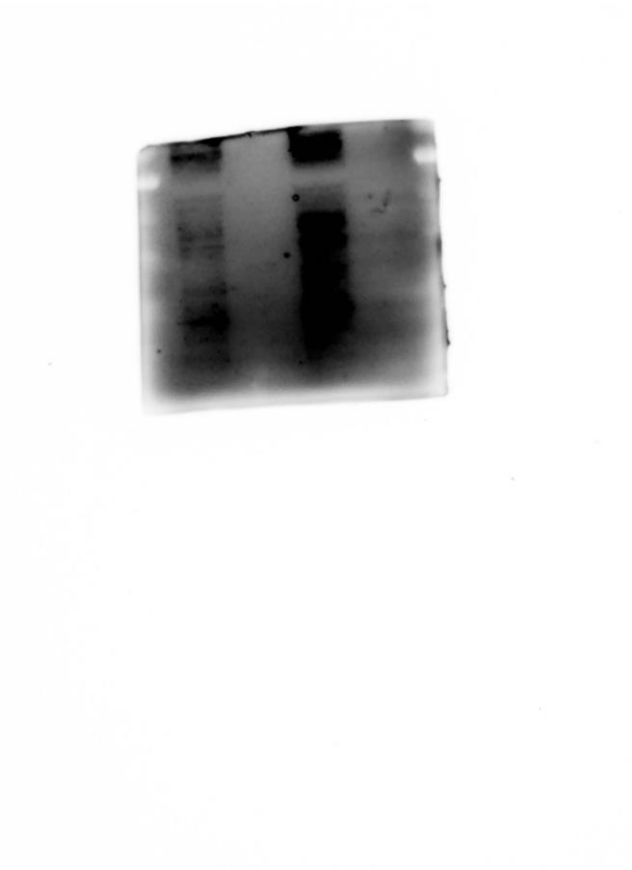

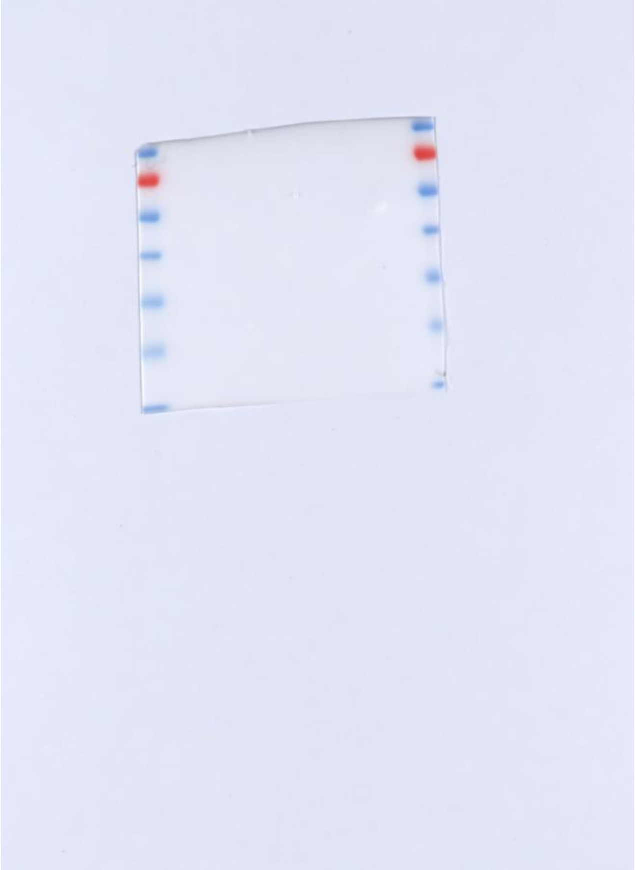

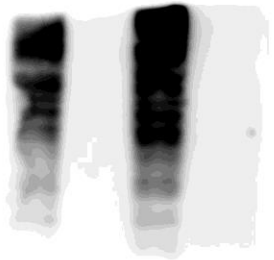

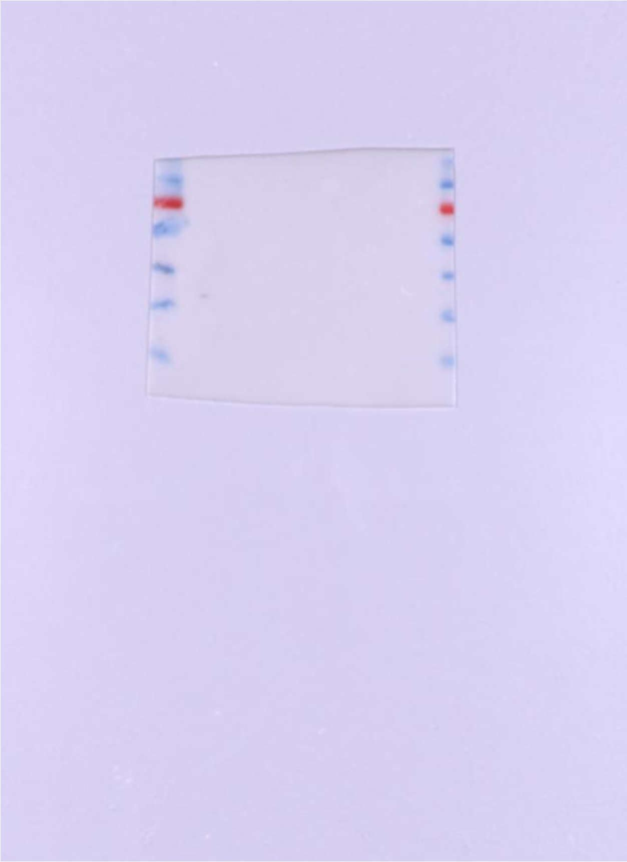

Fig. 3F

AMC-HN-8 **VHL** **TU212**

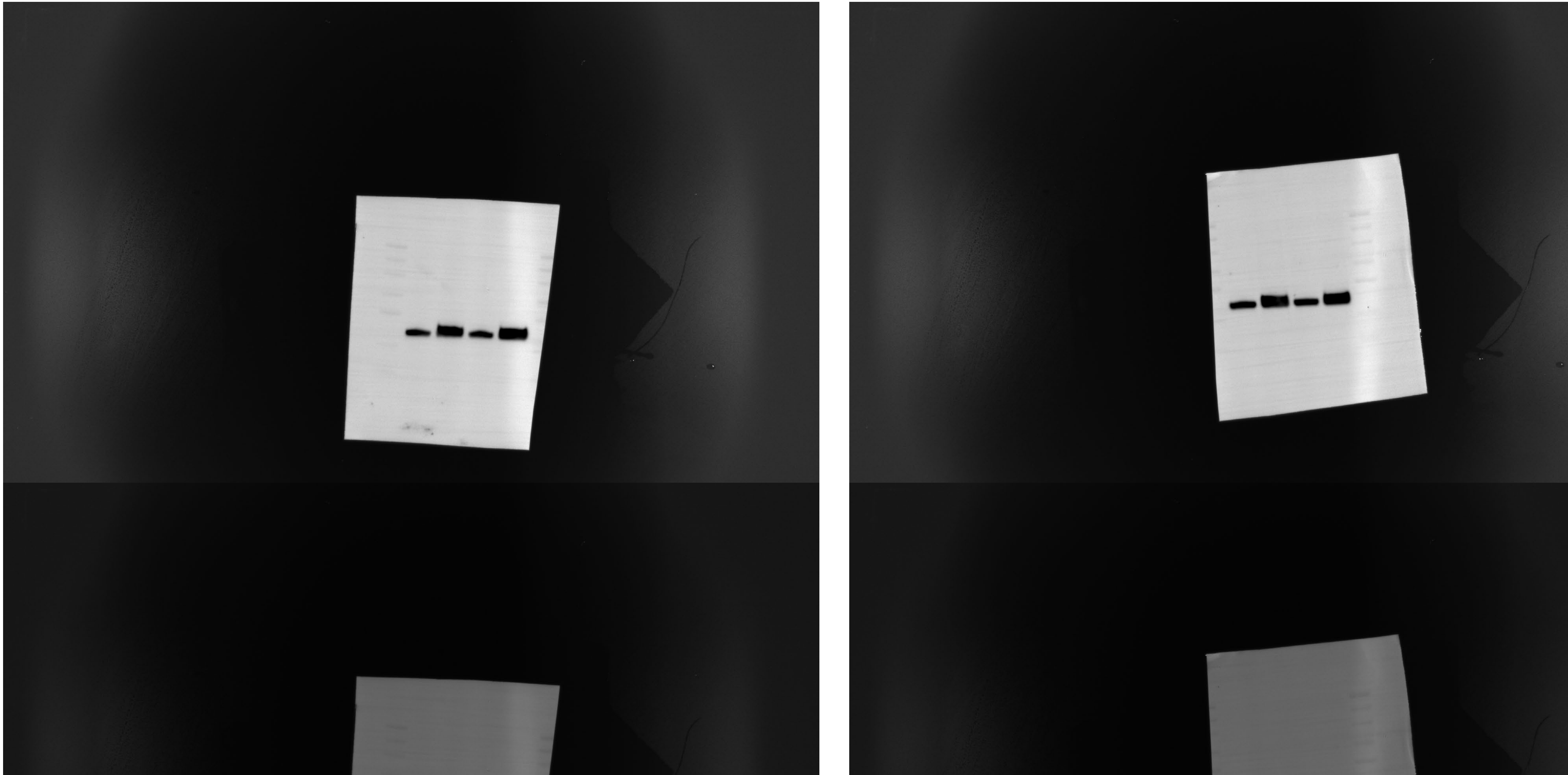

Fig. 3F

AMC-HN-8

MAP2K2

TU212

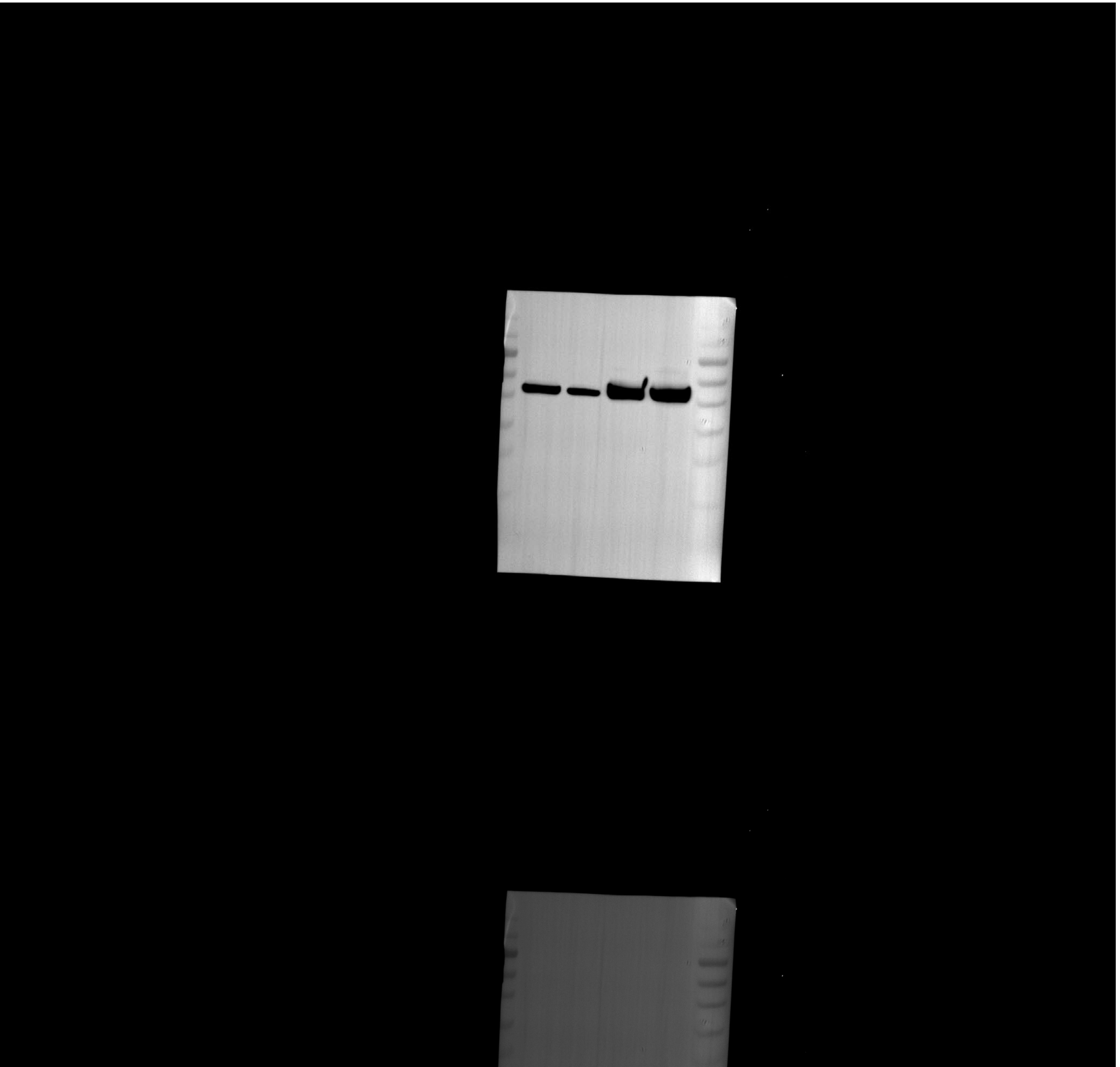

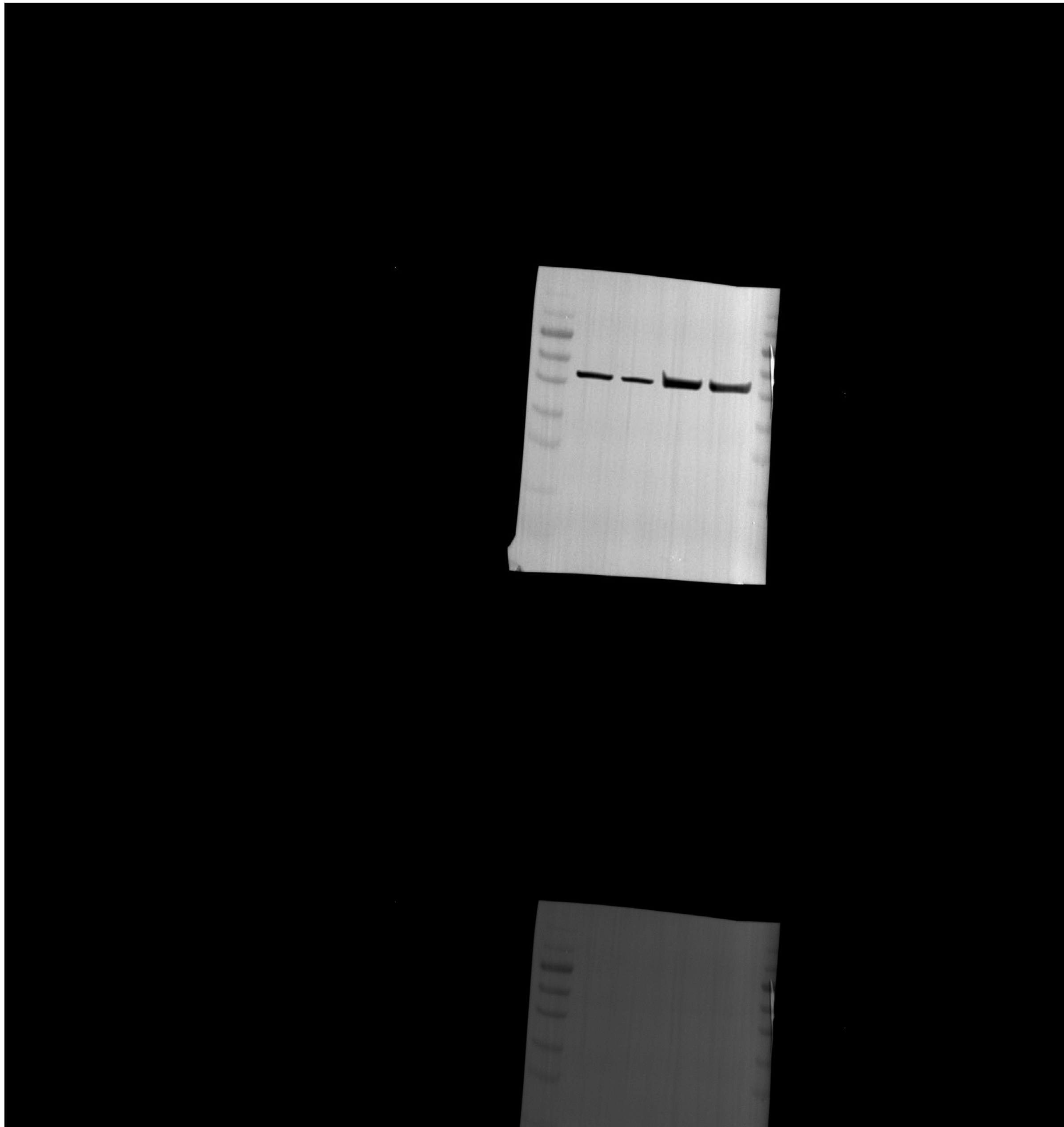

Fig. 3F

AMC-HN-8 GAPDH TU212

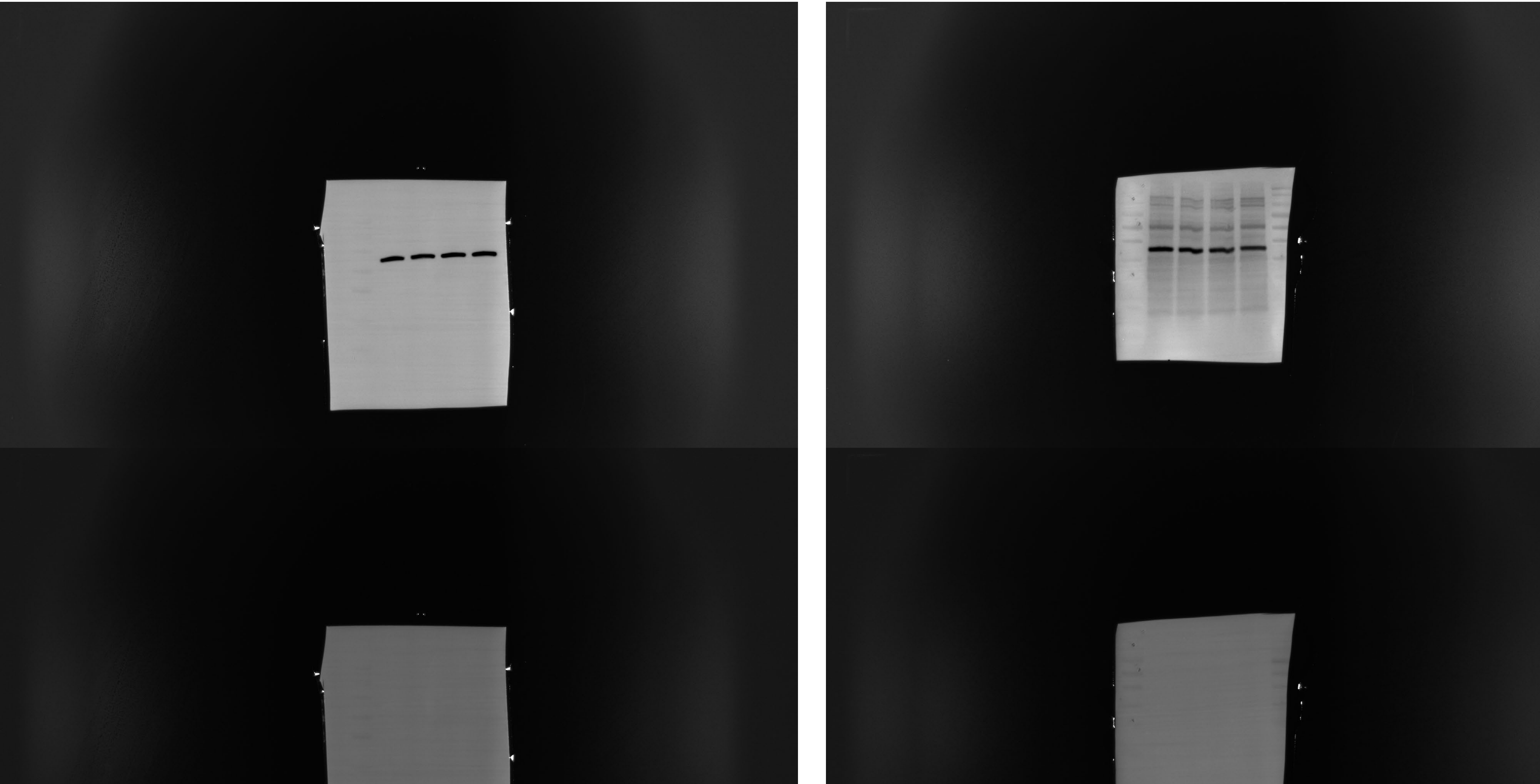

Fig. 3G

AMC-HN-8

IP MAP2K2

MAP2K2

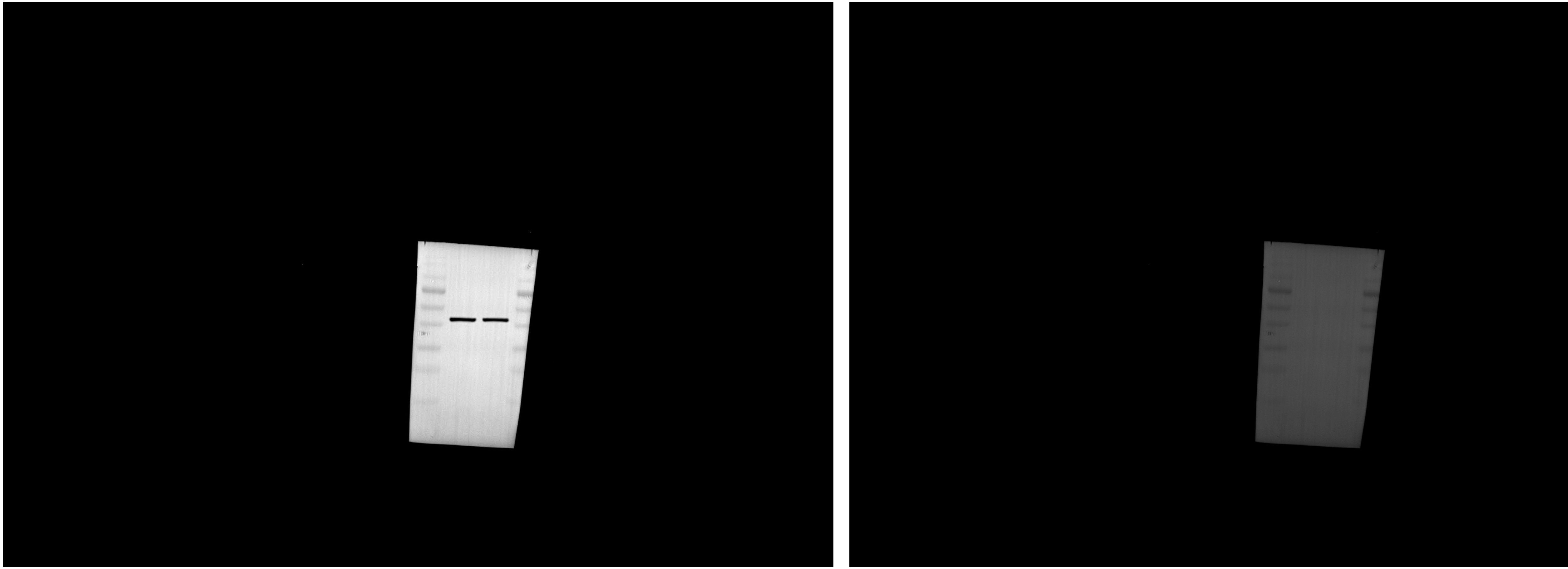

Fig. 3G

AMC-HN-8

IP MAP2K2

Ubiquitin

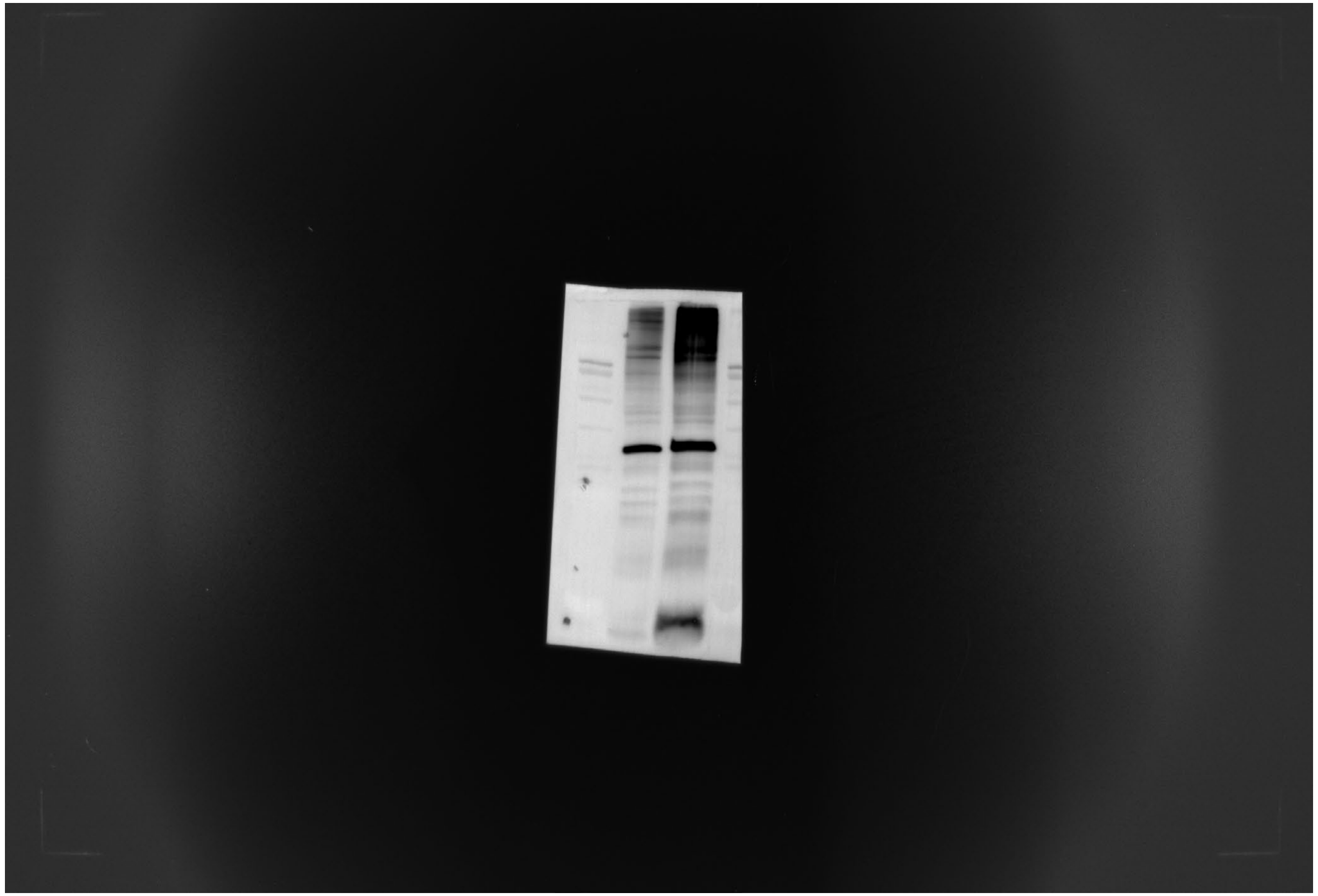

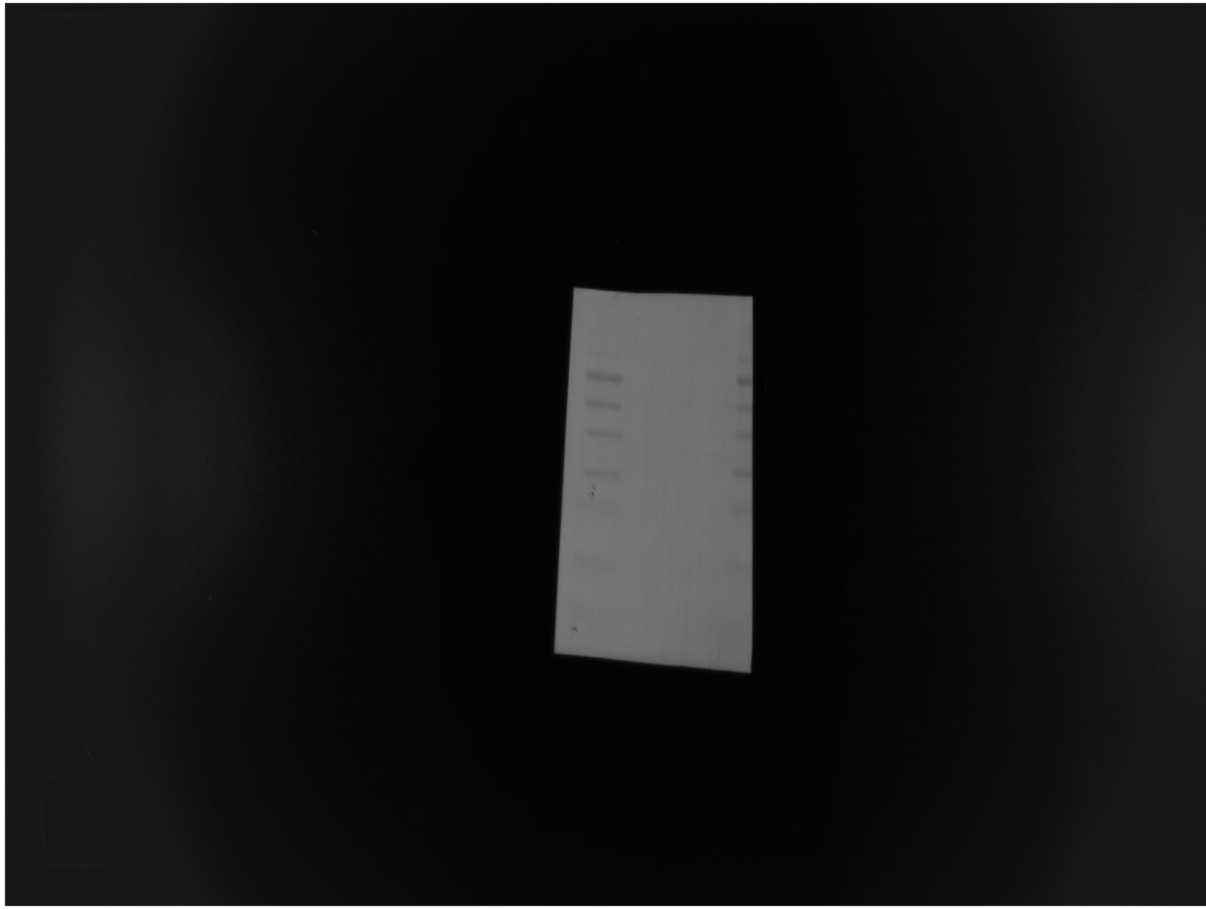

Fig. 3G

AMC-HN-8 INPUT

GAPDH

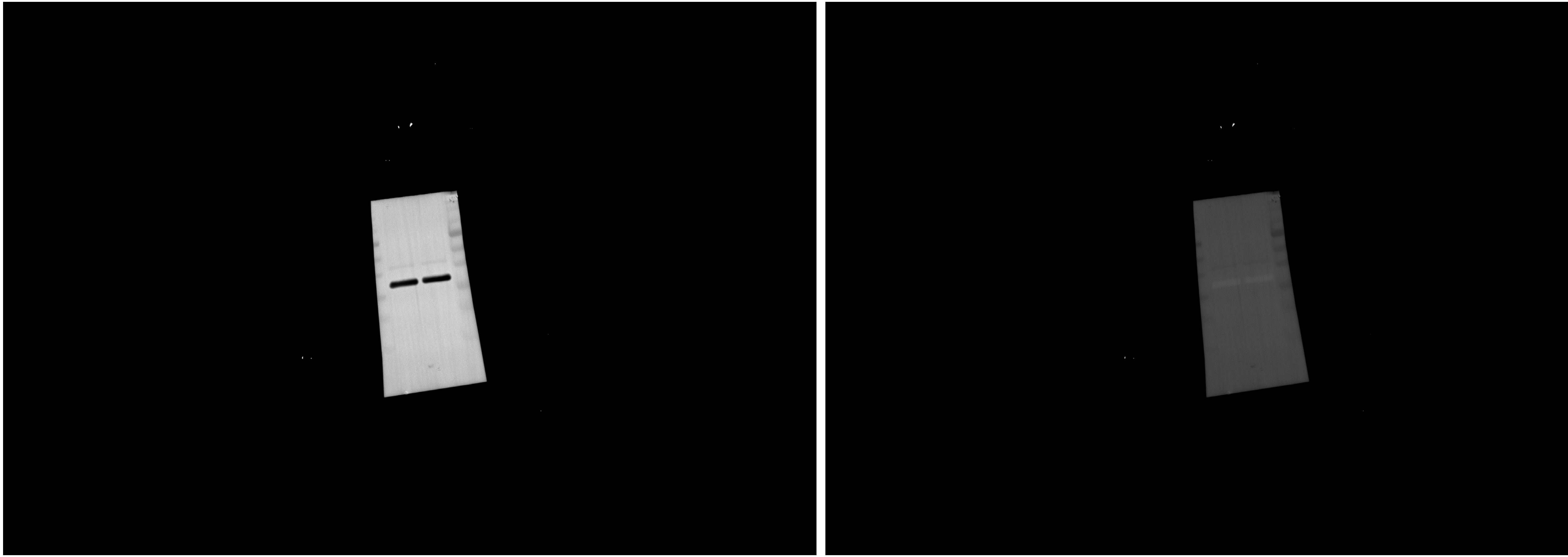

Fig. 3G

AMC-HN-8

INPUT

MAP2K2

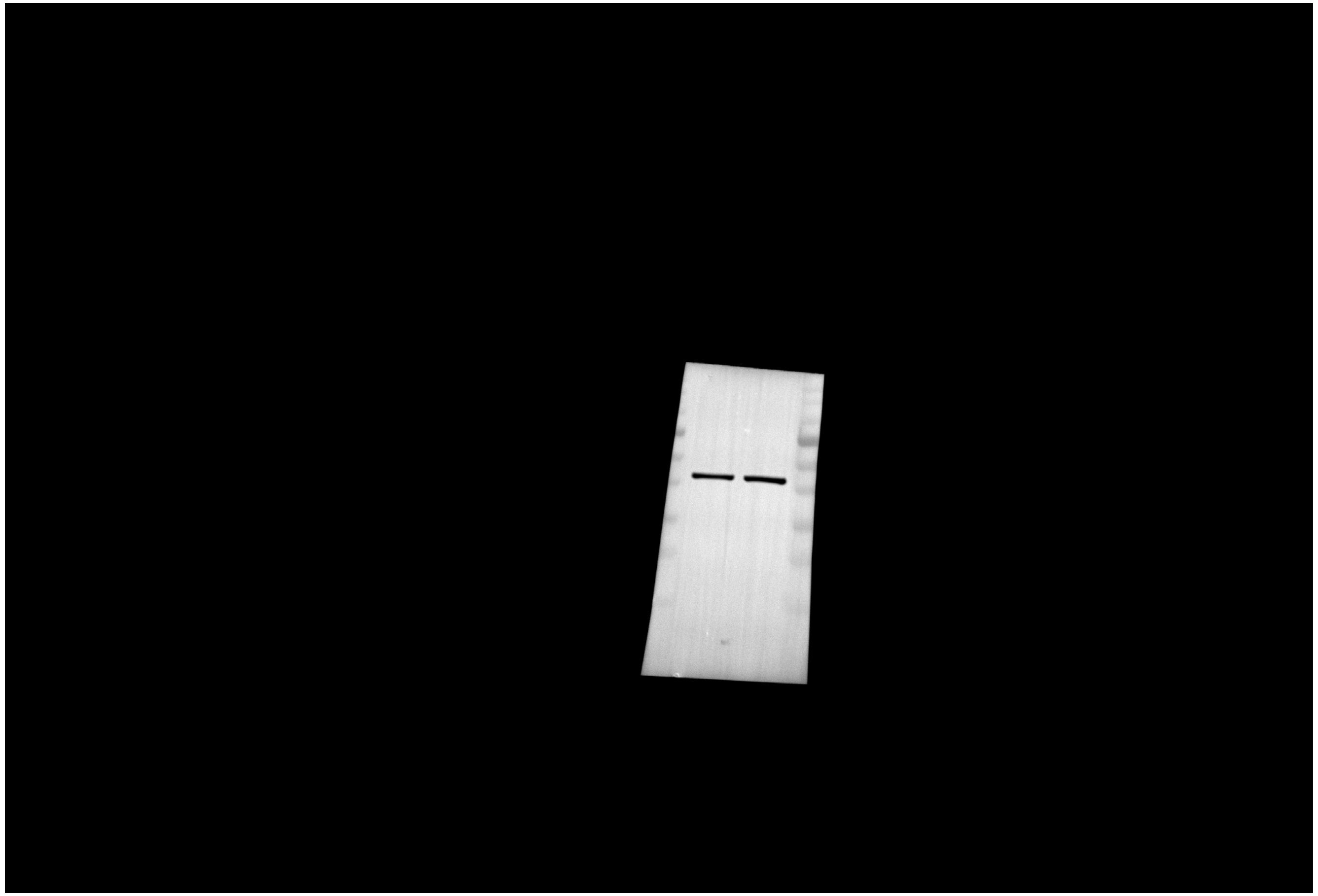

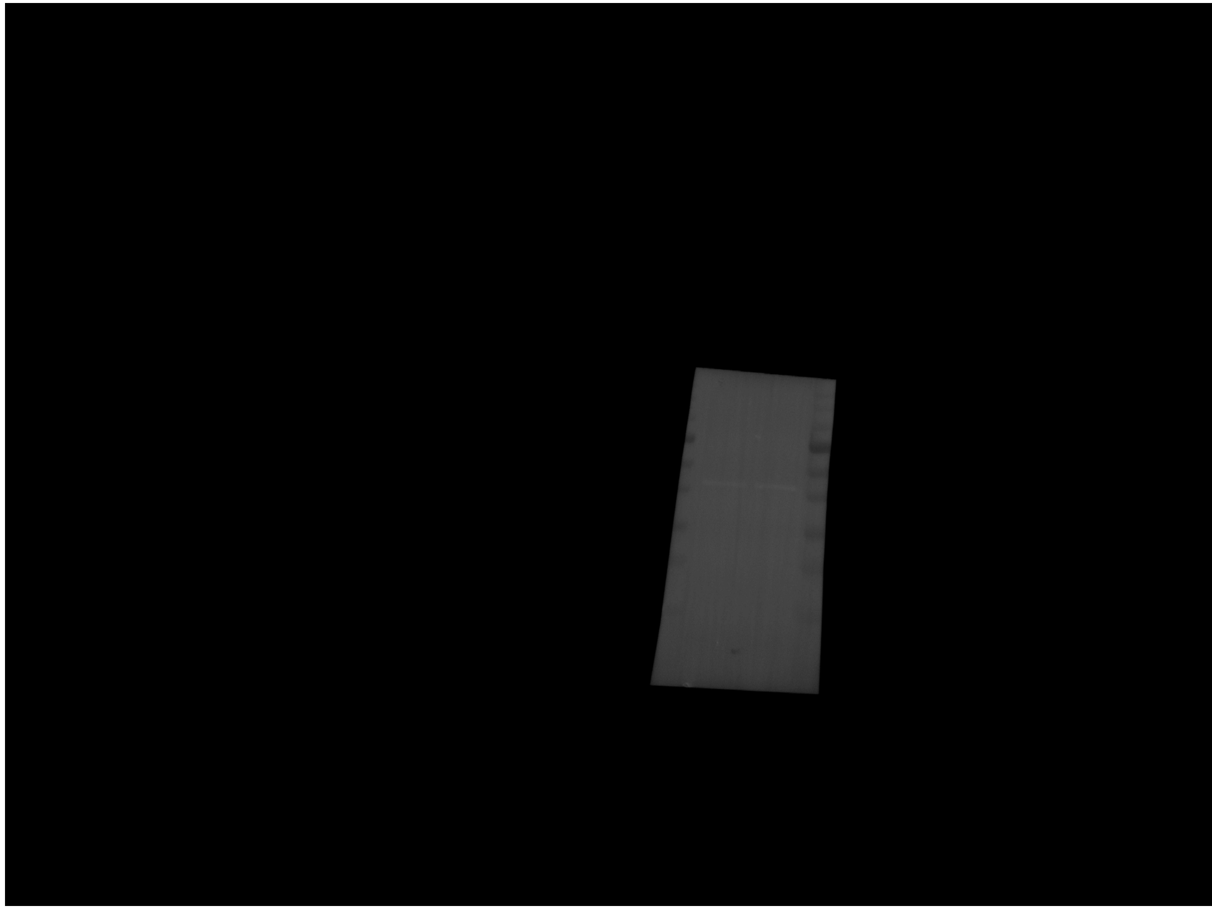

Fig. 3G

AMC-HN-8

INPUT

Ubiquitin

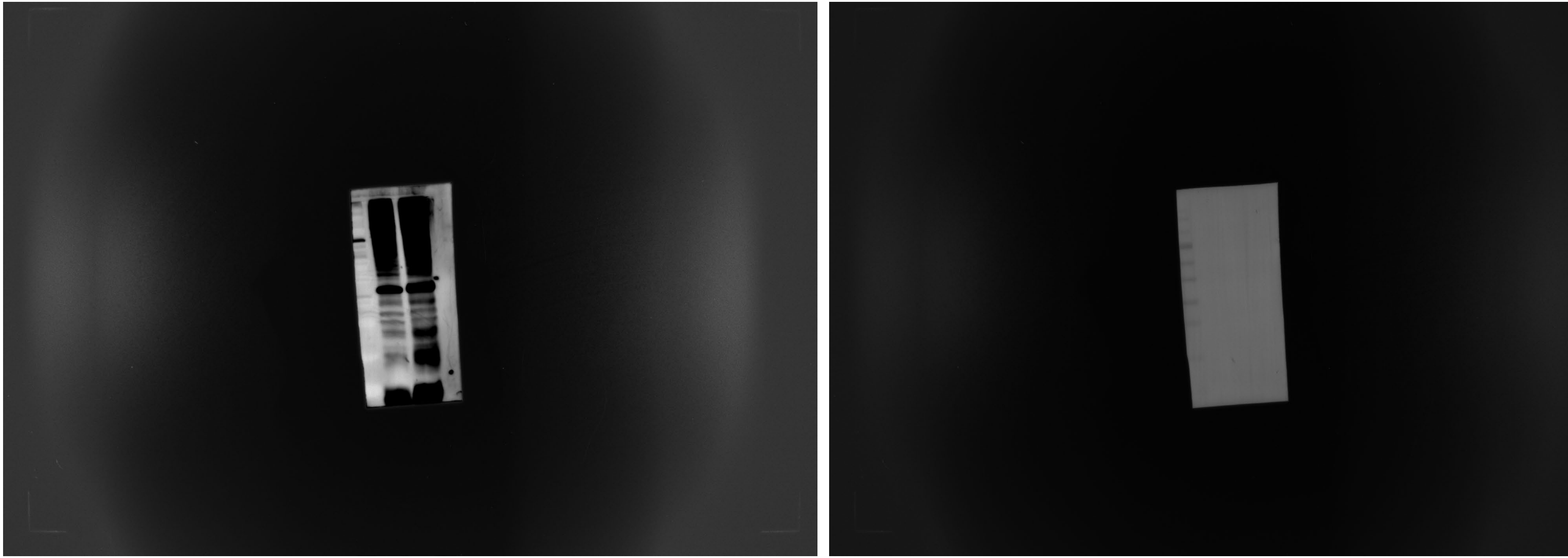

Fig. 3G

AMC-HN-8

INPUT

VHL

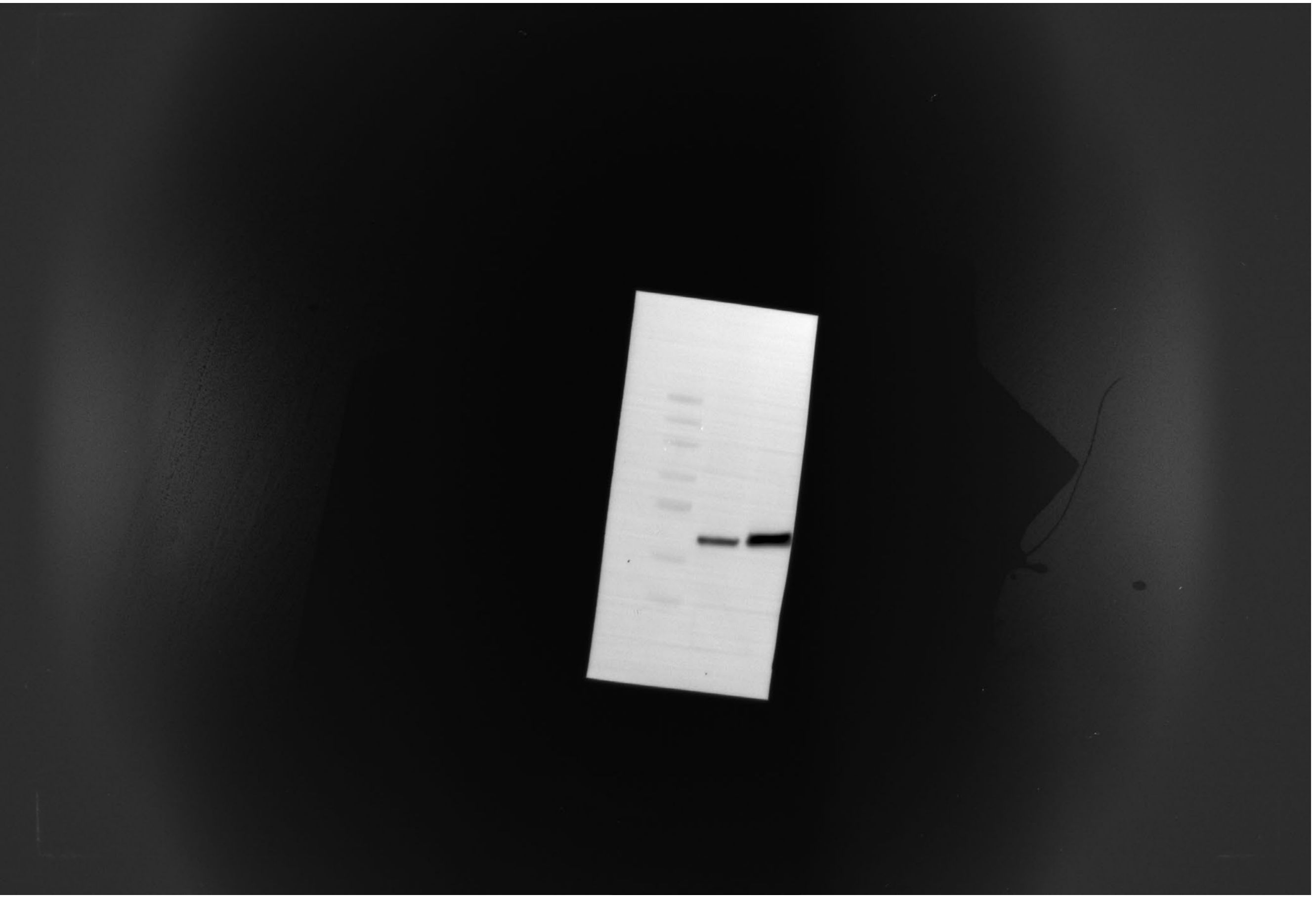

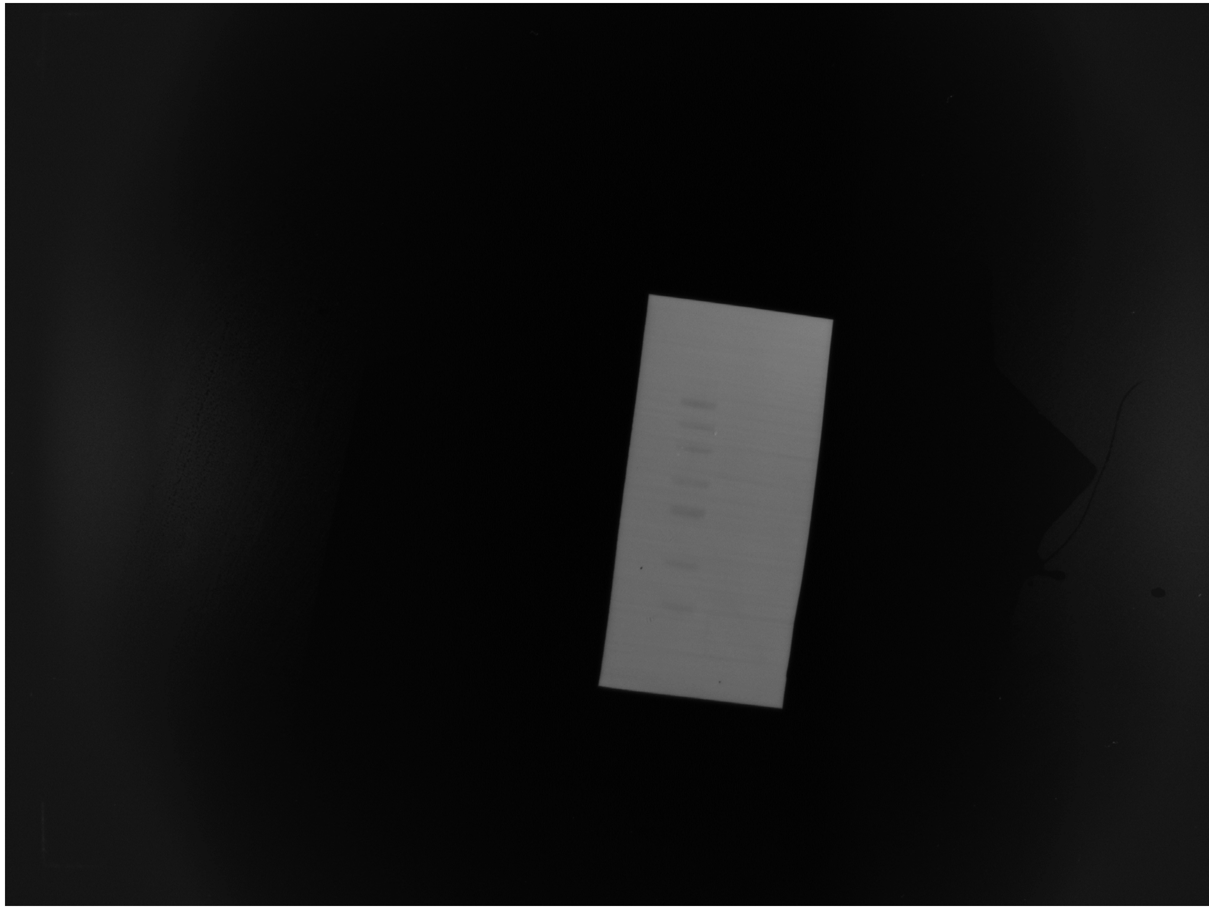

Fig. 3G

TU212

IP MAP2K2

MAP2K2

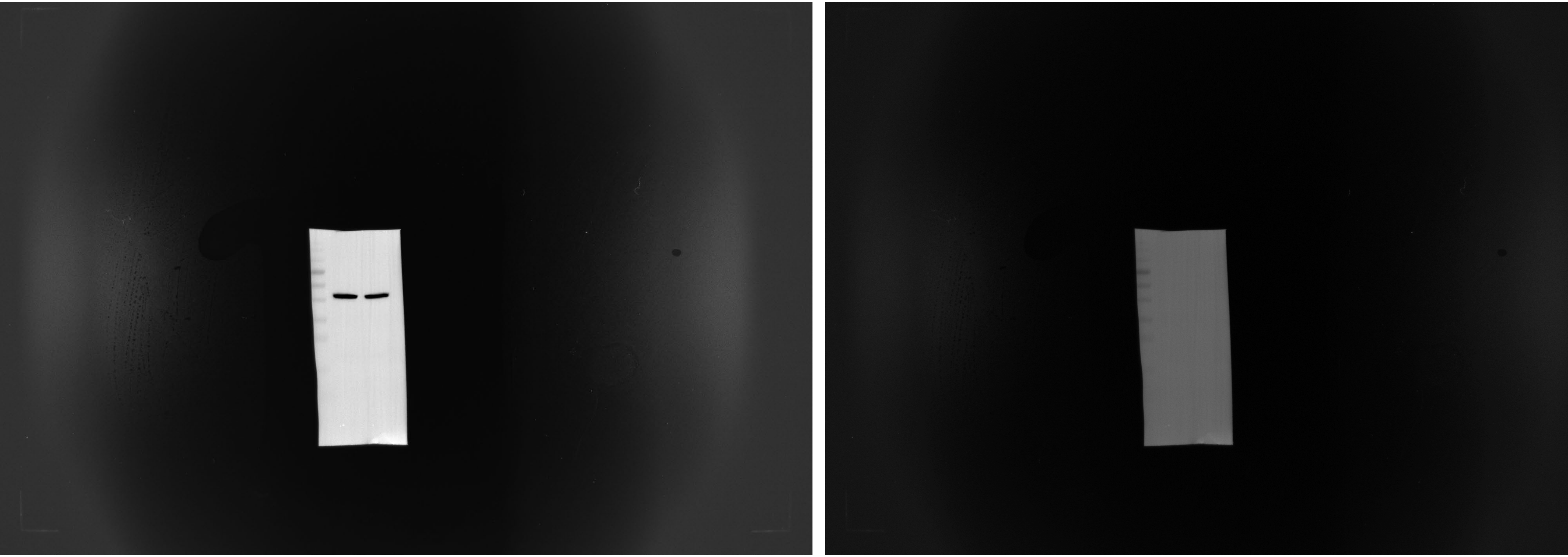

Fig. 3G

TU212

IP MAP2K2

Ubiquitin

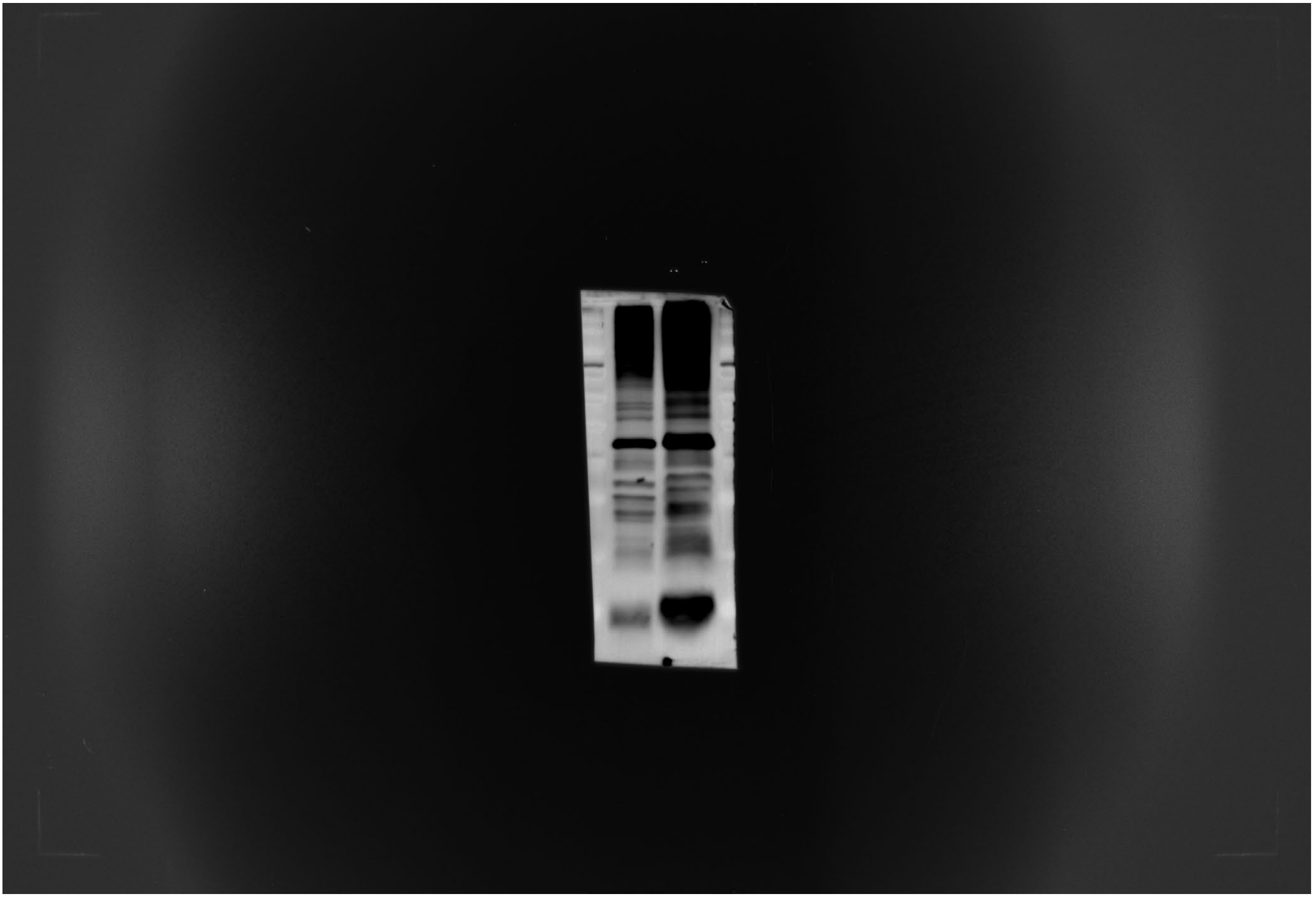

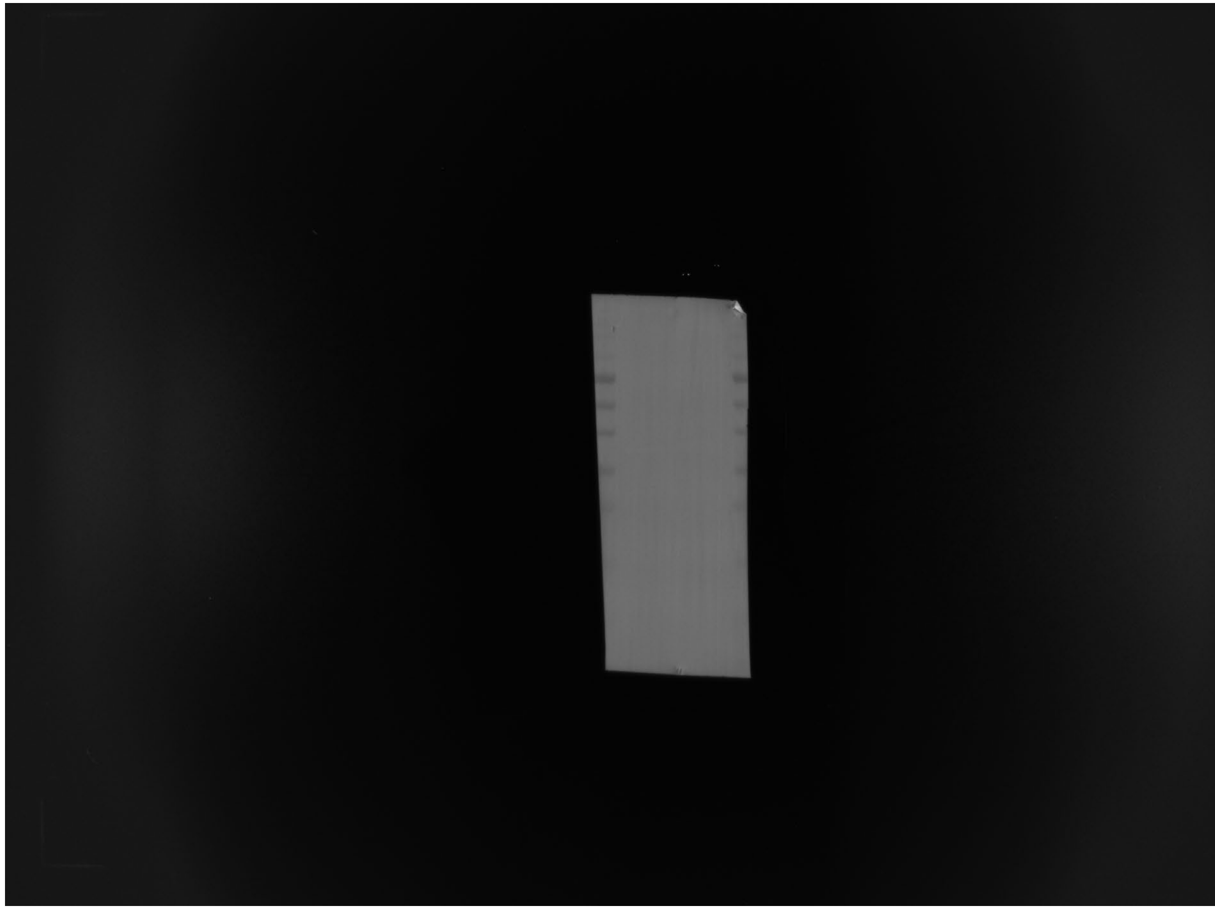

Fig. 3G

TU212

INPUT

GAPDH

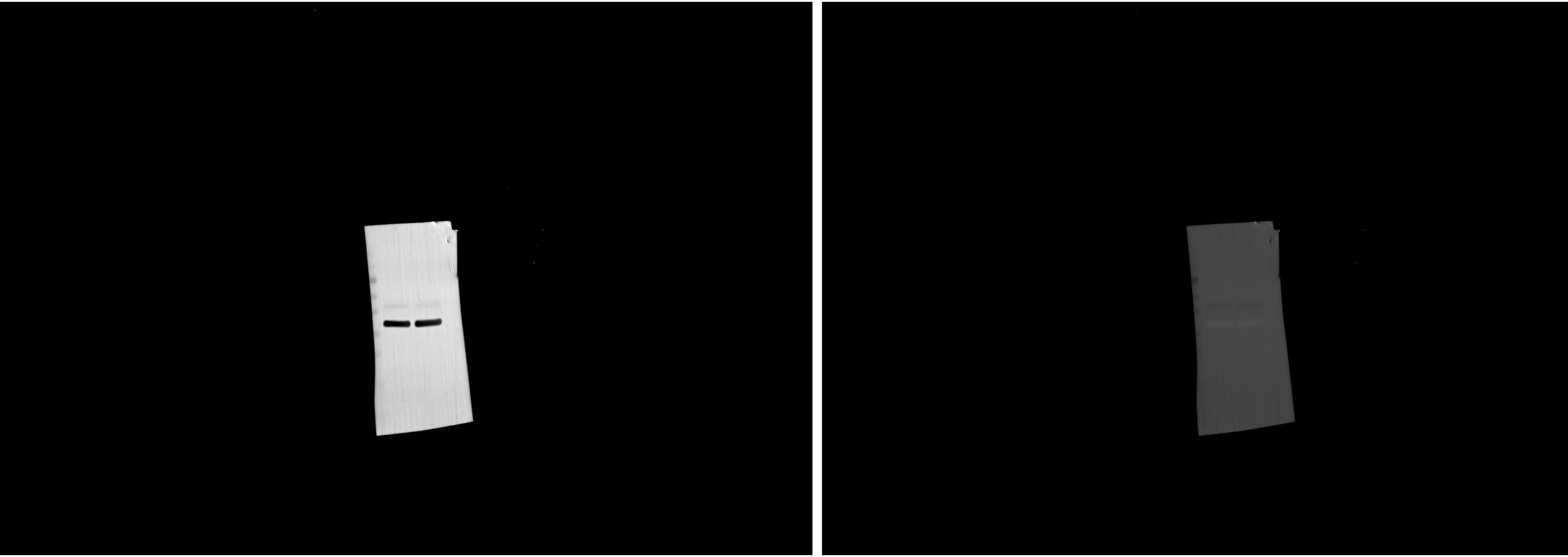

Fig. 3G

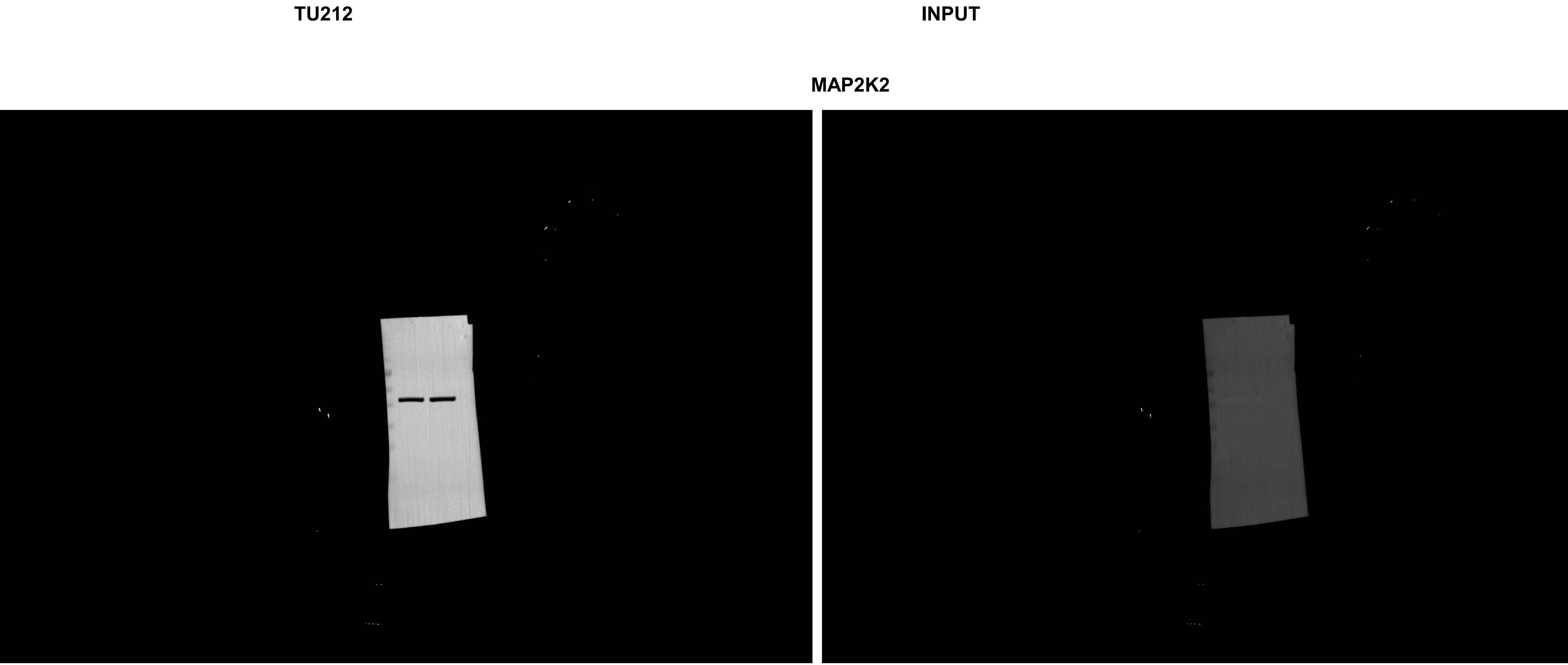

Fig. 3G

TU212

INPUT

Ubiquitin

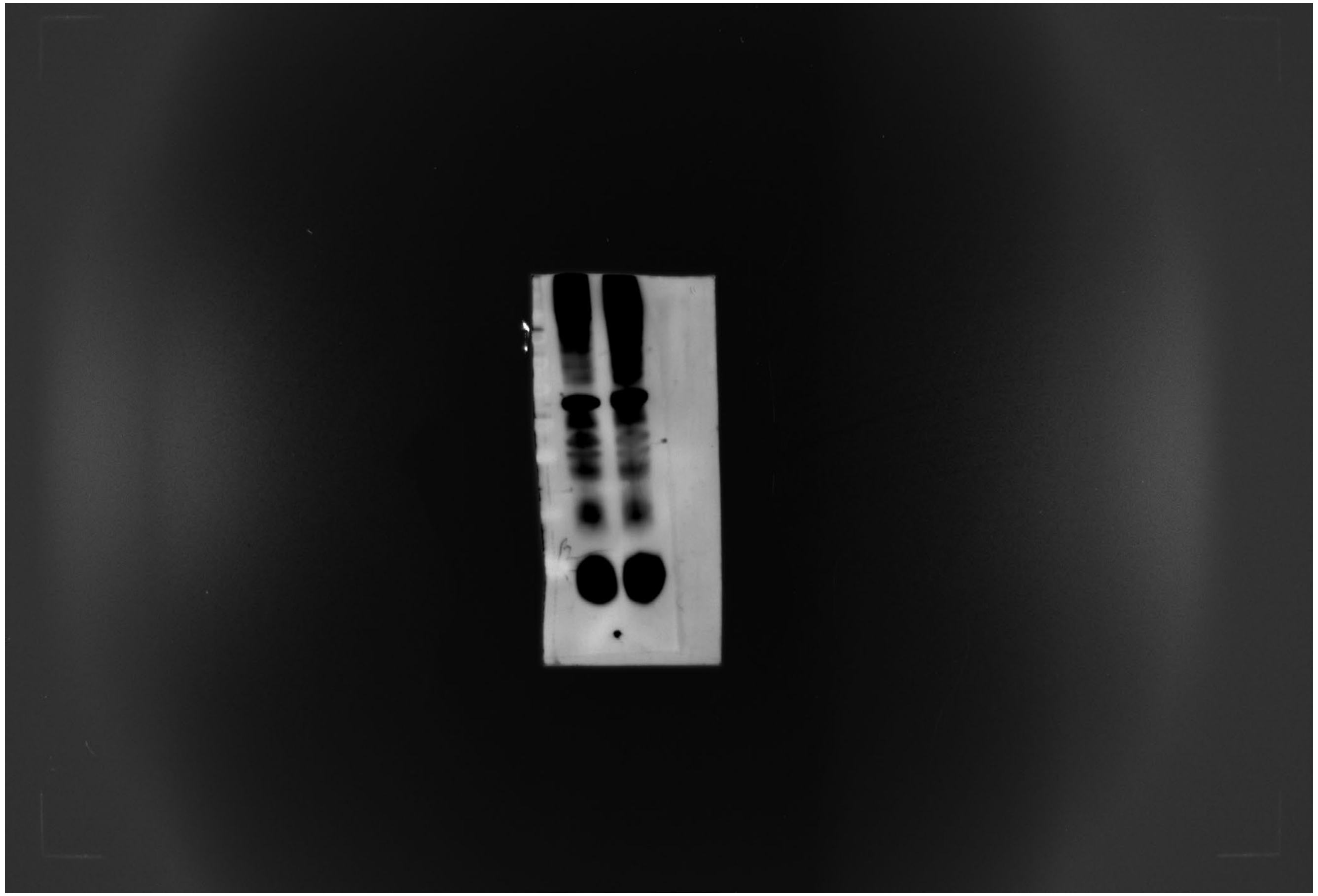

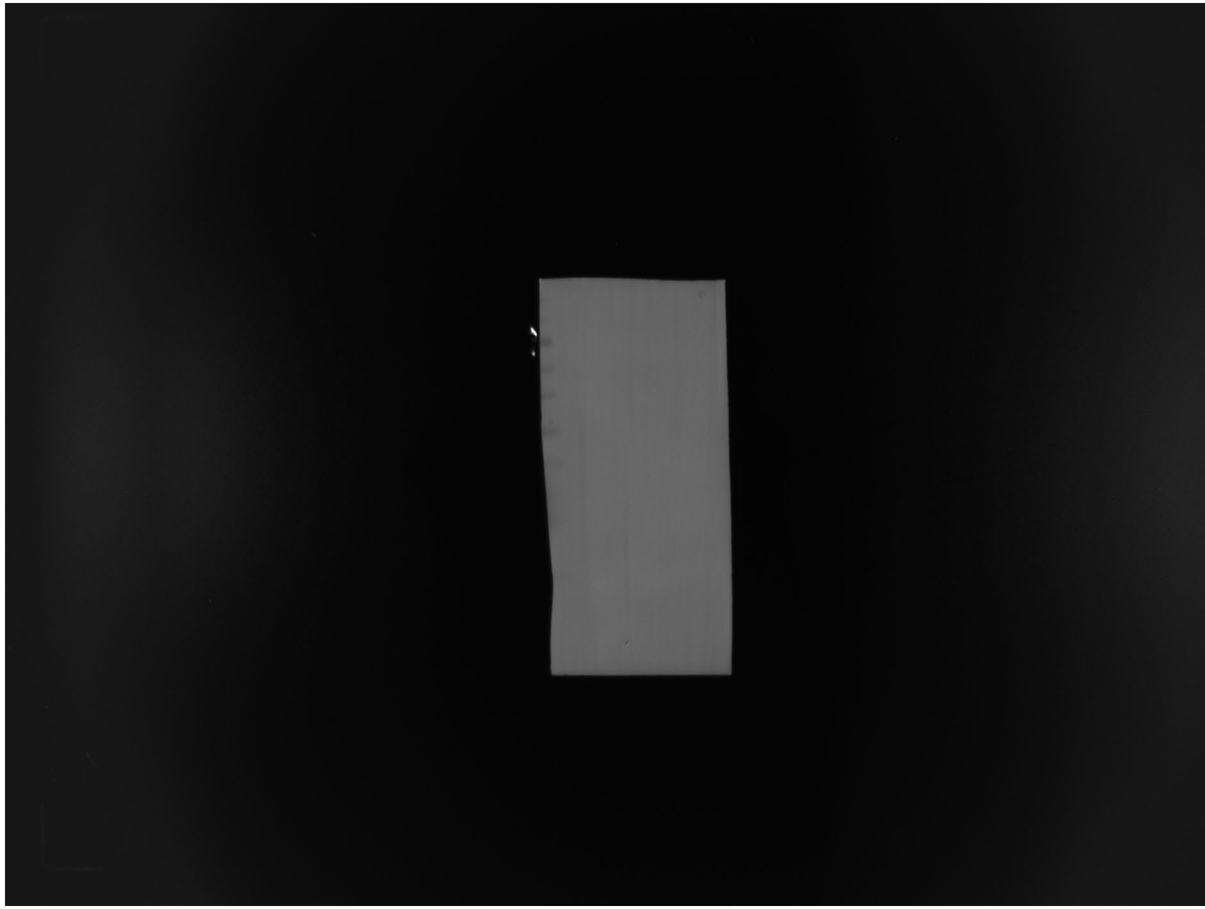

Fig. 3G

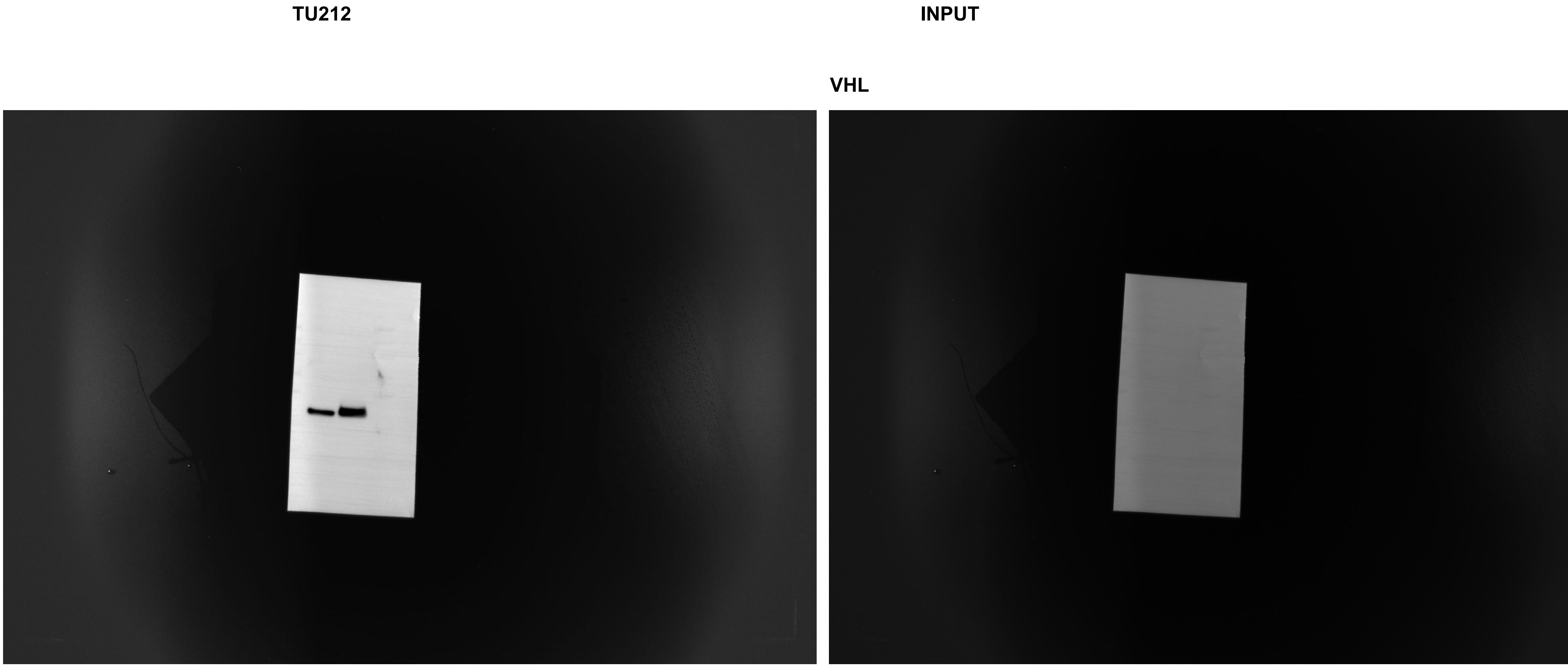

Fig. 3H

IP MAP2K2

Ubiquitin

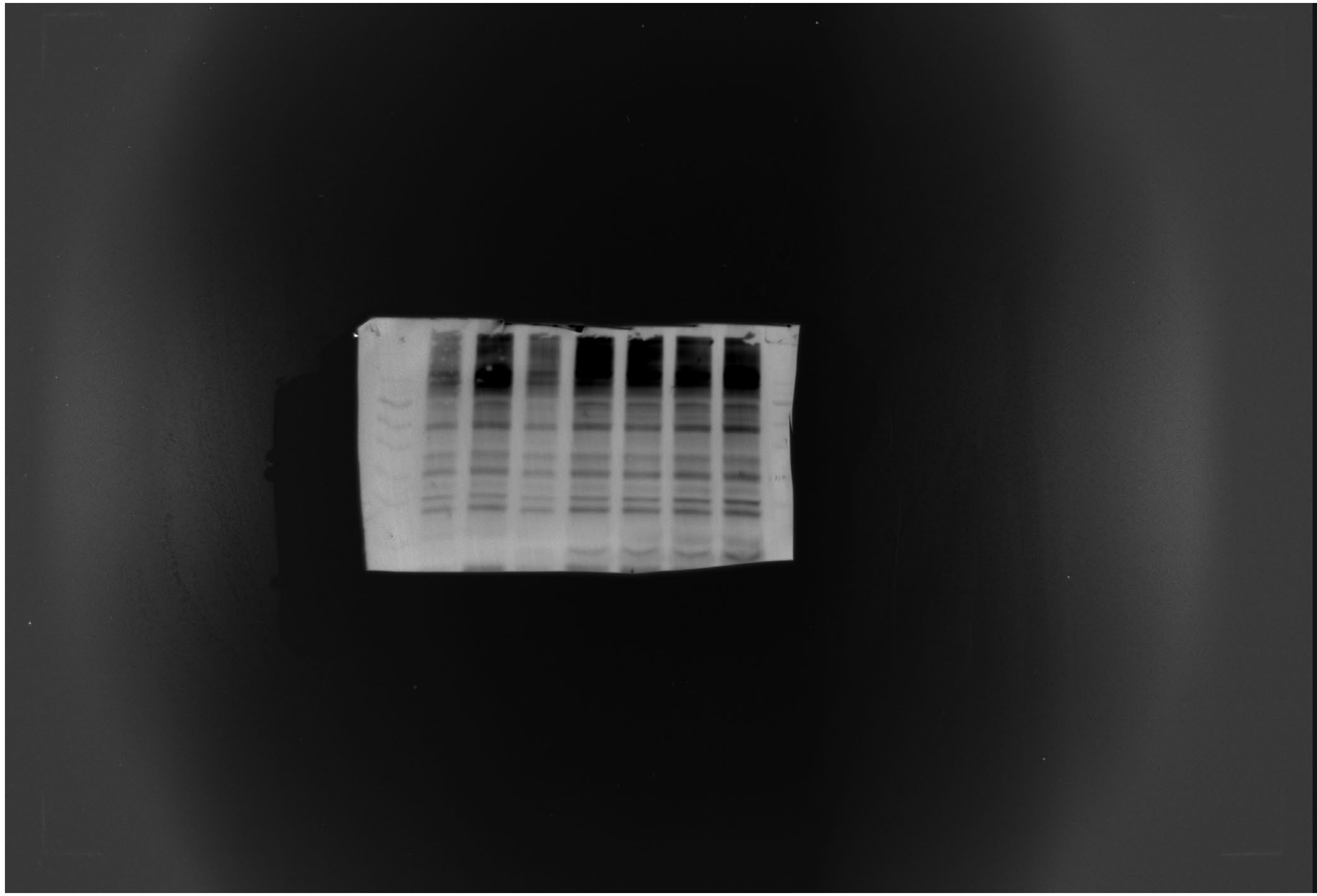

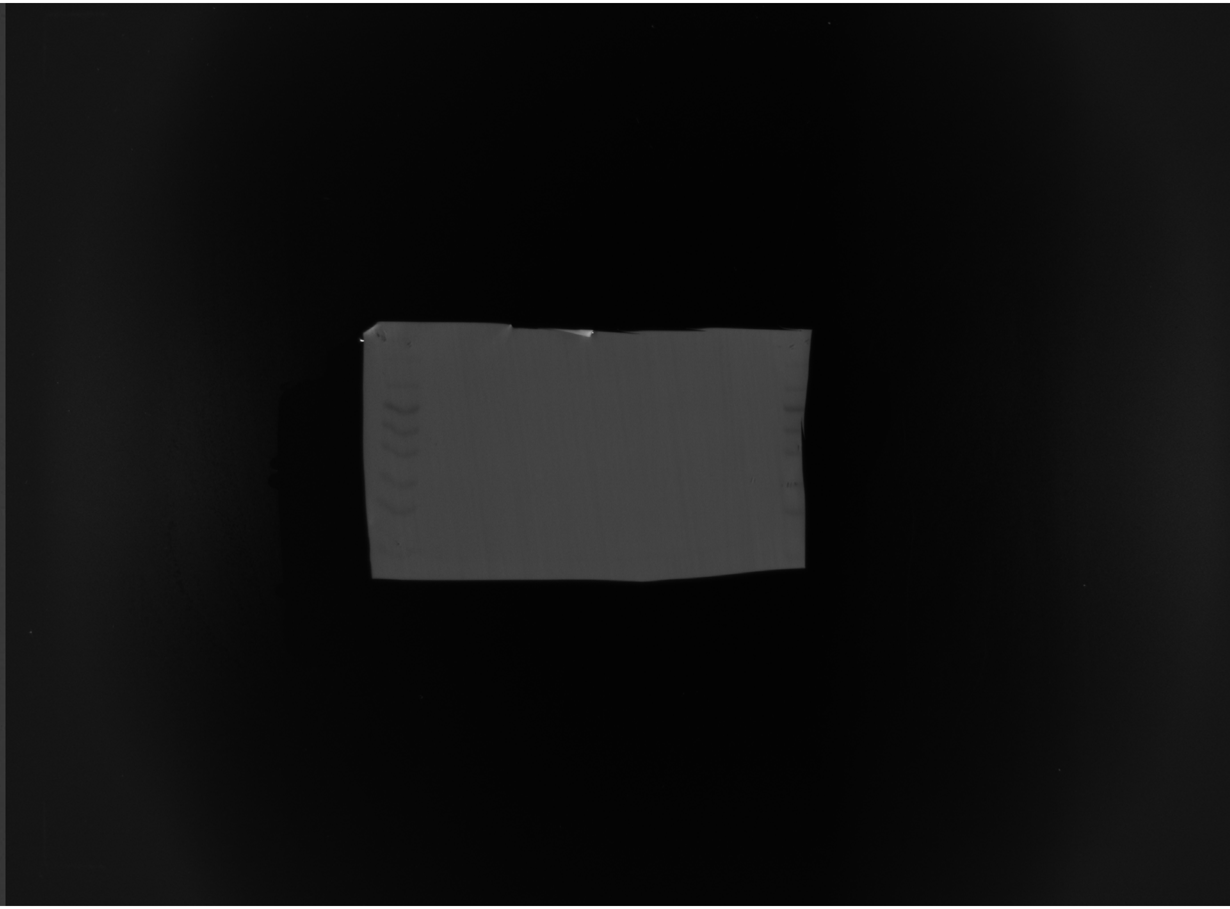

Fig. 3H

IP MAP2K2 **MAP2K2**

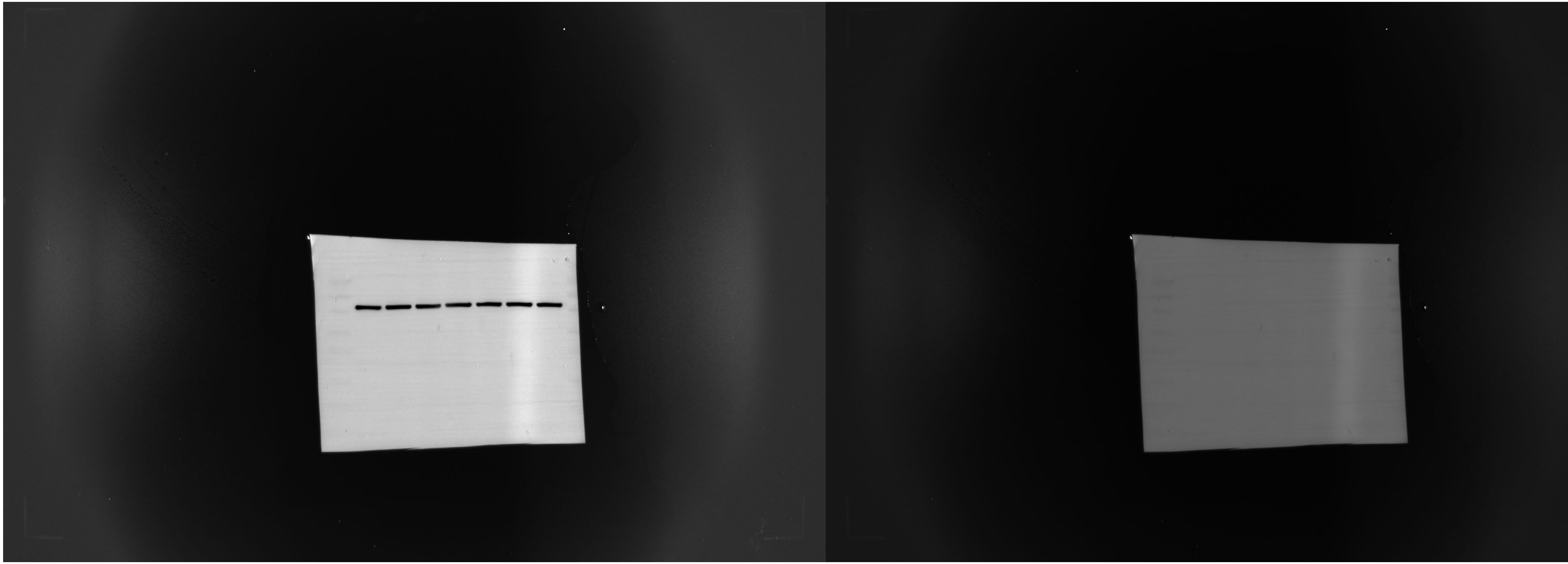

Fig. 3H

INPUT GAPDH

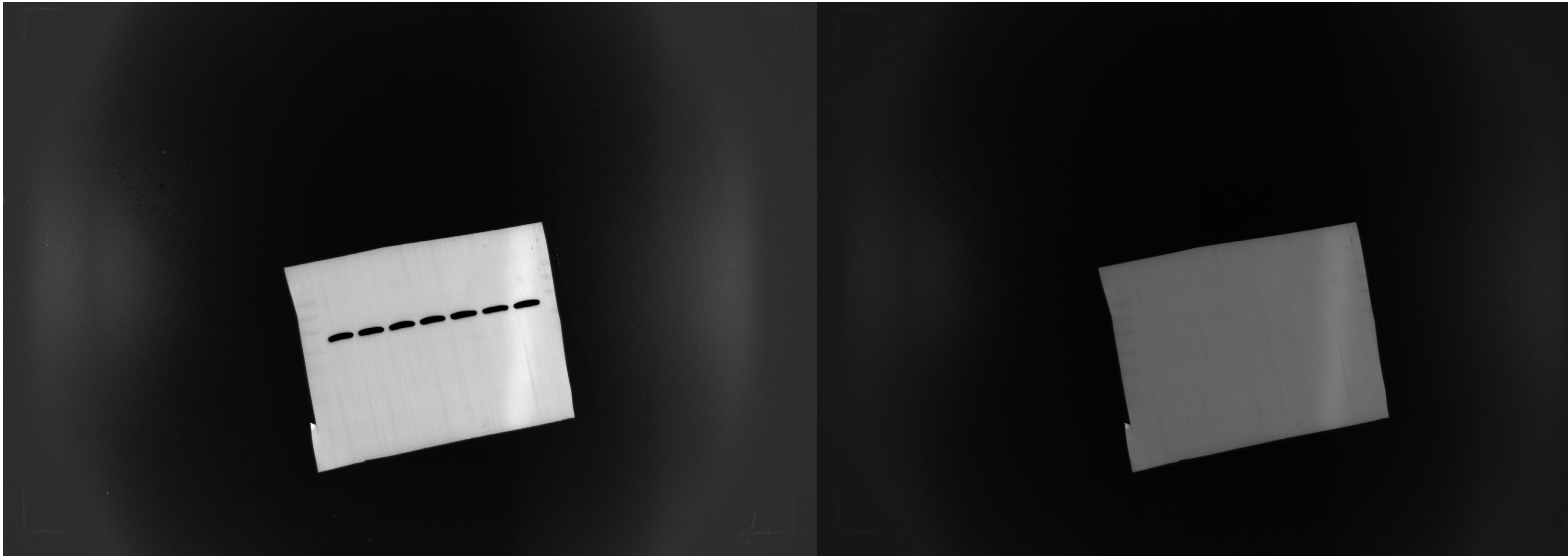

Fig. 3H

INPUT MAP2K2

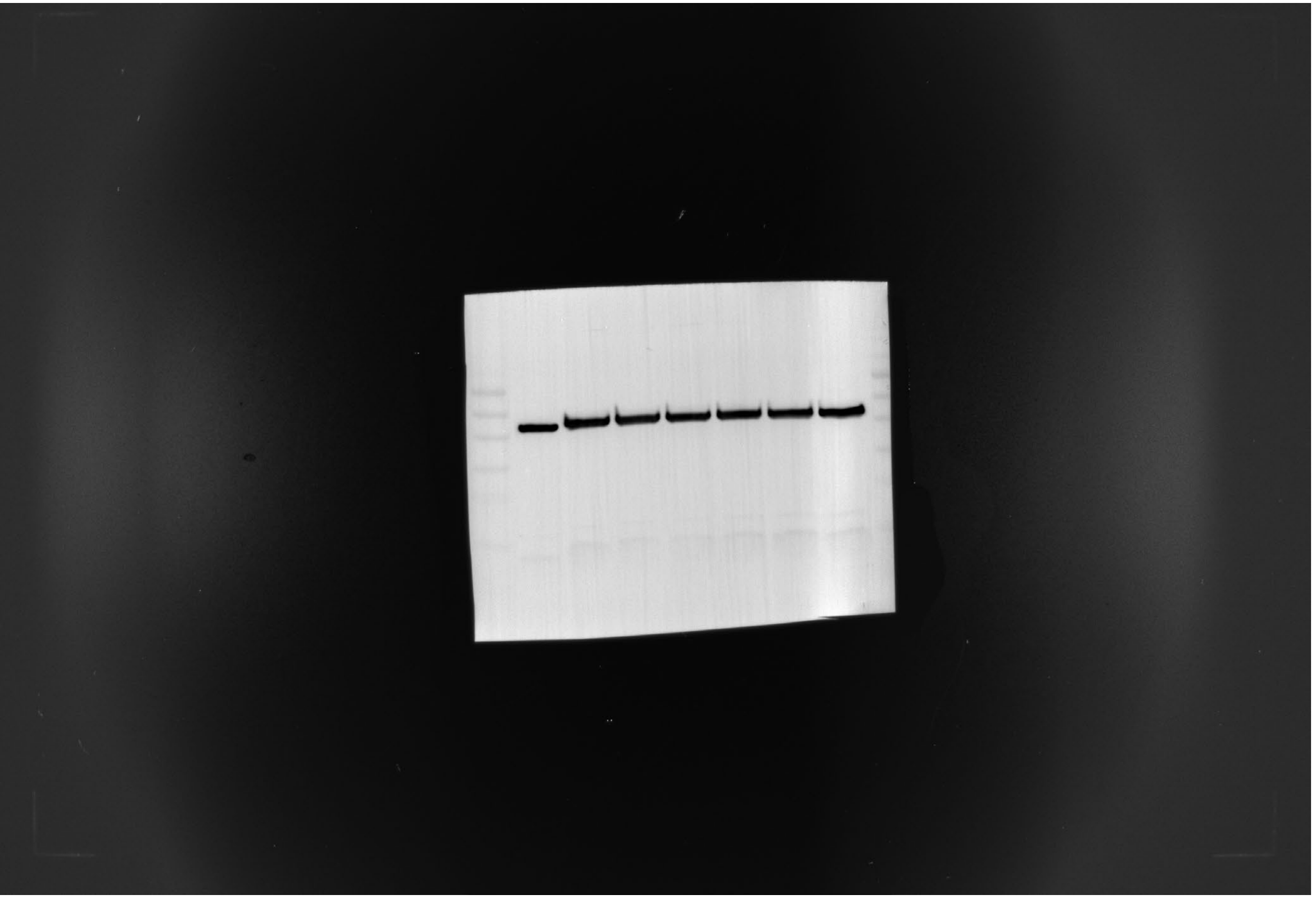

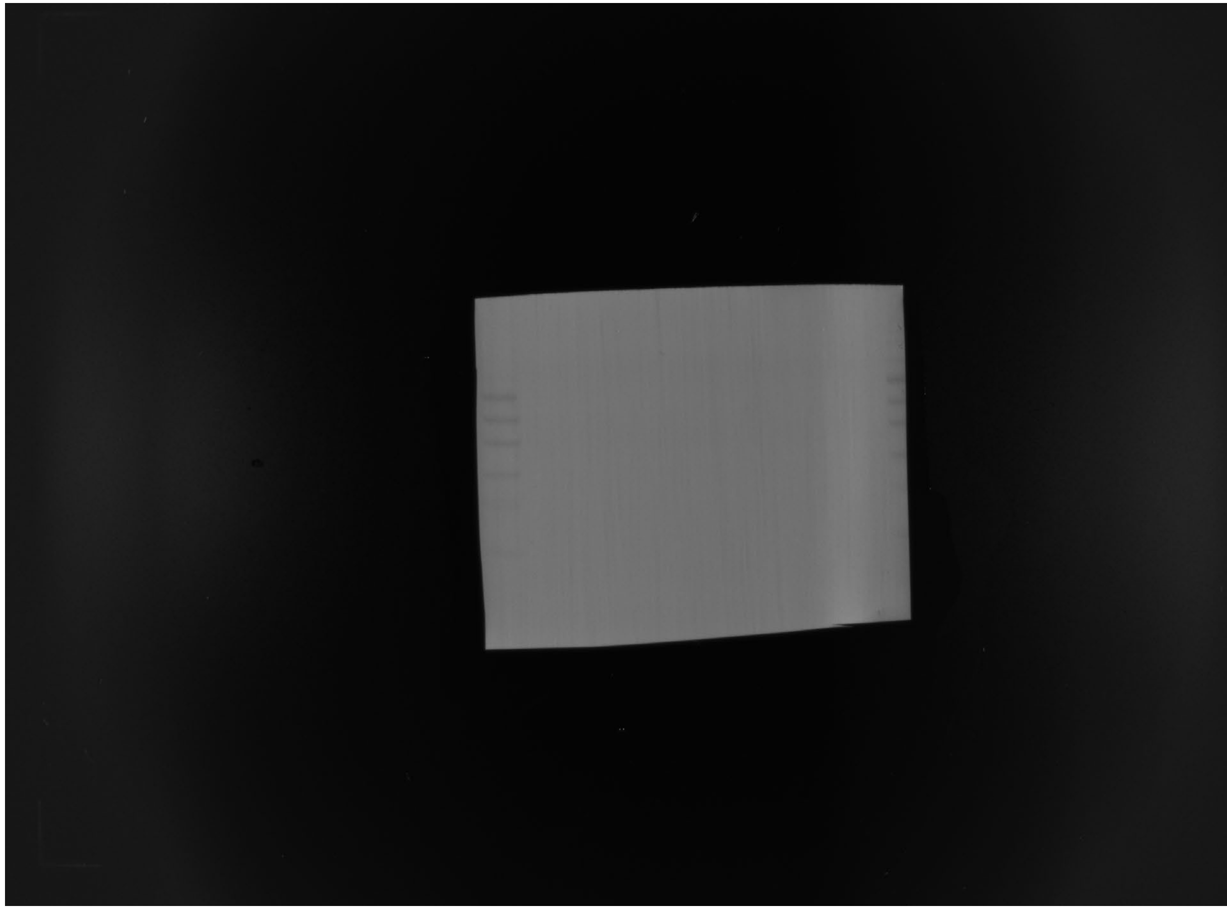

Fig. 3H

INPUT Ubiquitin

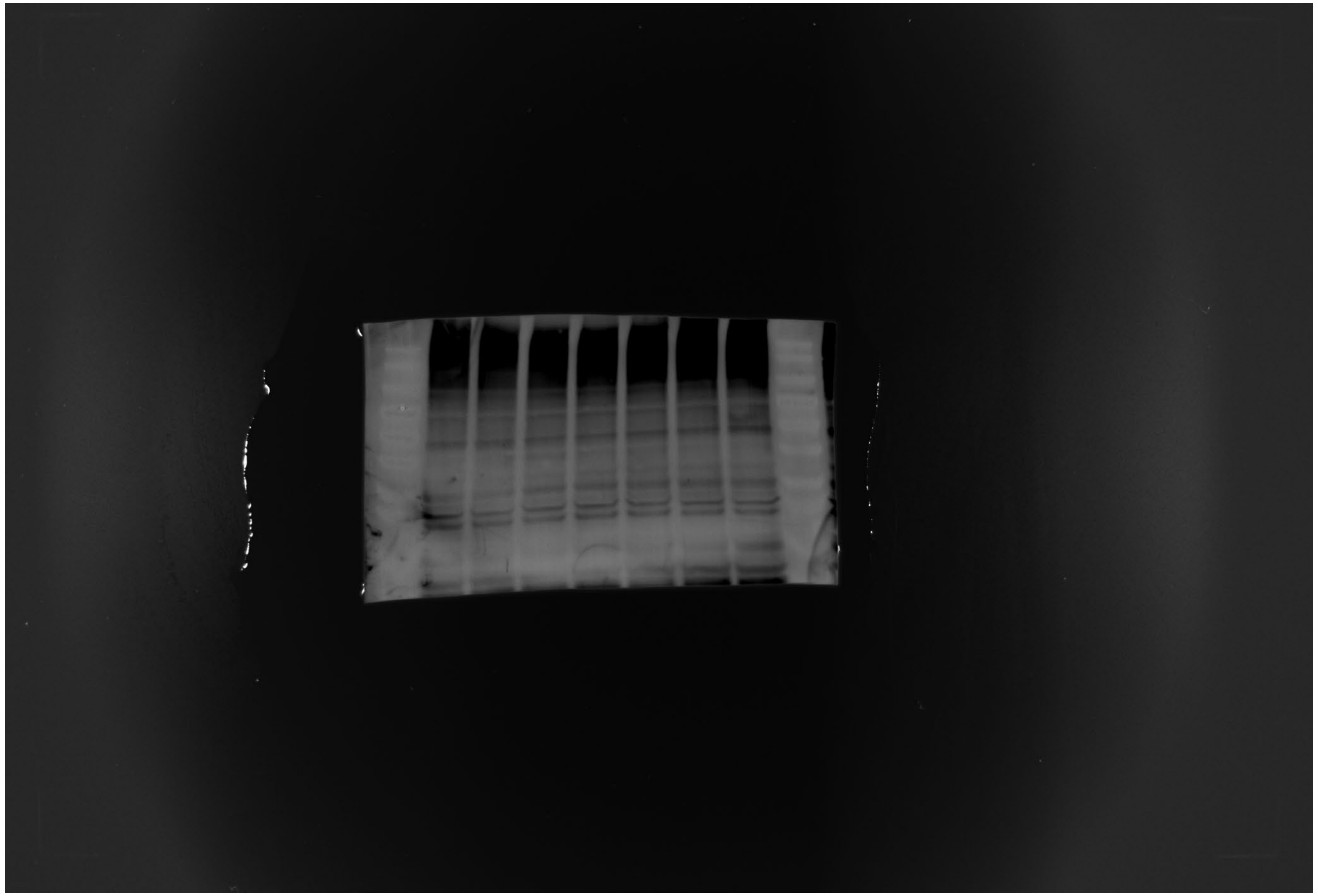

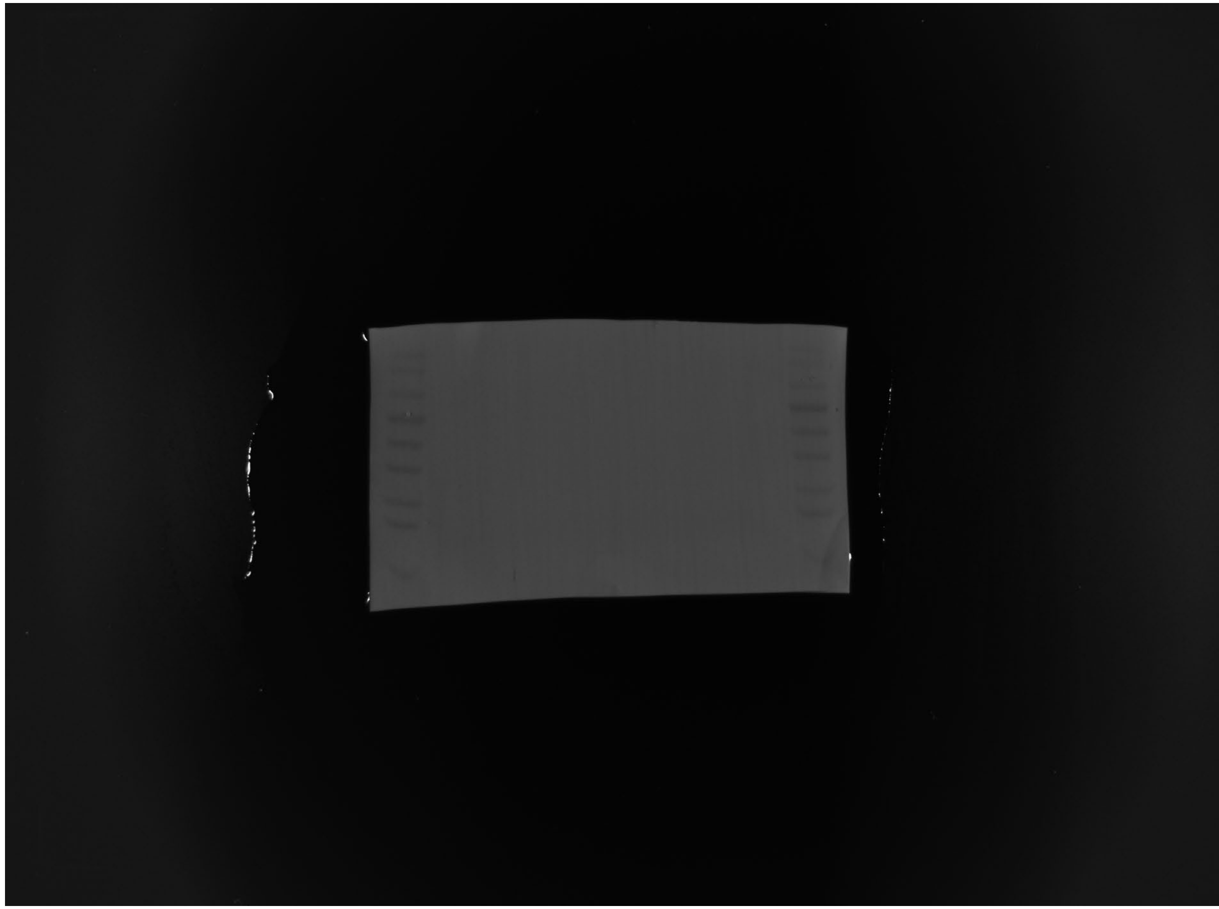

Fig. 3H

INPUT VHL

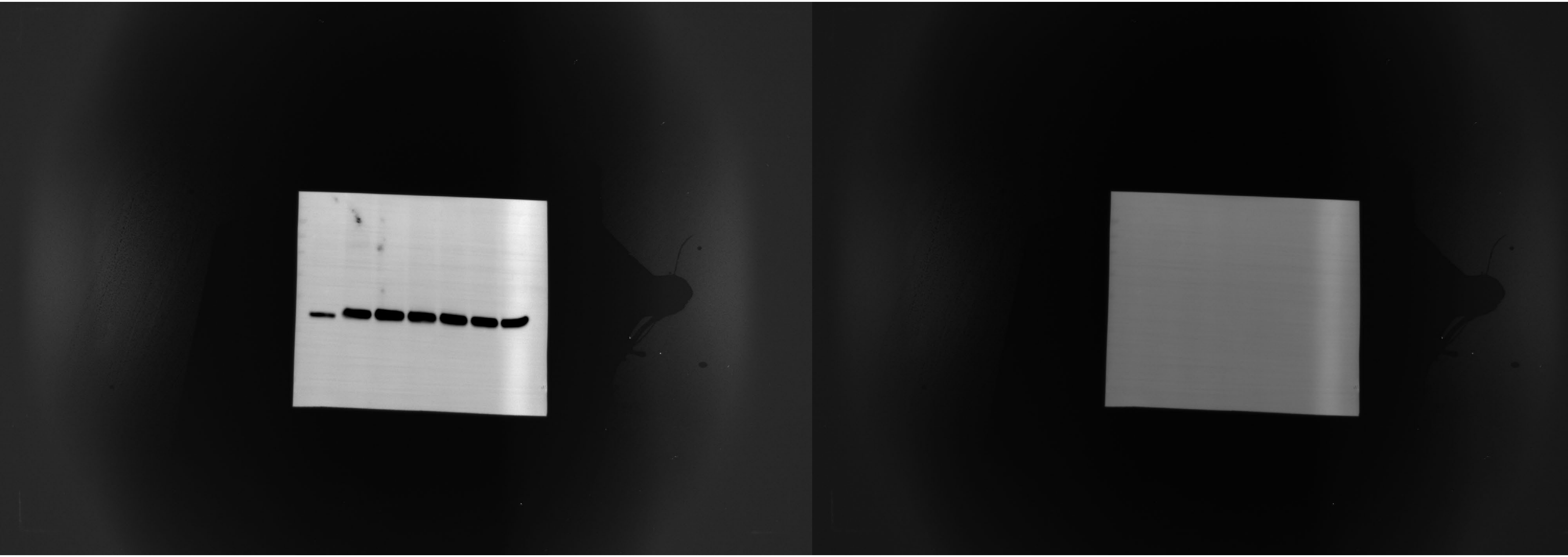

Fig. 3I

AMC-HN-8 **TU212**

MAP2K2

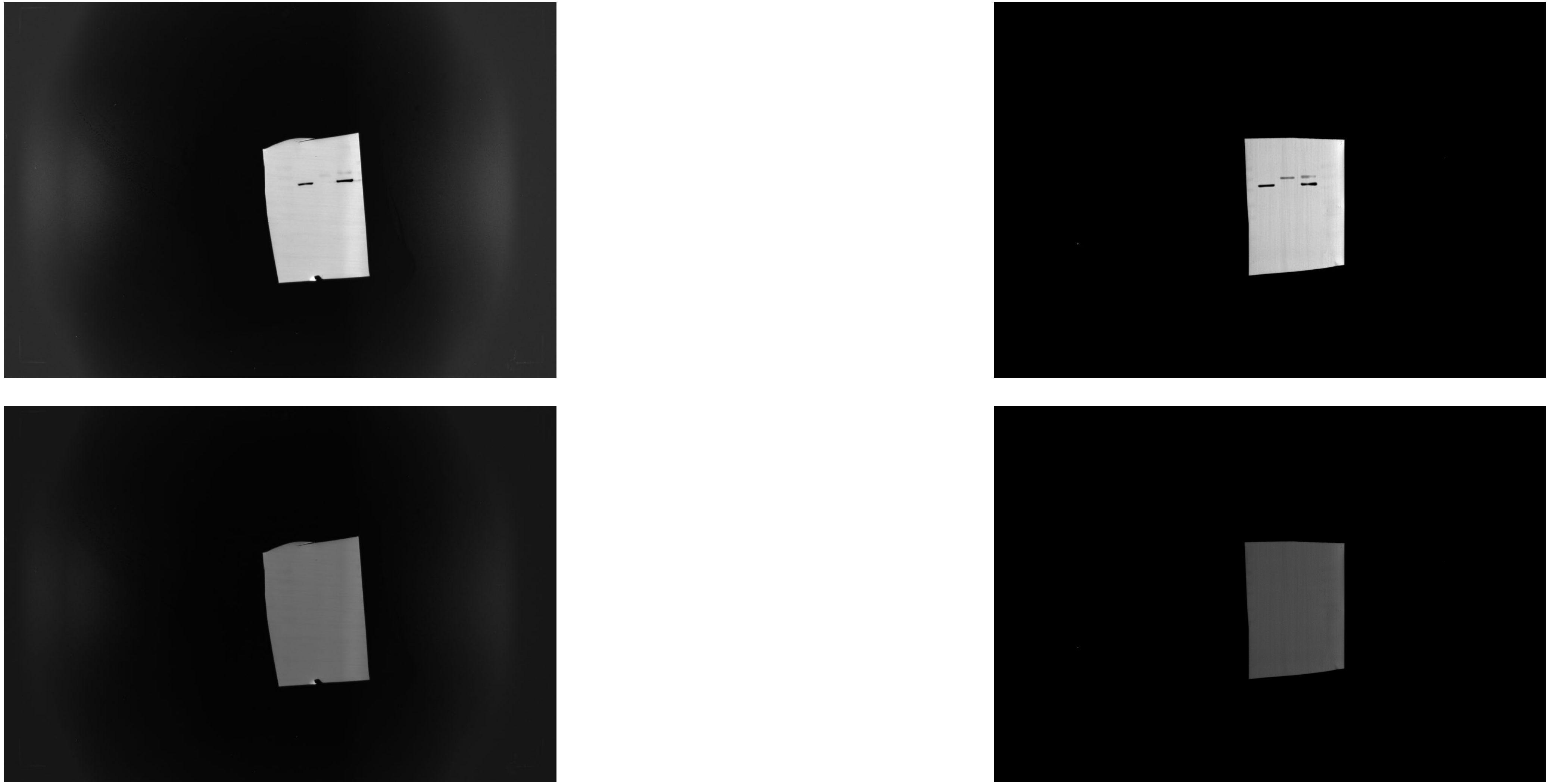

Fig. 3I

AMC-HN-8

TU212

VHL

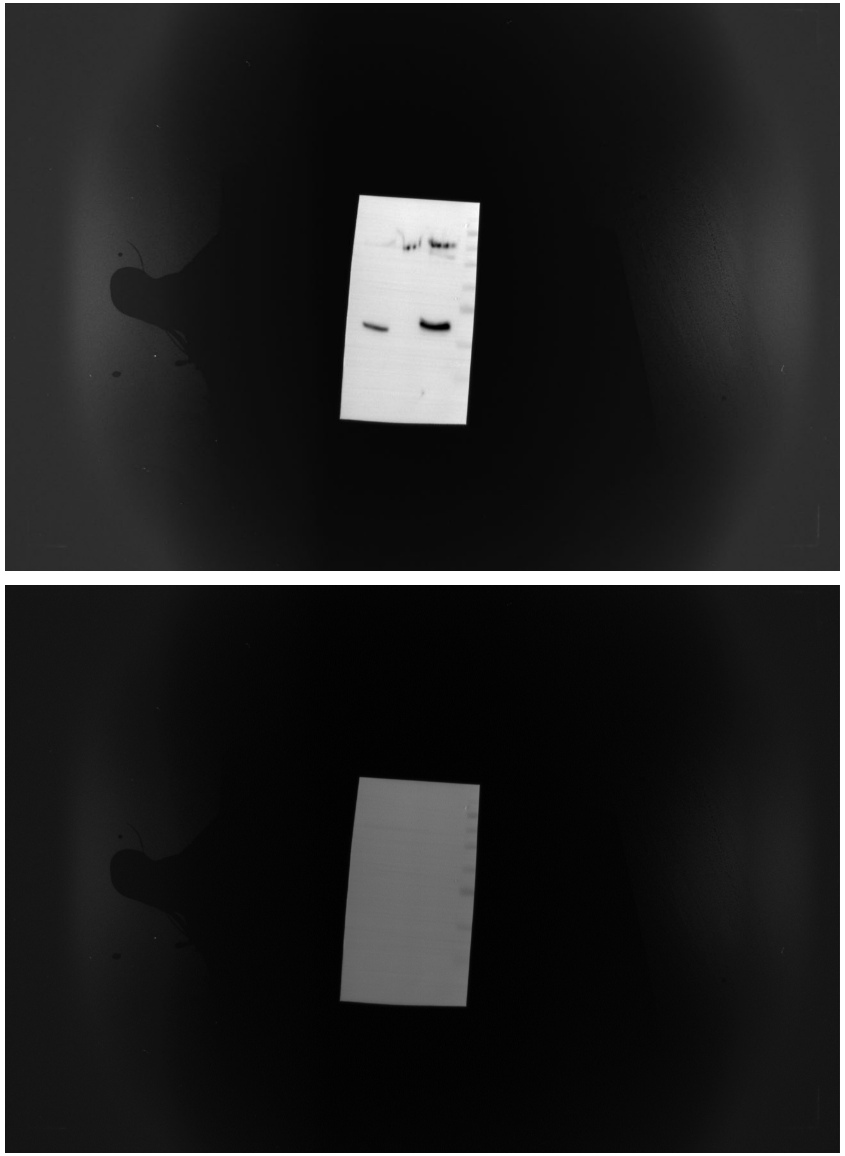

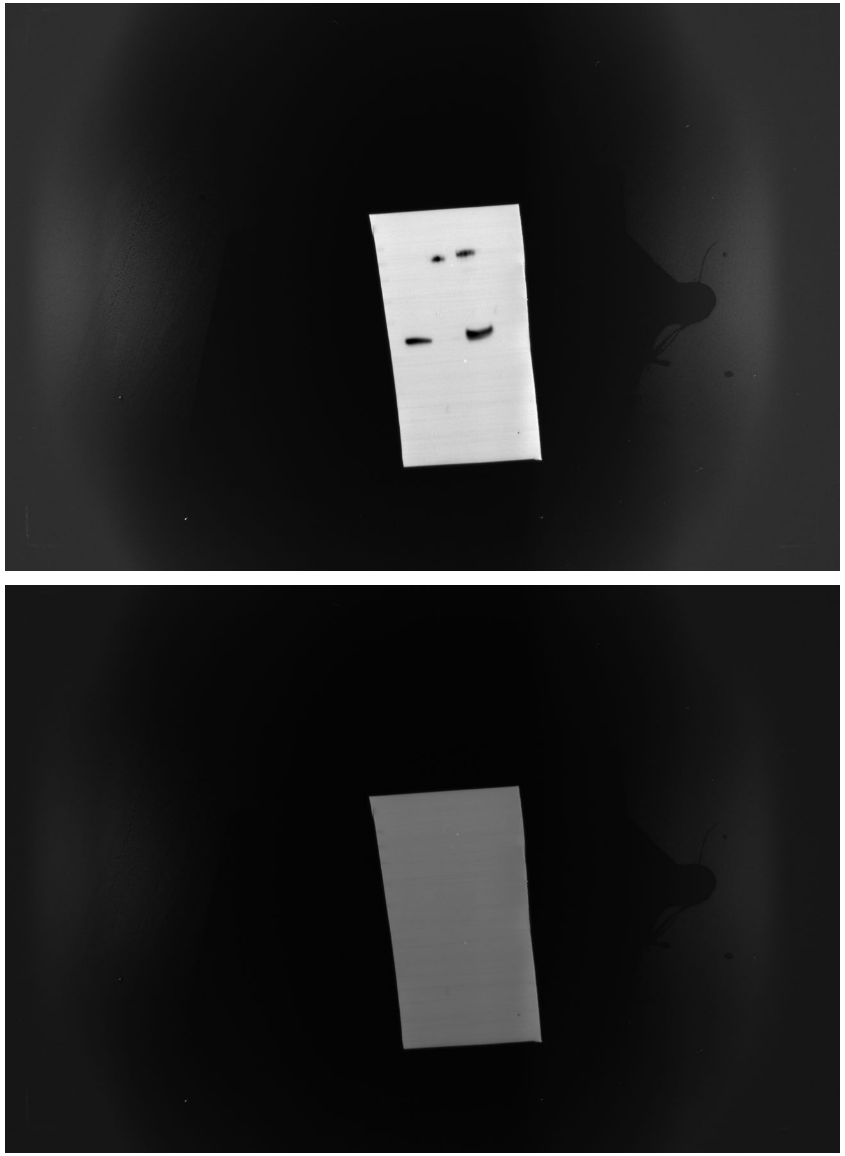

Fig. 3J

AMC-HN-8 TU212

MAP2K2

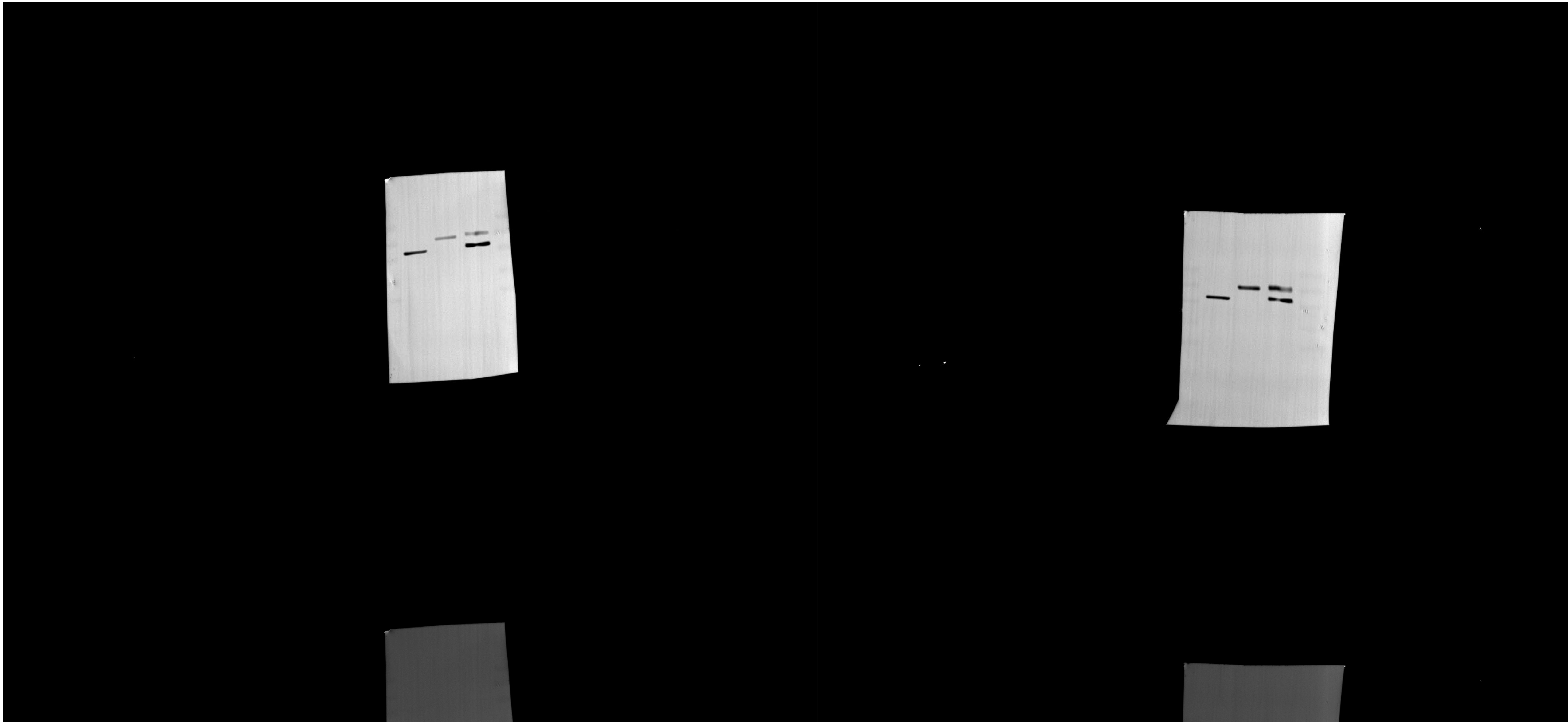

Fig. 3J

AMC-HN-8 **TU212**

VHL

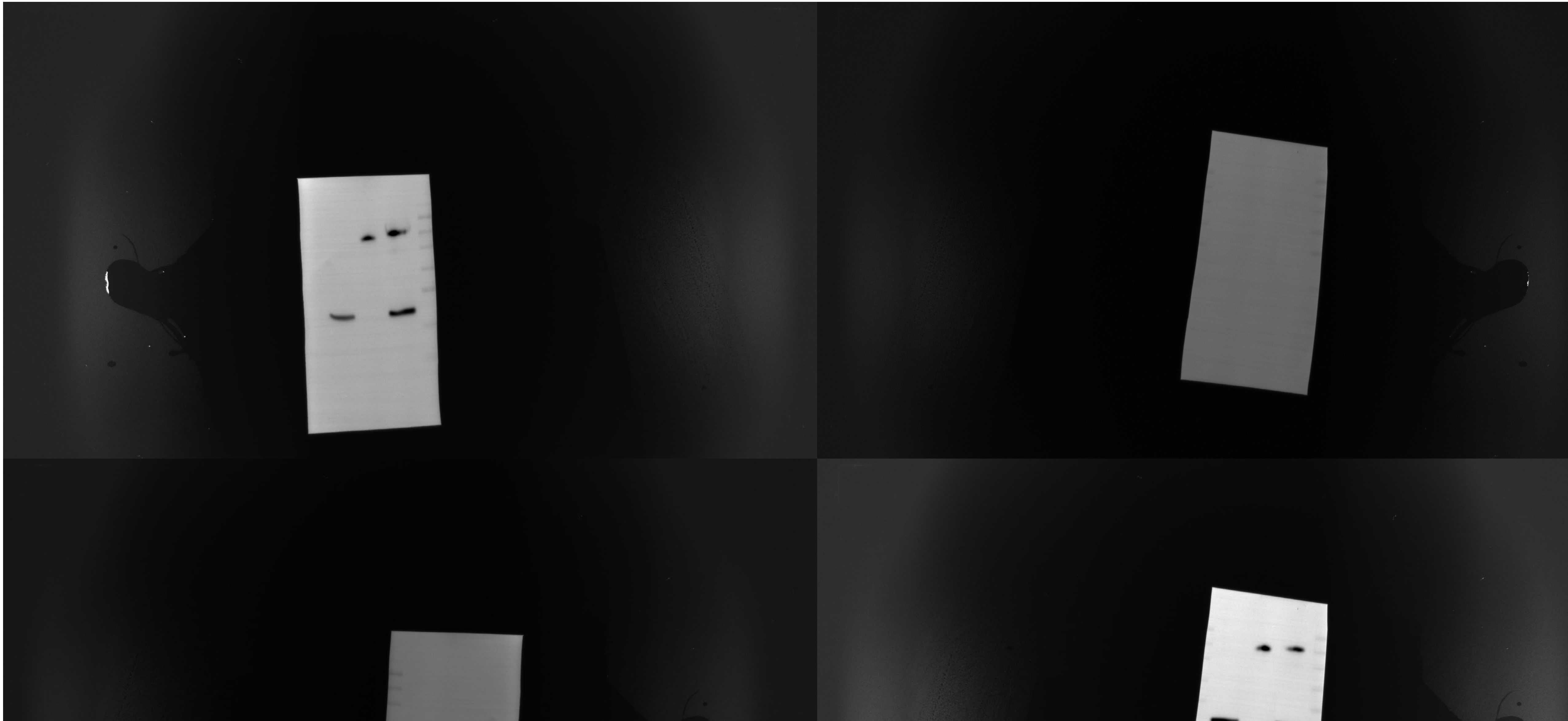

Fig. 3K

AMC-HN-8 Input EIF3B

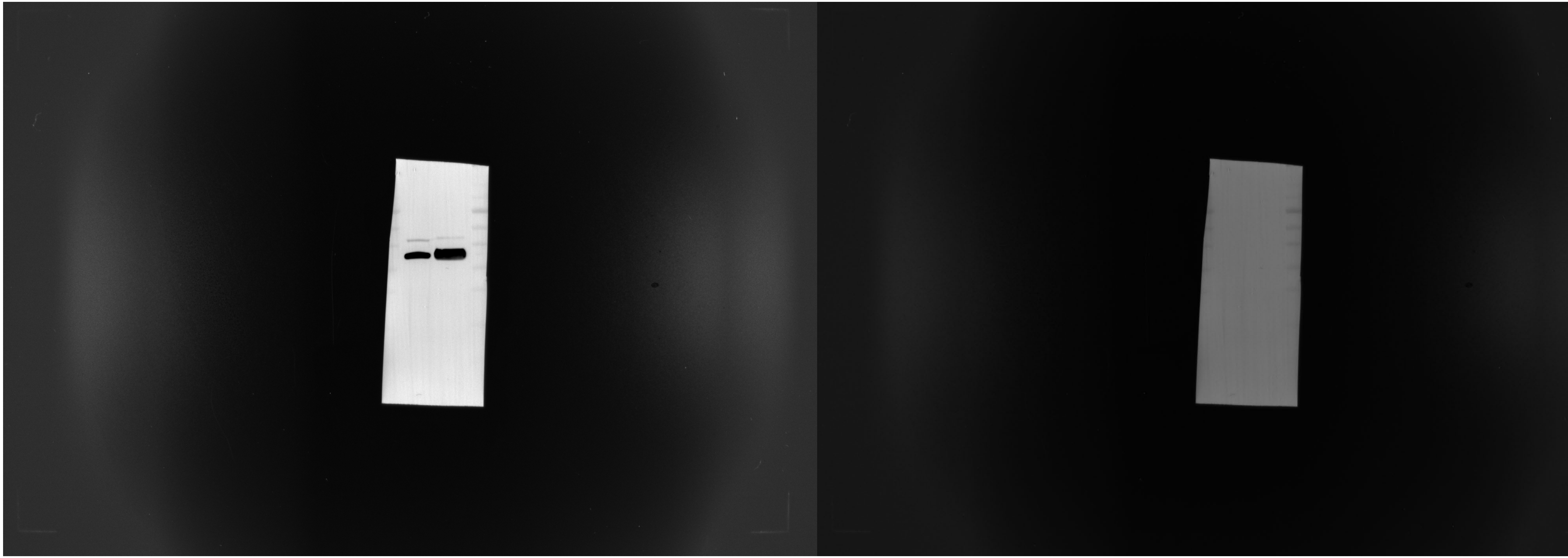

Fig. 3K

AMC-HN-8 Input GAPDH

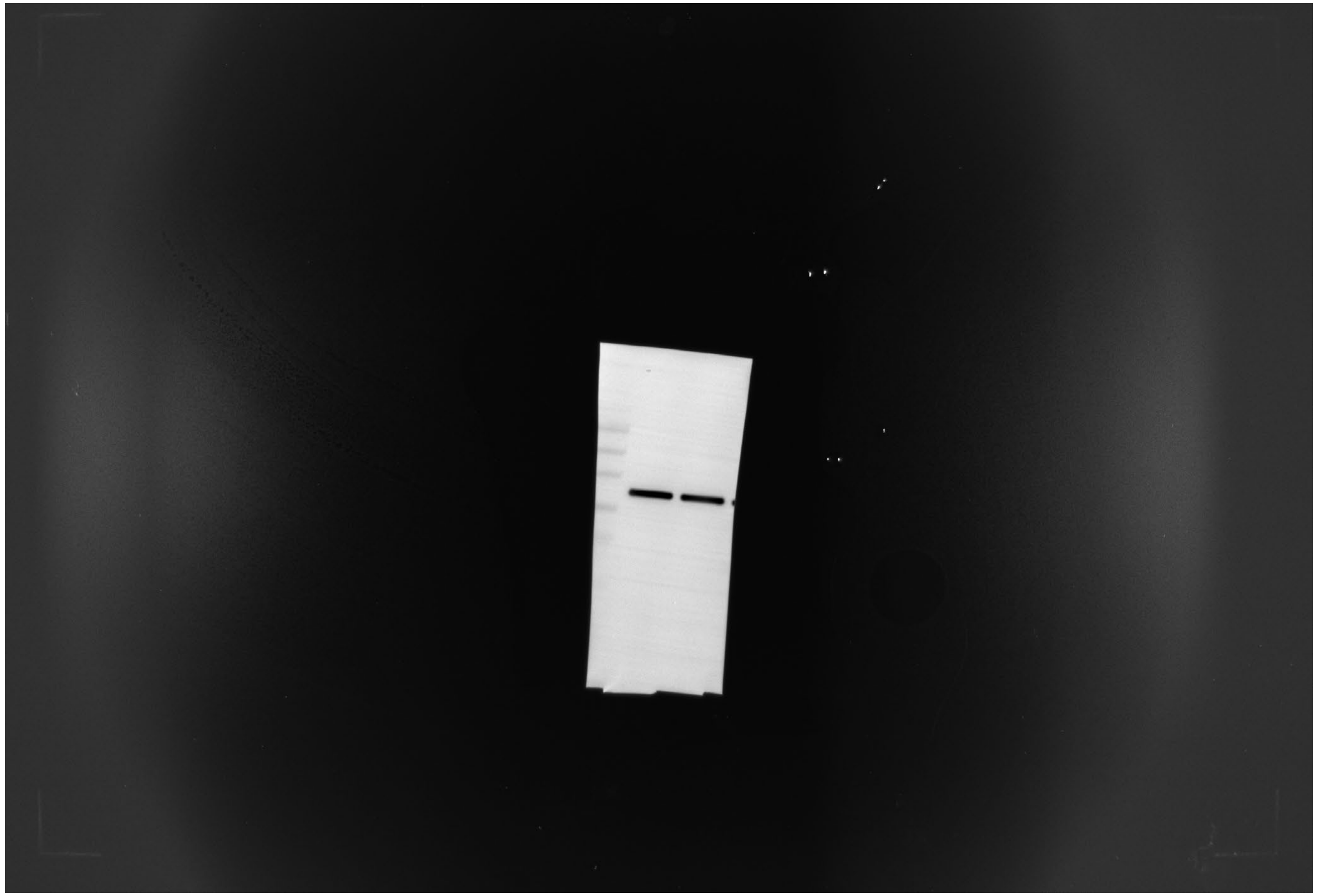

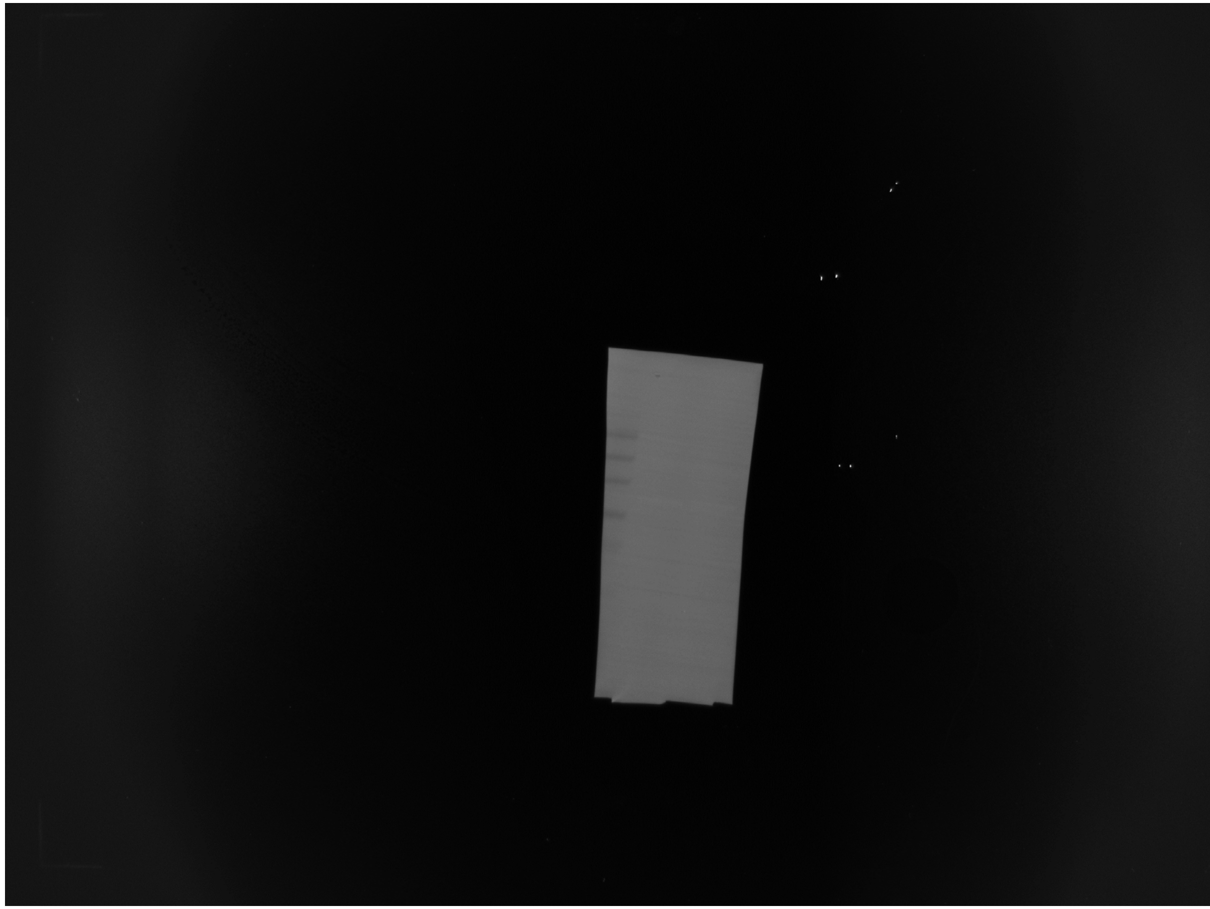

Fig. 3K

AMC-HN-8 **Input** **MAP2K2**

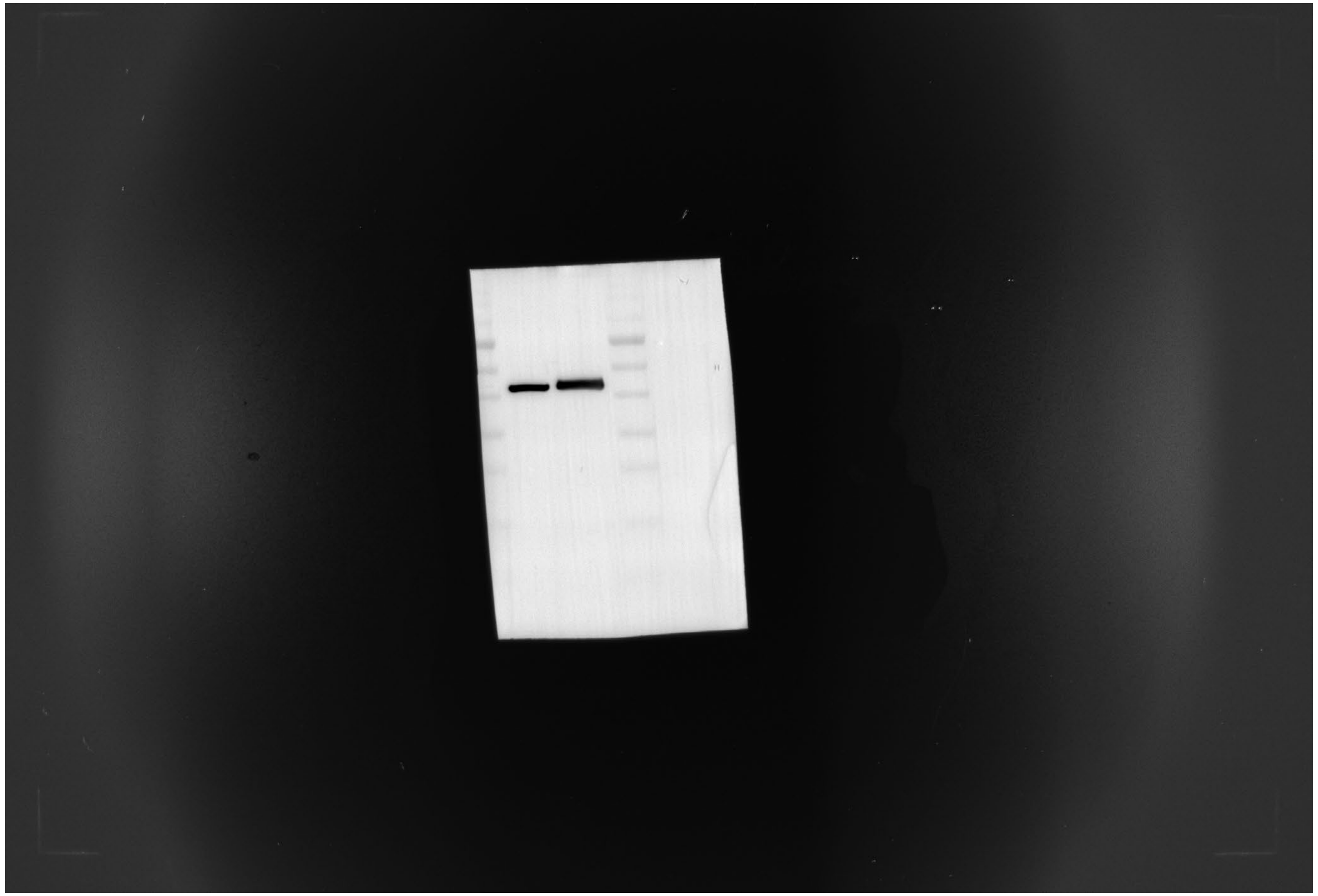

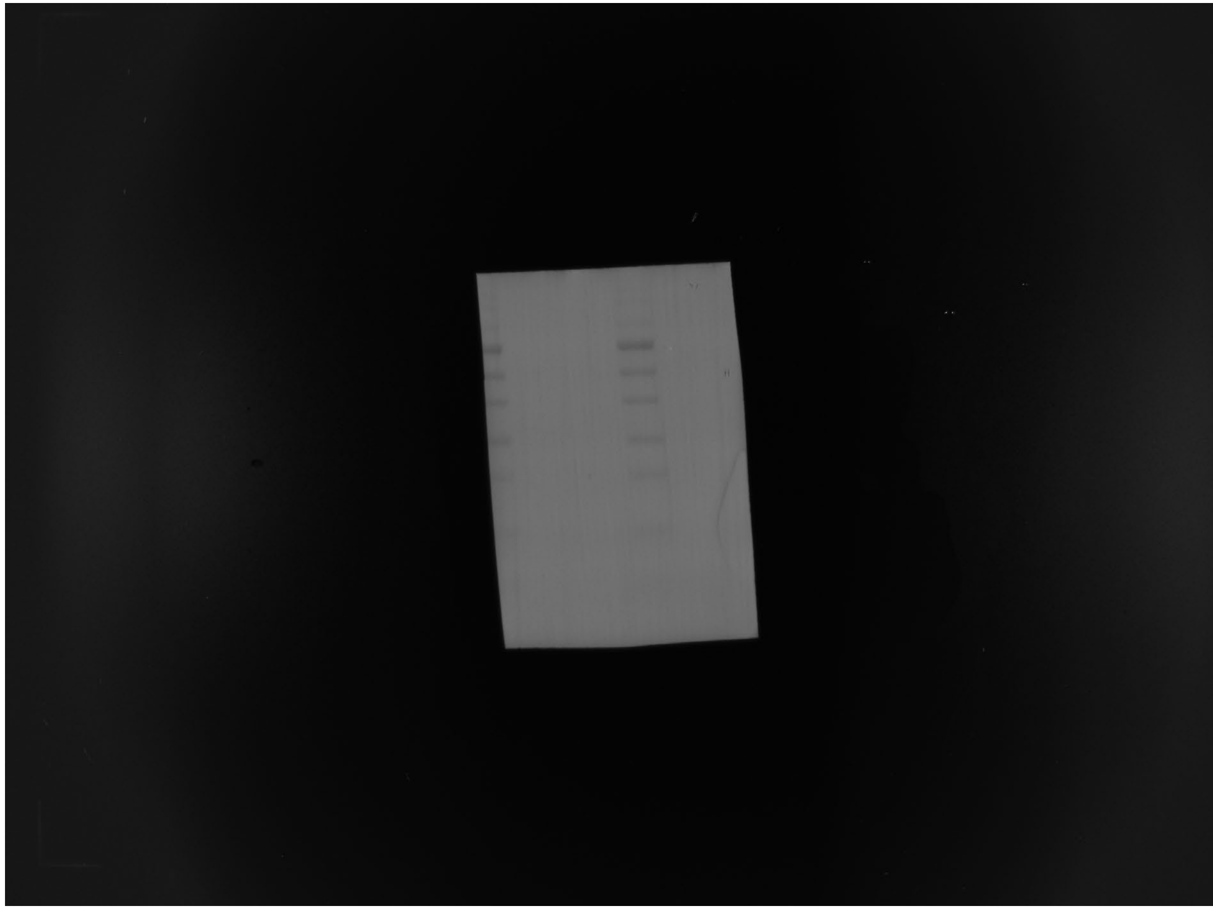

Fig. 3K

AMC-HN-8 Input VHL

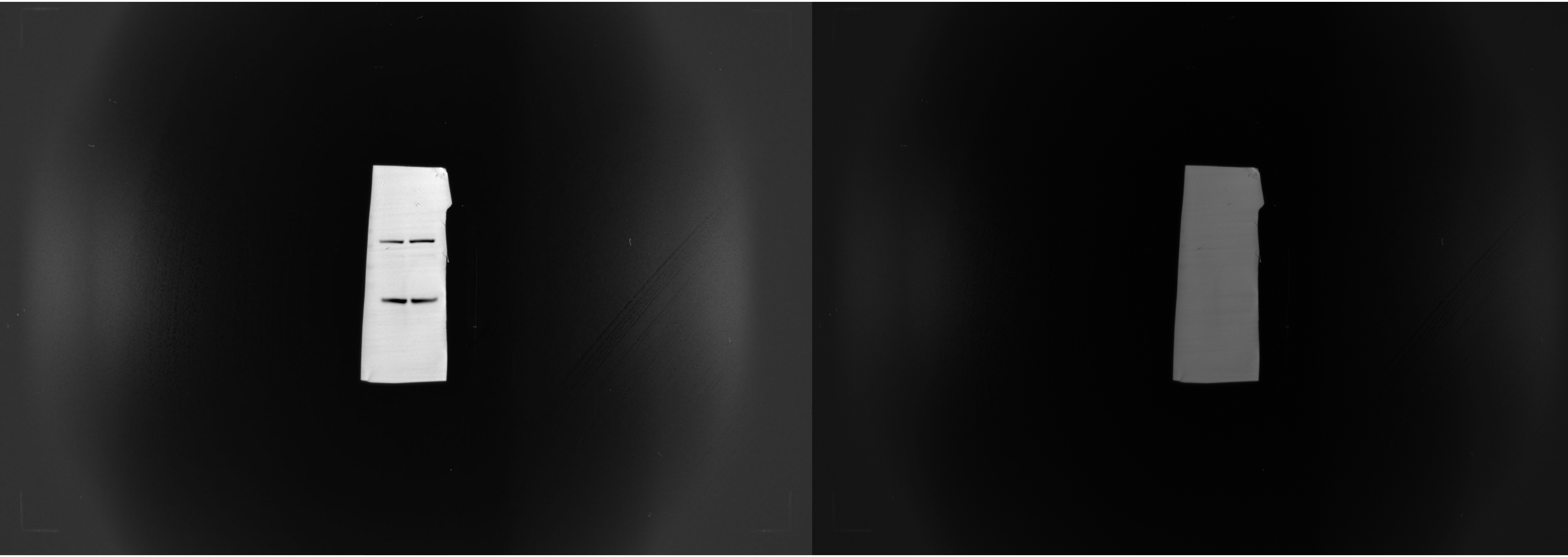

Fig. 3K

AMC-HN-8 **Ip** **EIF3B**

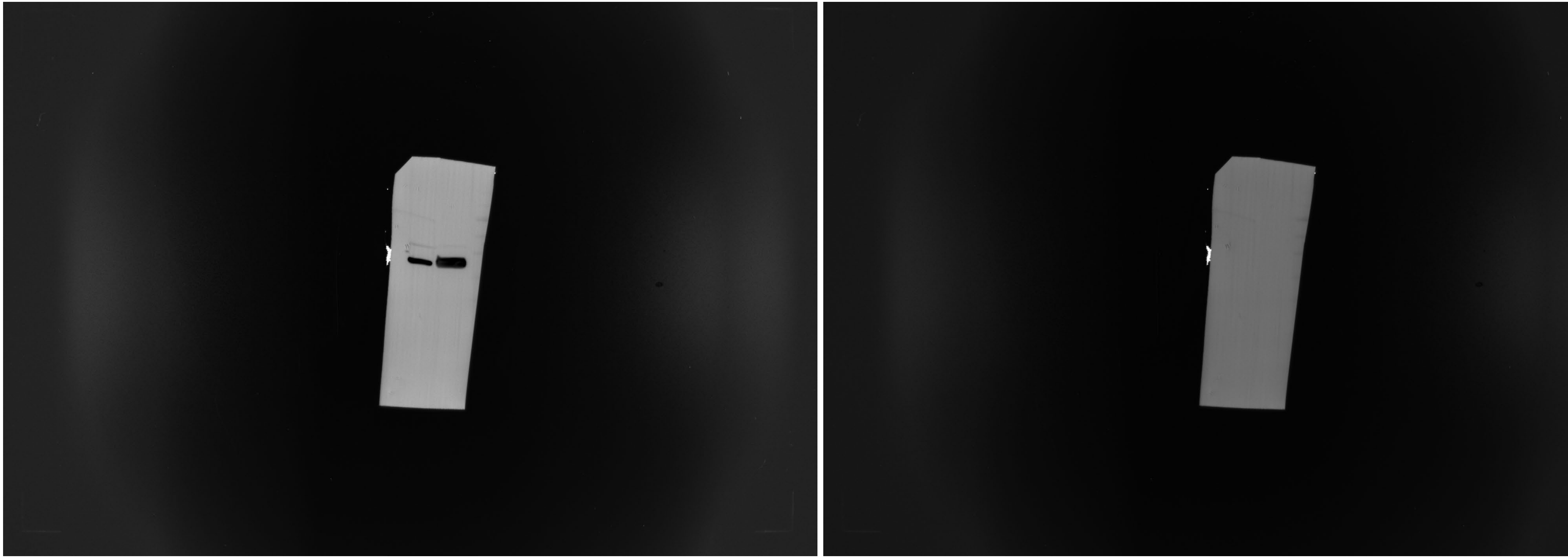

Fig. 3K

AMC-HN-8 Ip MAP2K2

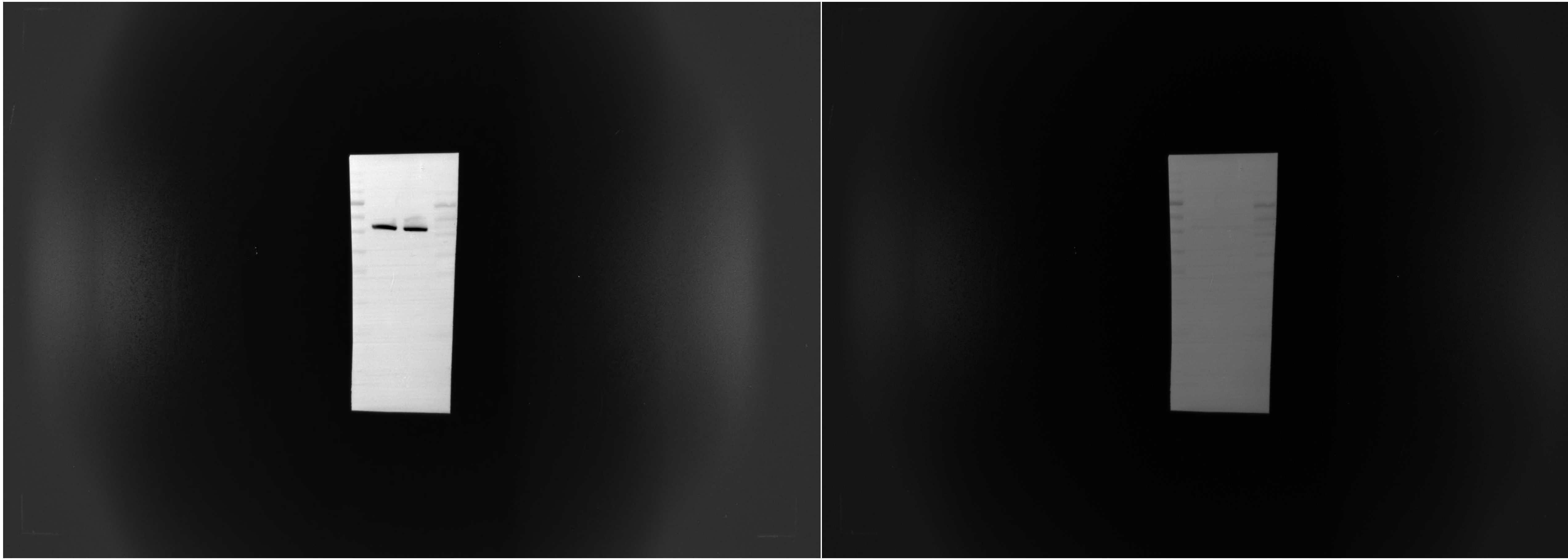

Fig. 3K

AMC-HN-8 Ip VHL

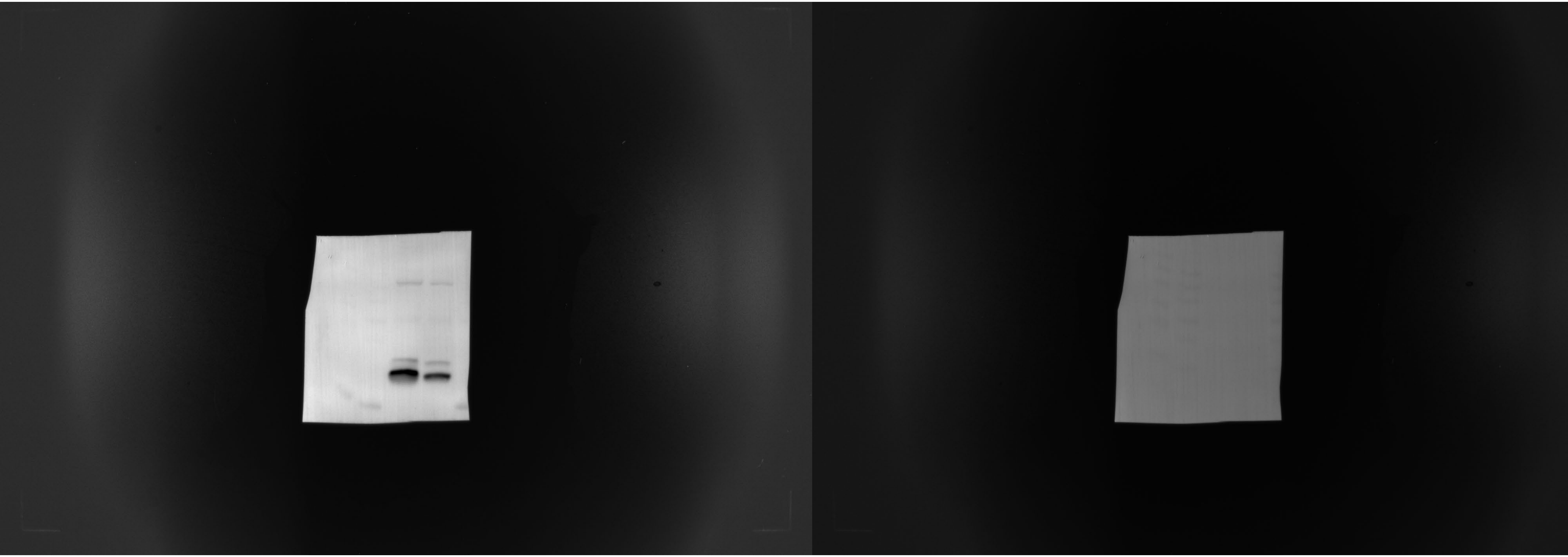

Fig. 3K

TU212 **Input** **EIF3B**

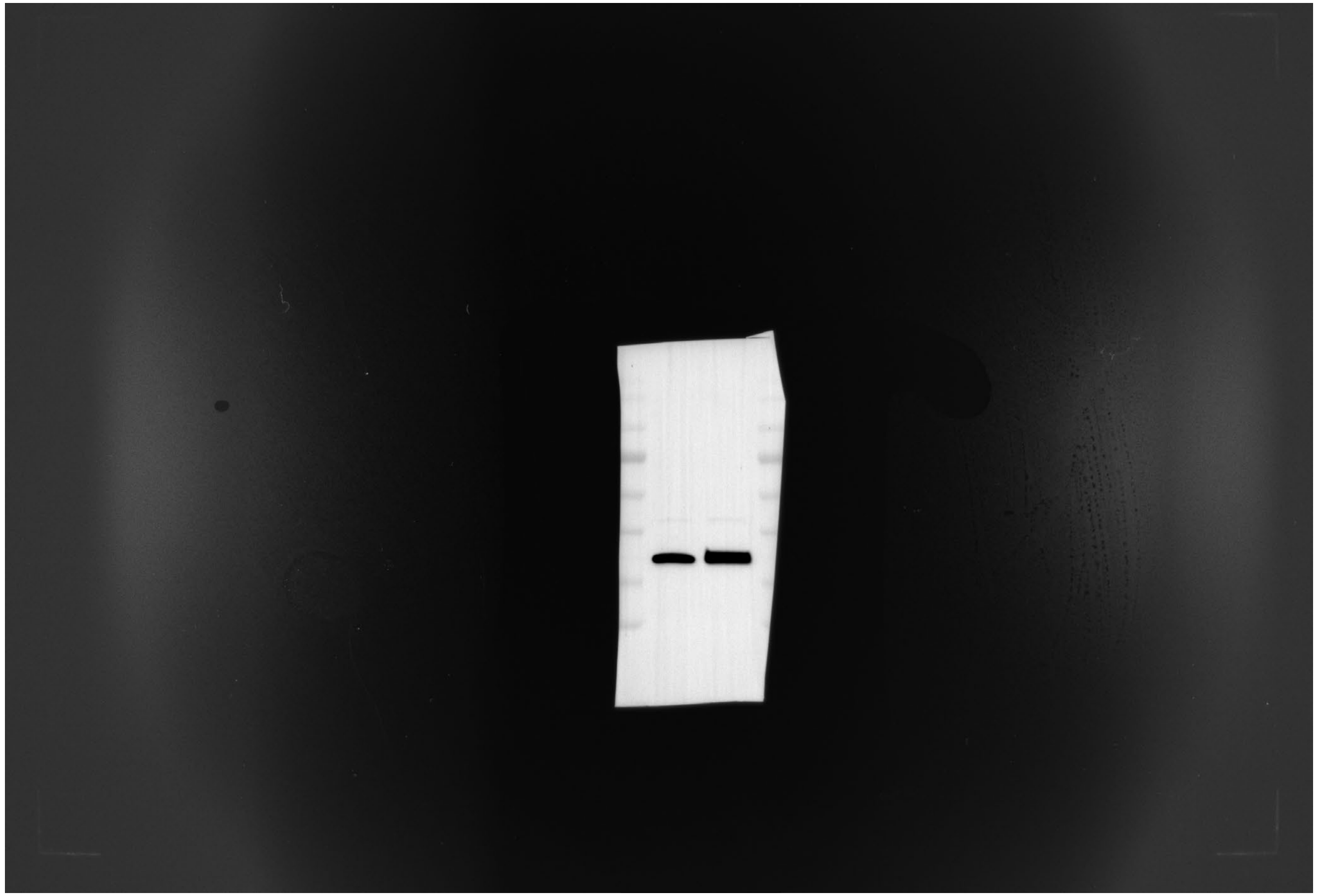

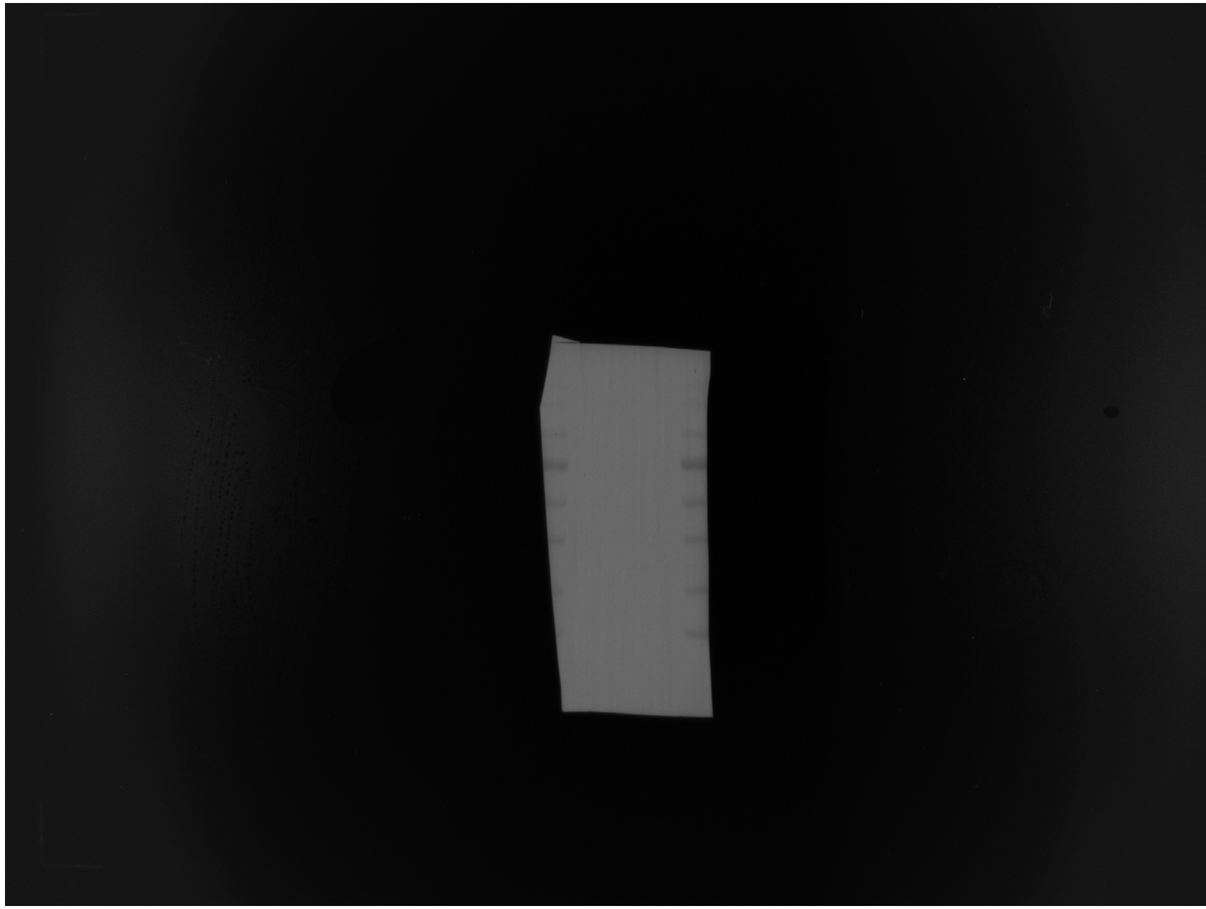

Fig. 3K

TU212 Input GAPDH

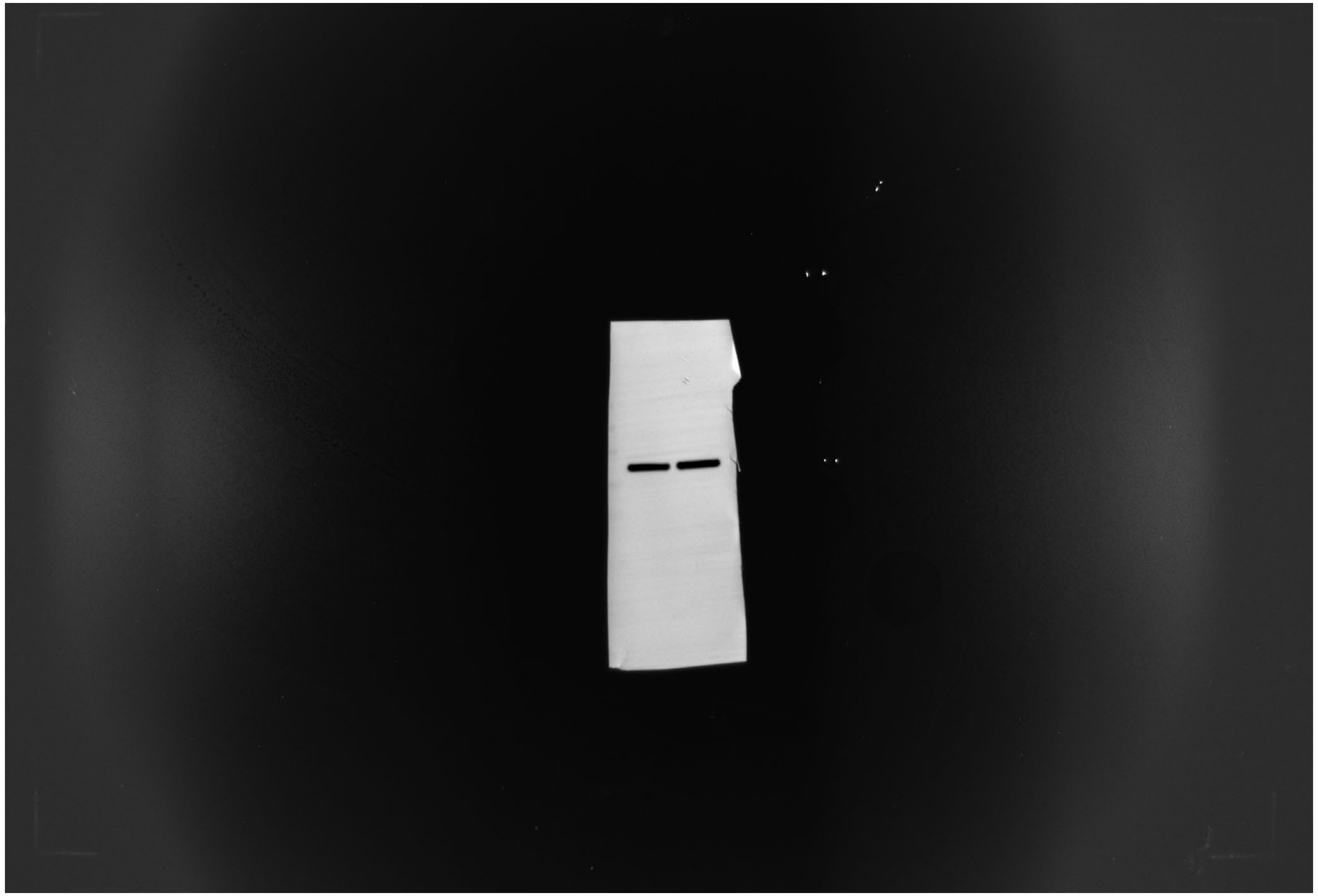

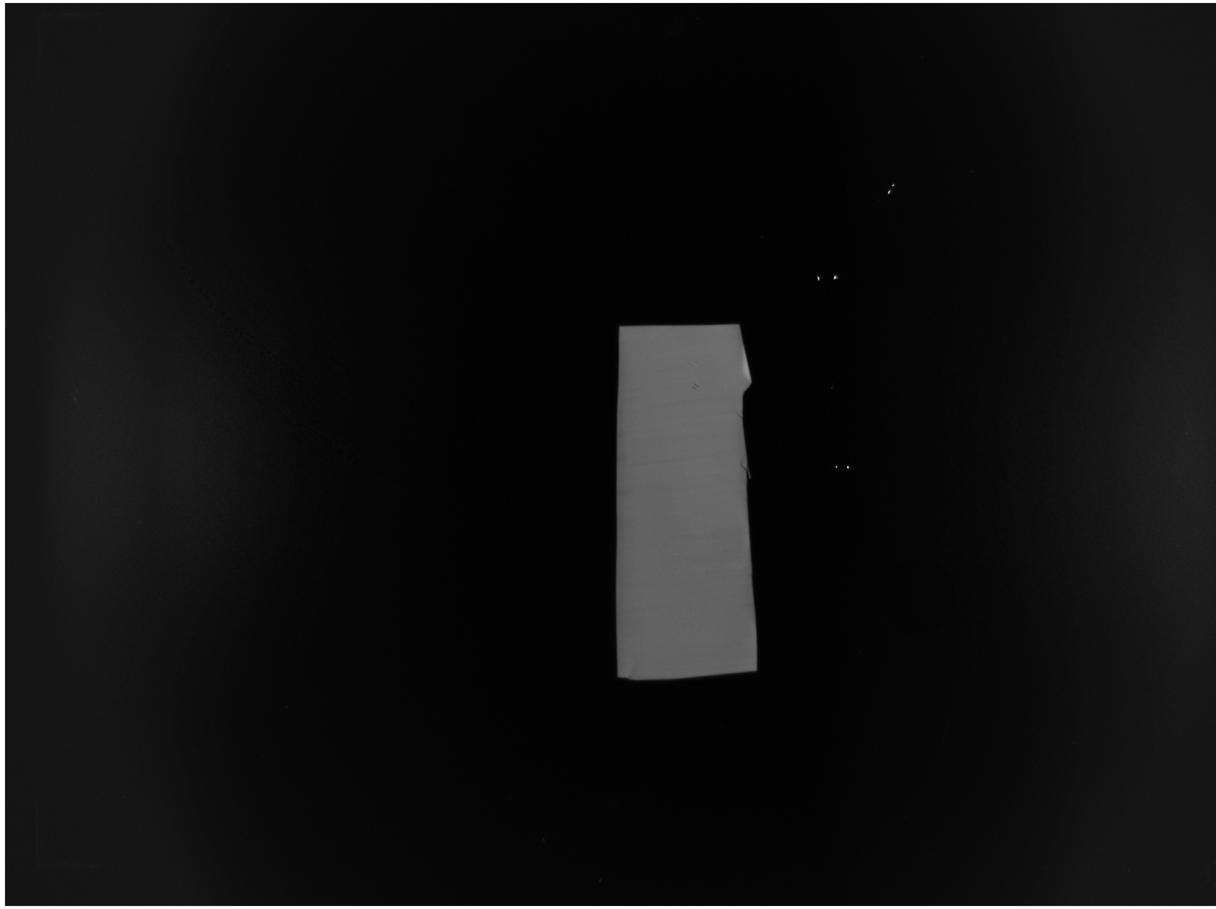

Fig. 3K

TU212 Input MAP2K2

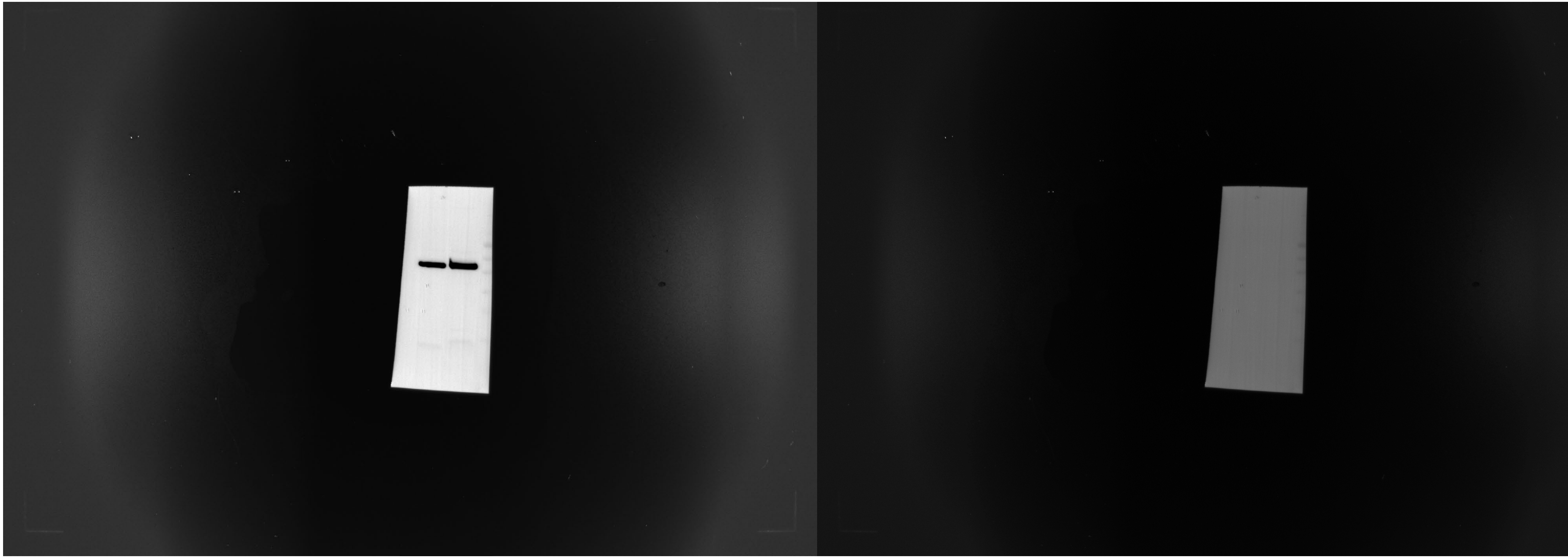

Fig. 3K

TU212 Input VHL

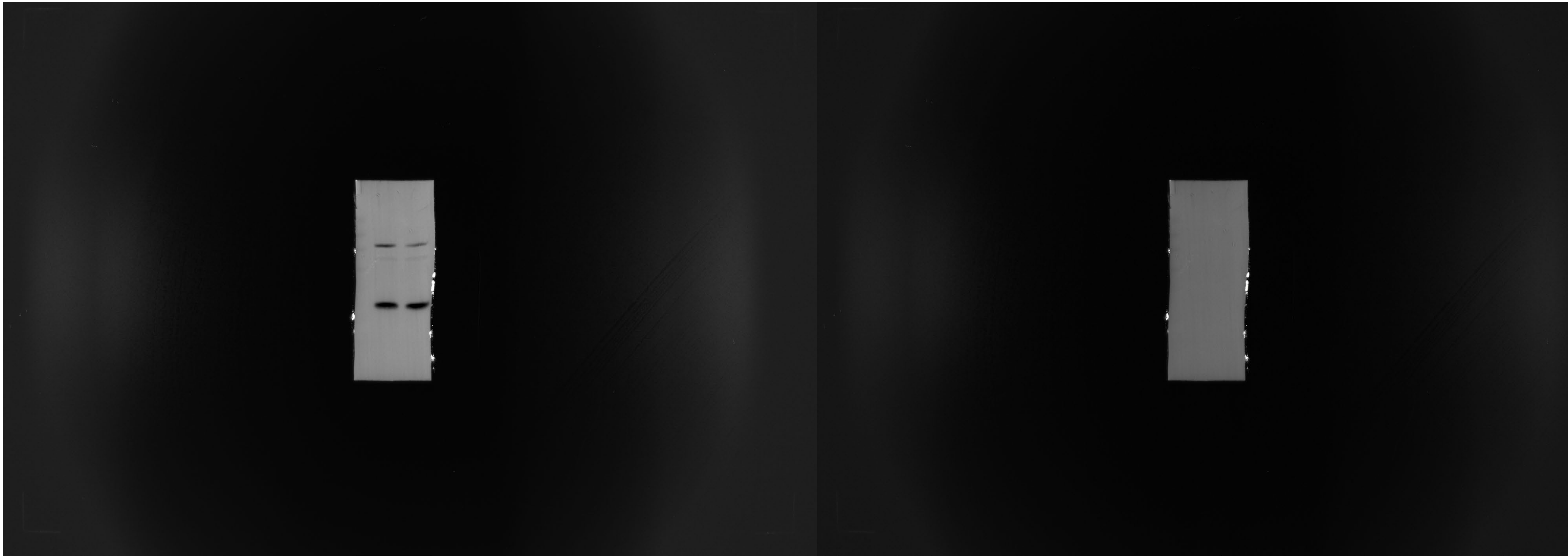

Fig. 3K

TU212 IP EIF3B

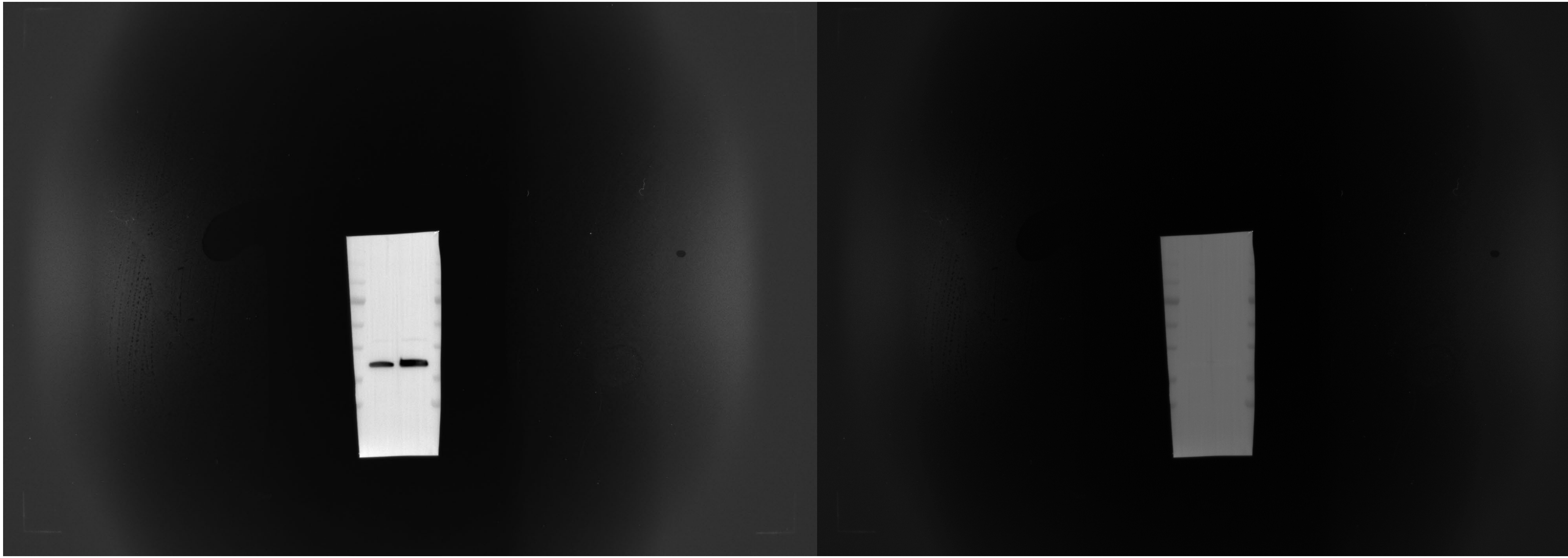

Fig. 3K

TU212 Ip MAP2K2

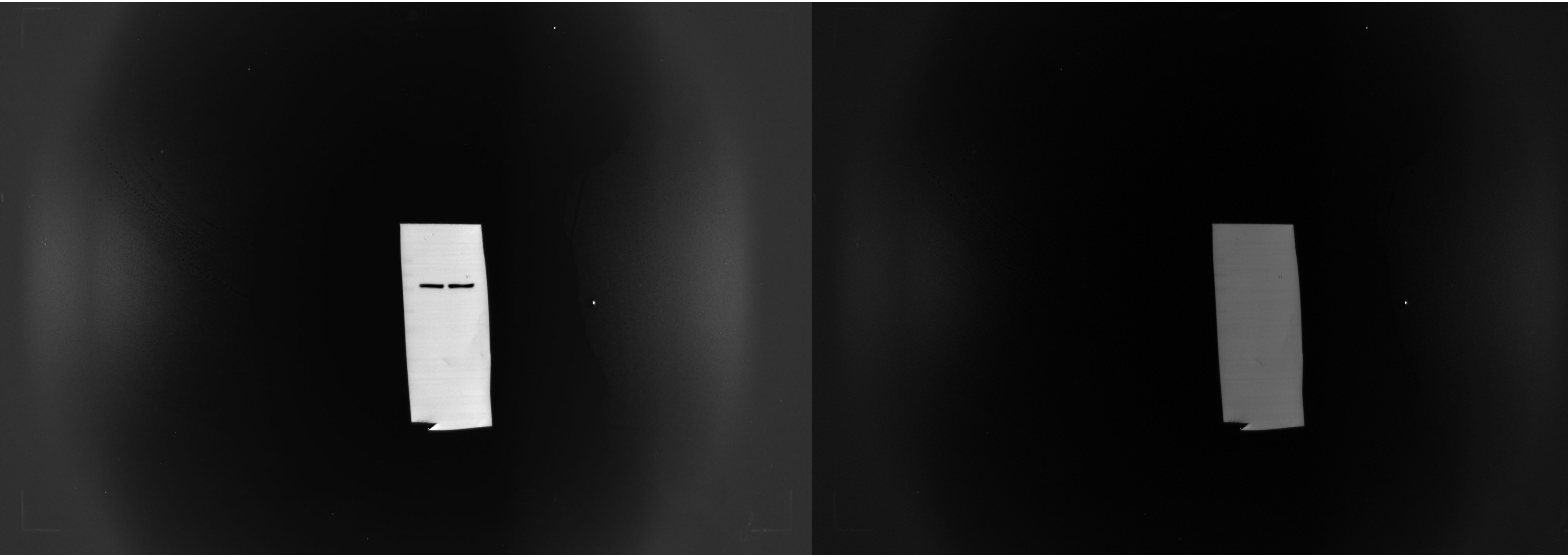

Fig. 3K

TU212 Ip VHL

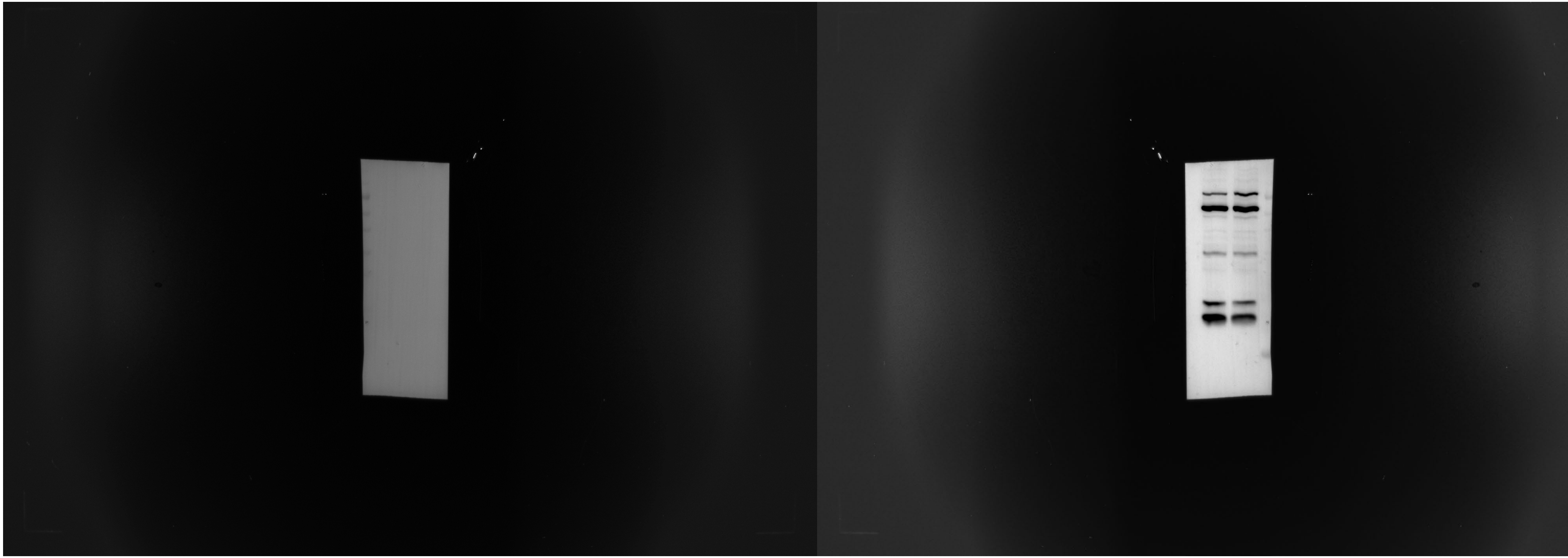

Fig. 5A

AMC-HN-8 | ERK | TU212

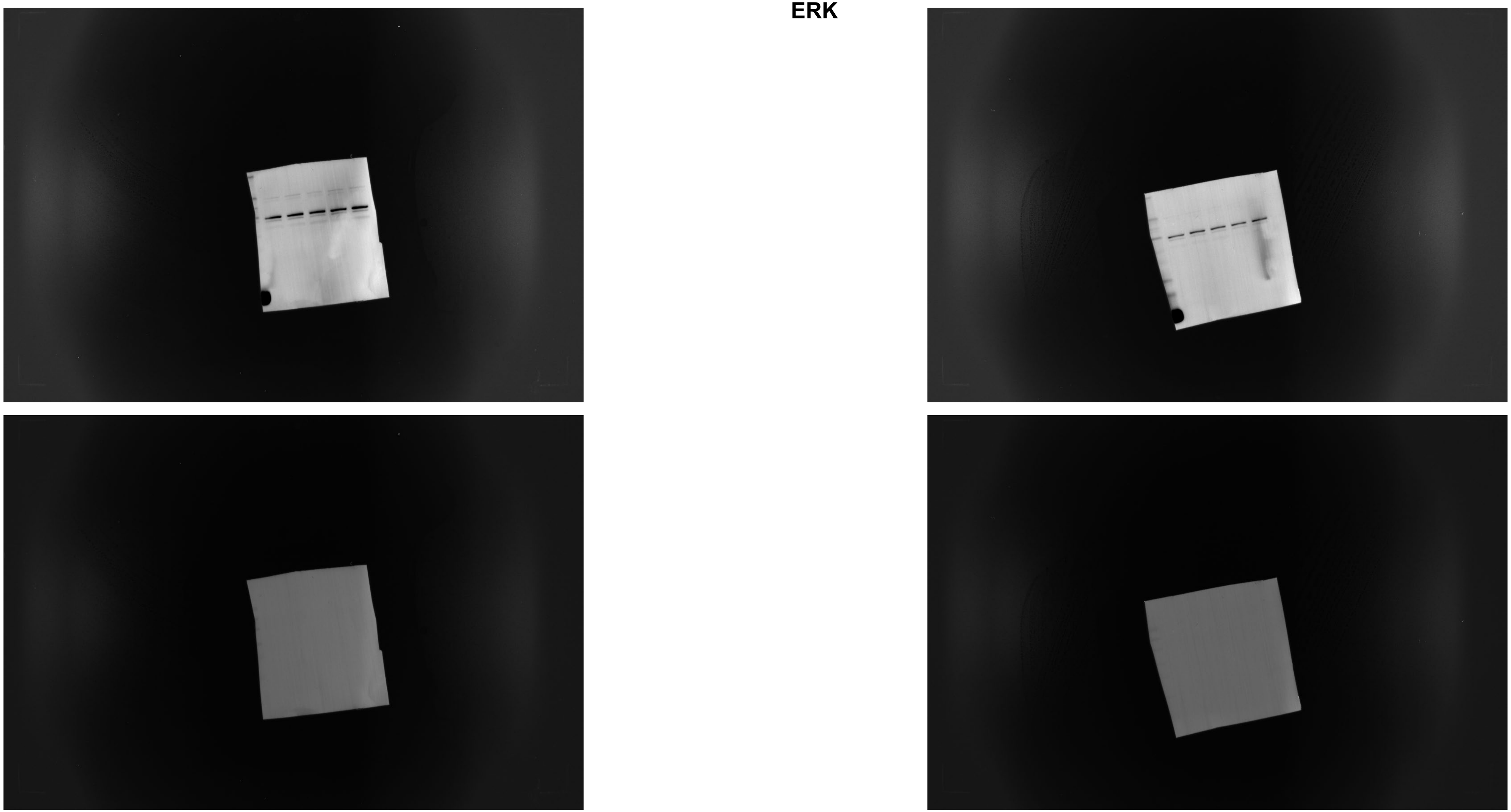

Fig. 5A

AMC-HN-8

TU212

P-ERK

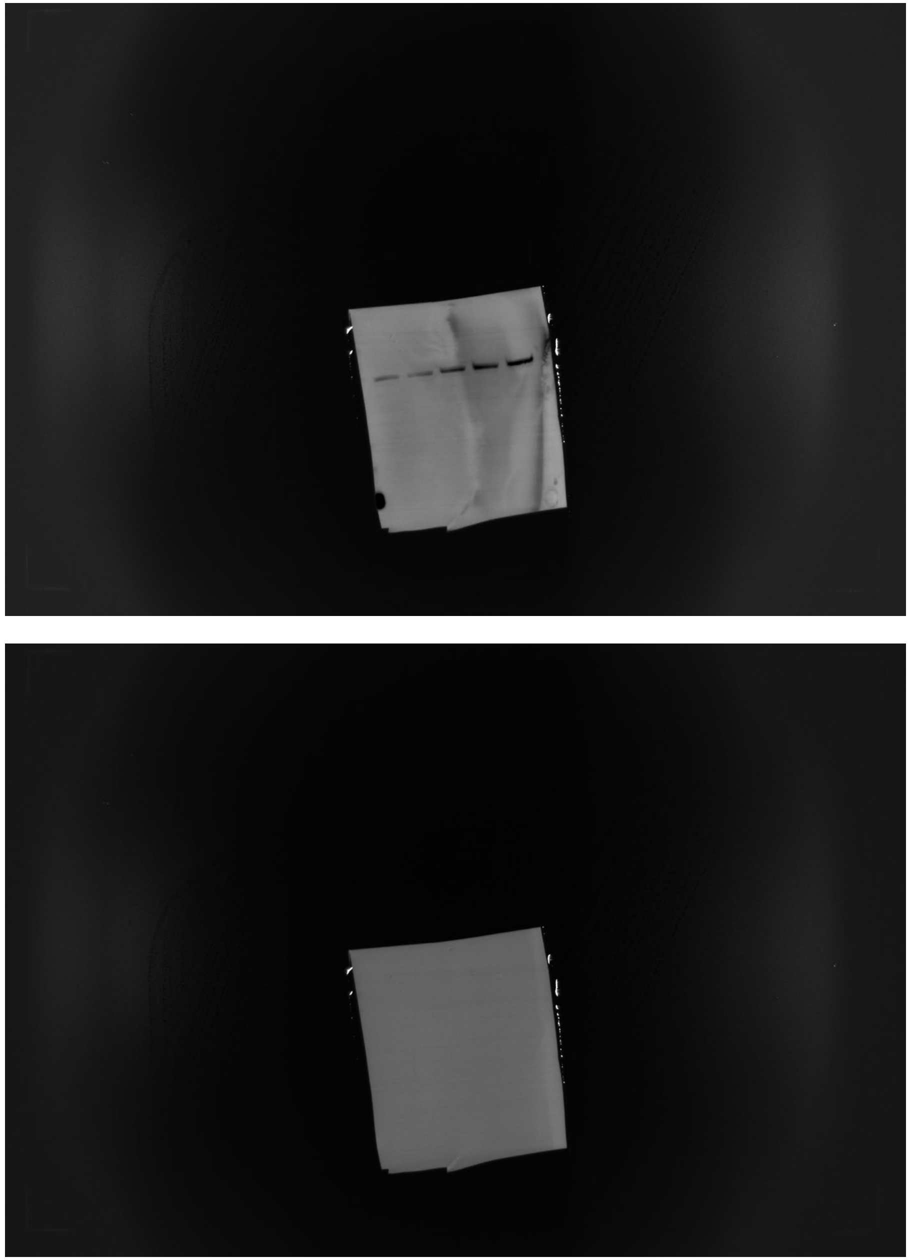

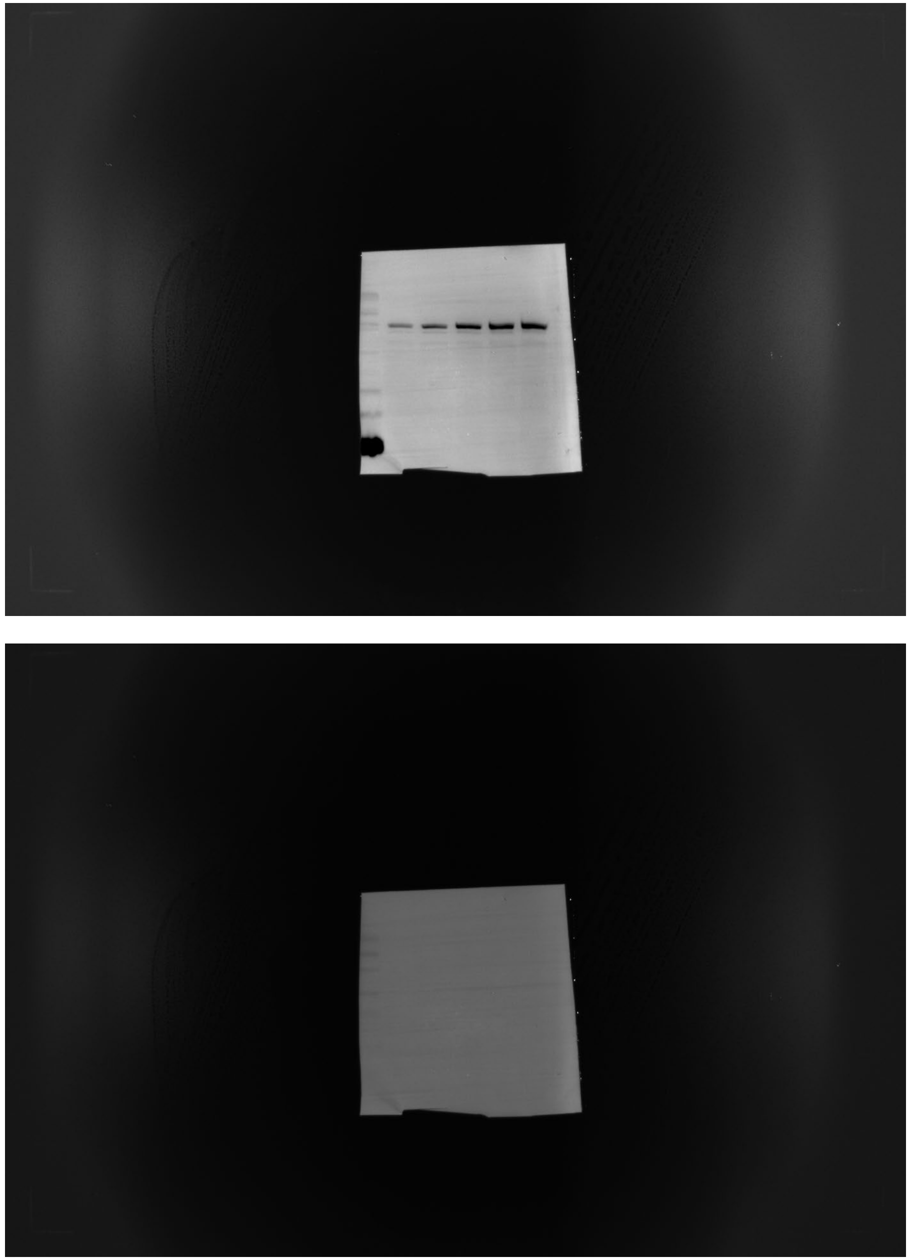

Fig. 5A

AMC-HN-8

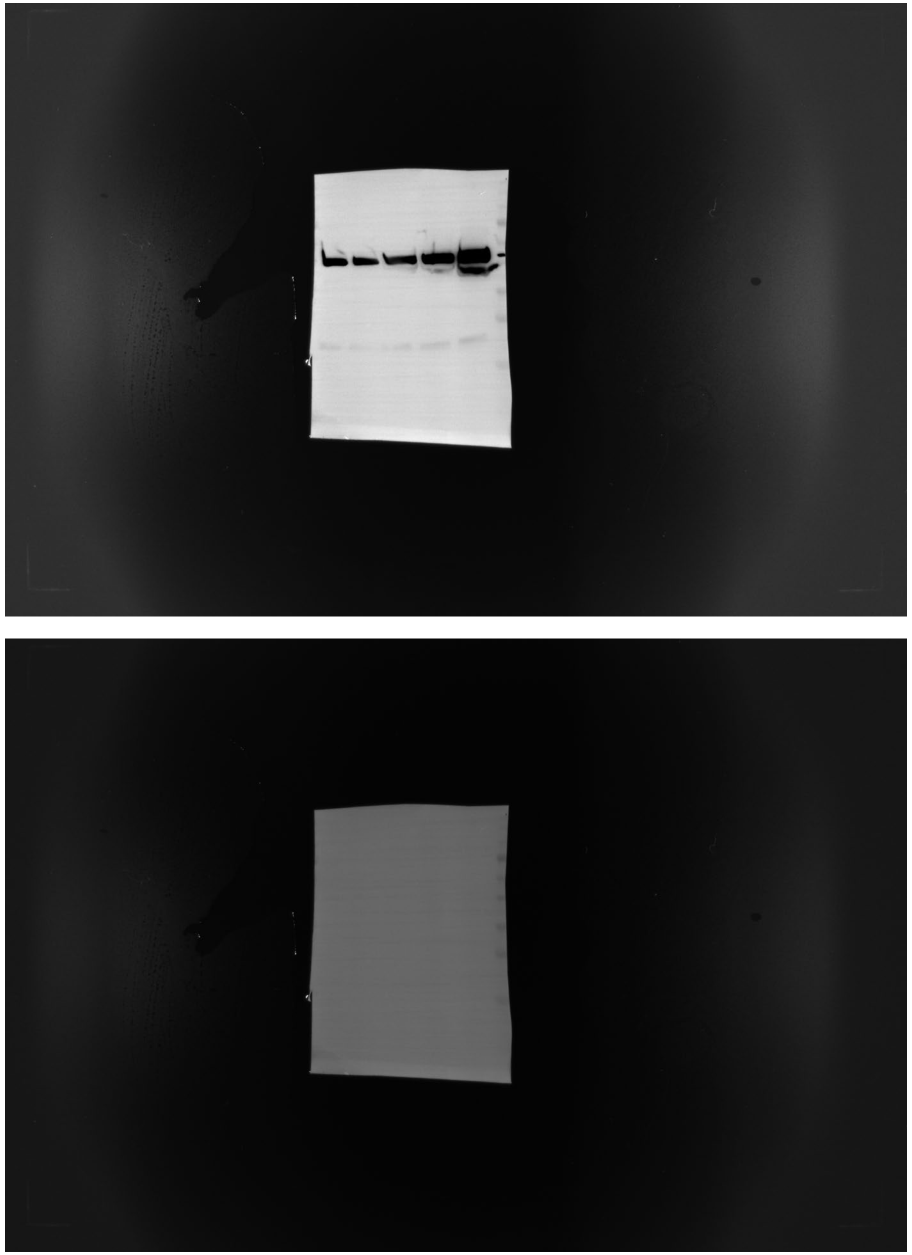

TU212

MAP2K2

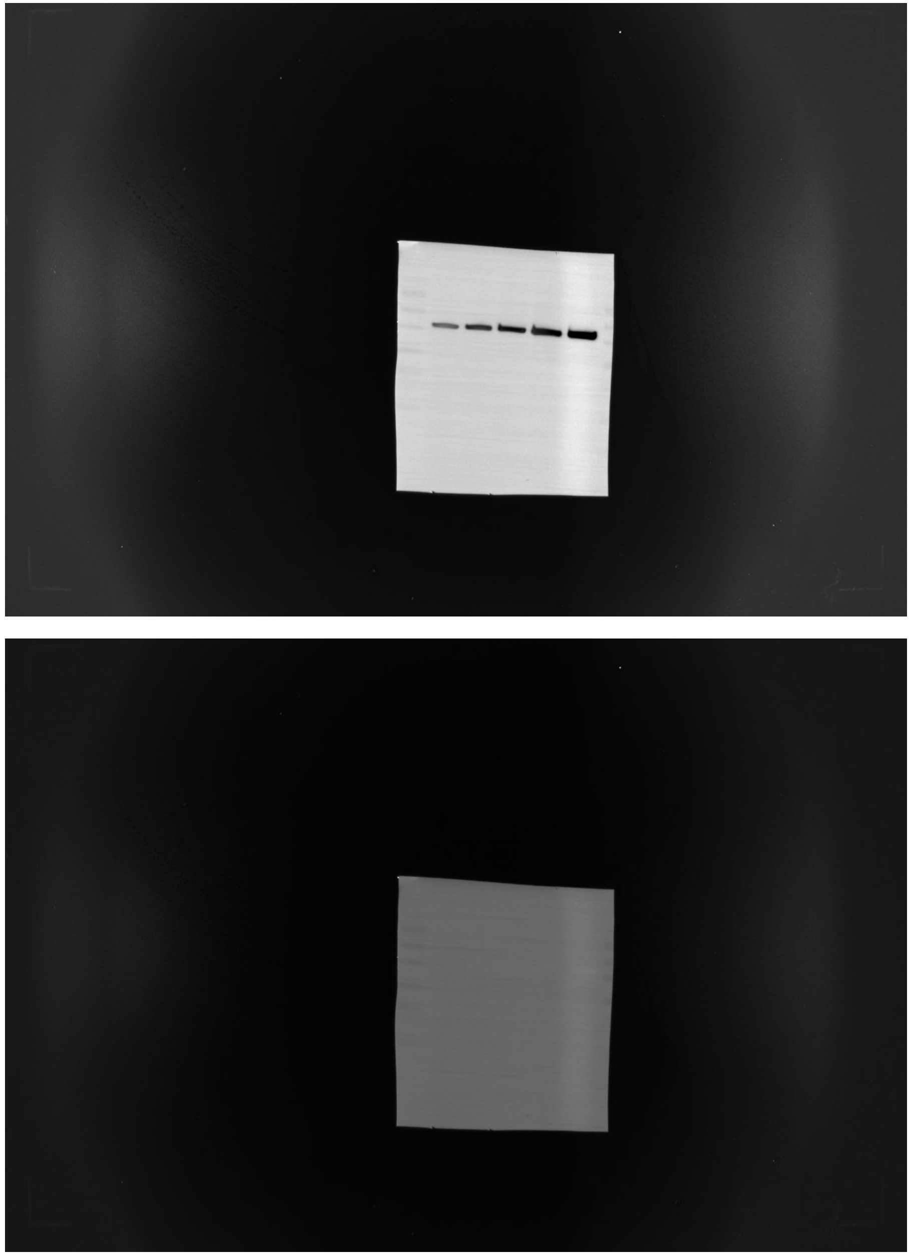

Fig. 5A

AMC-HN-8

TU212

P-MAP2K2

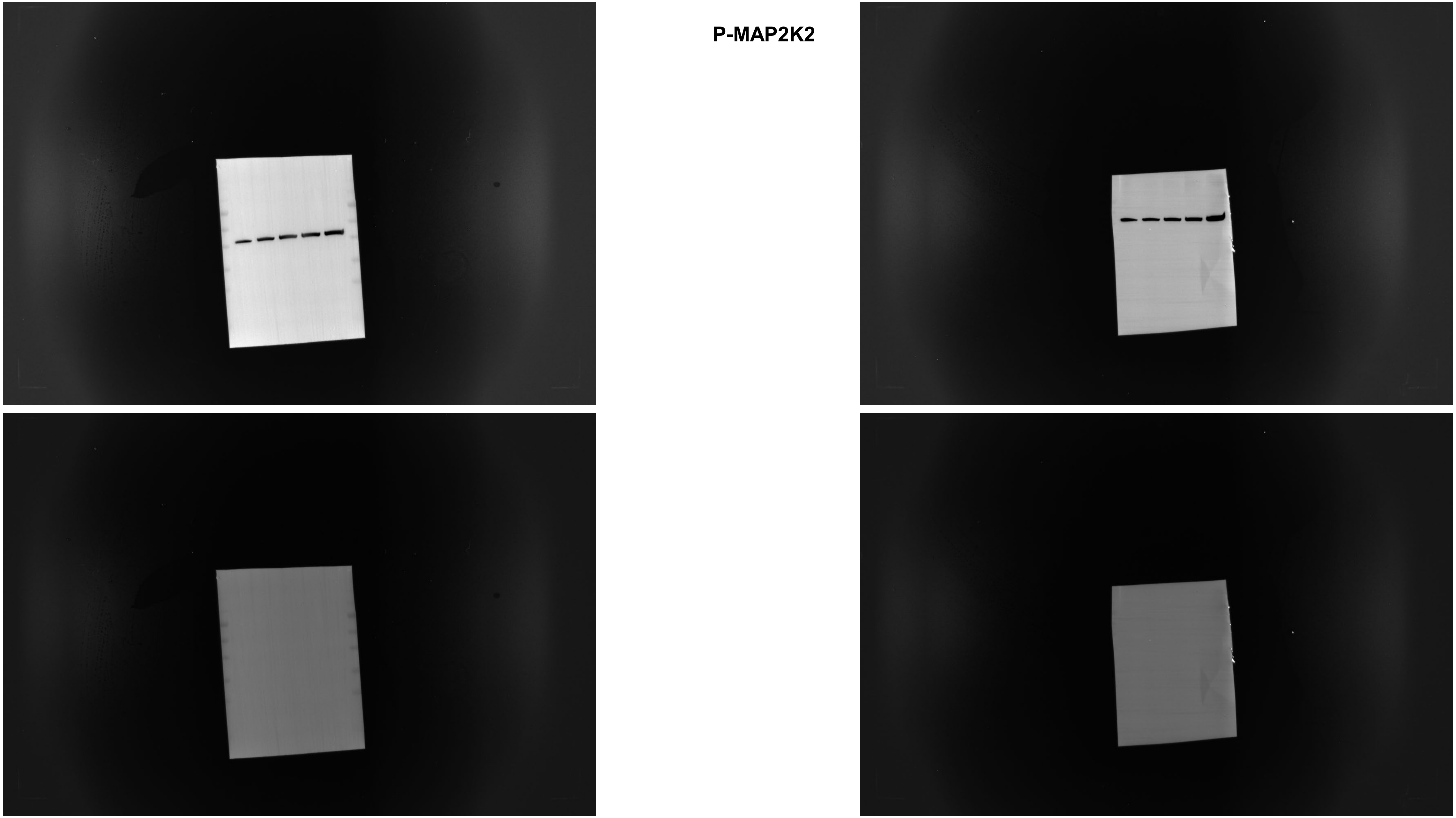

Fig. 5A

AMC-HN-8 **TU212**

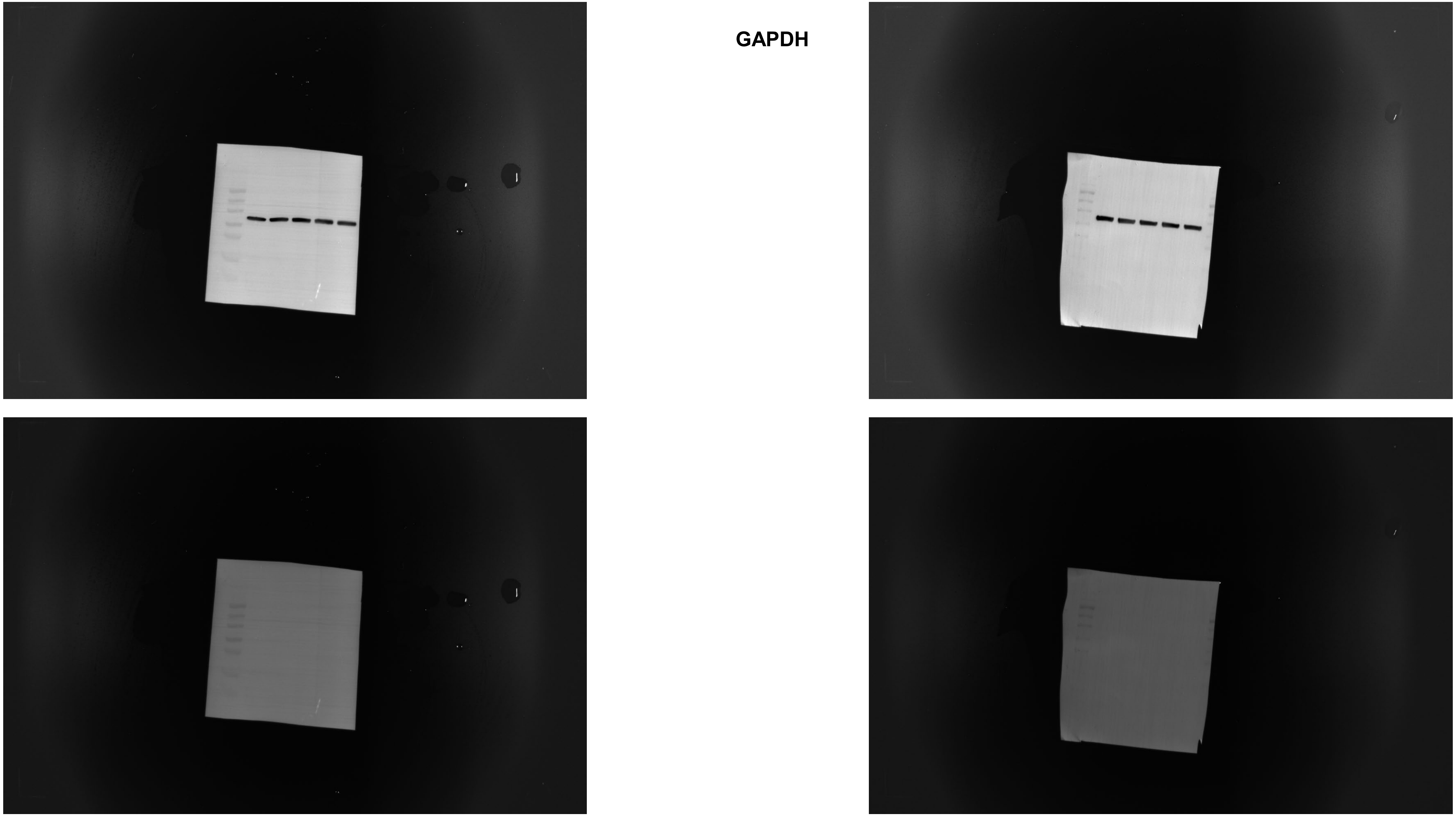

AMC-HN-8

Fig. 5B

ERK

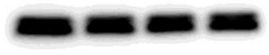

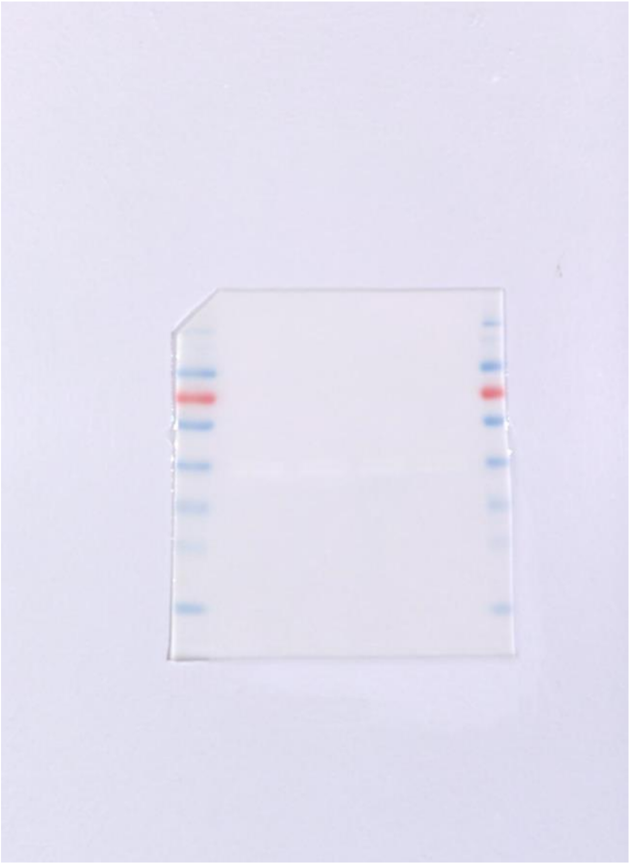

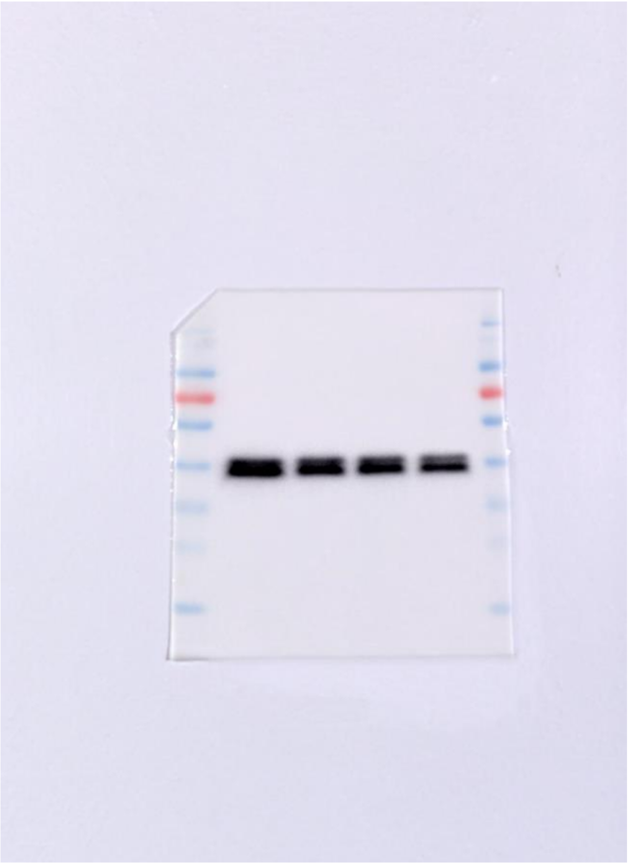

AMC-HN-8

Fig. 5B

P-ERK

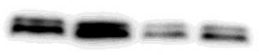

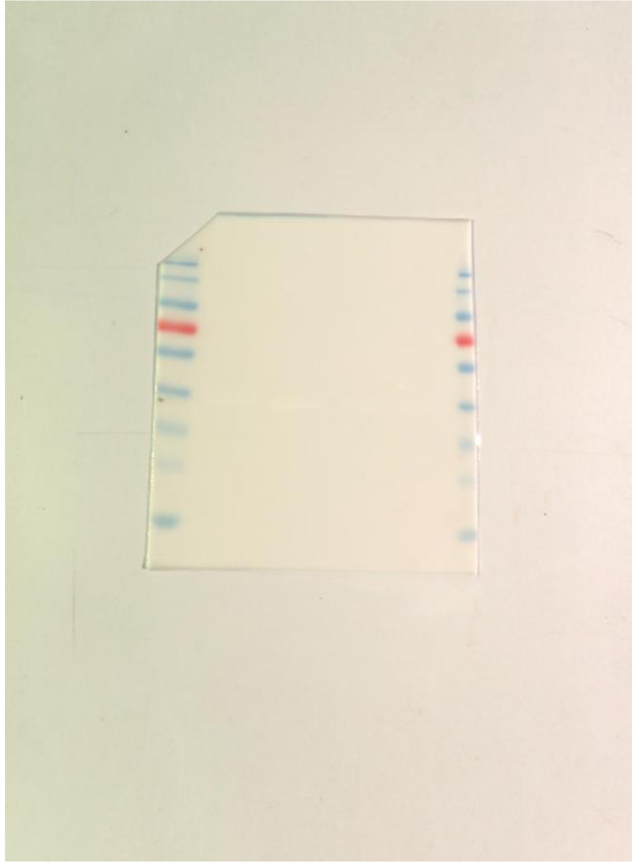

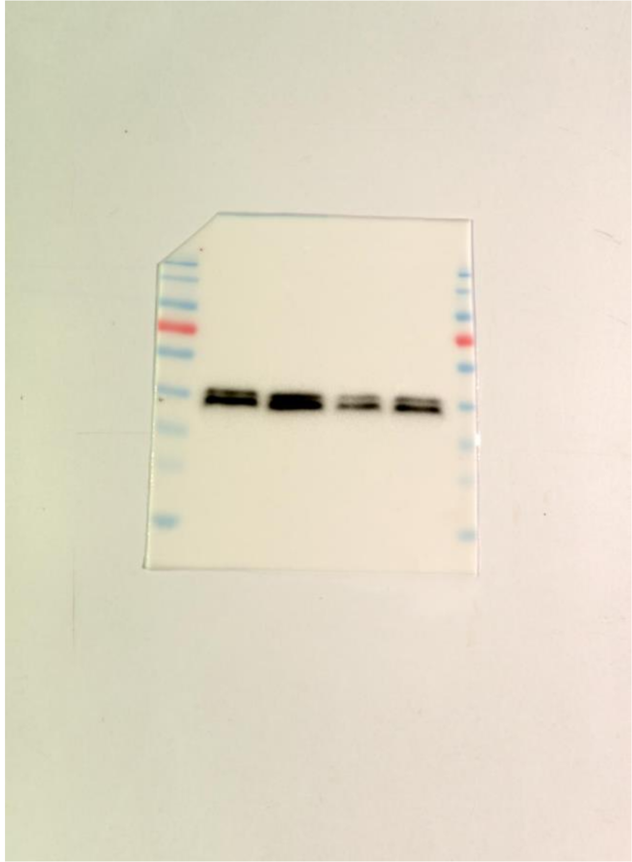

AMC-HN-8

Fig. 5B

GAPDH

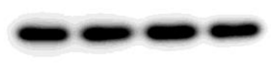

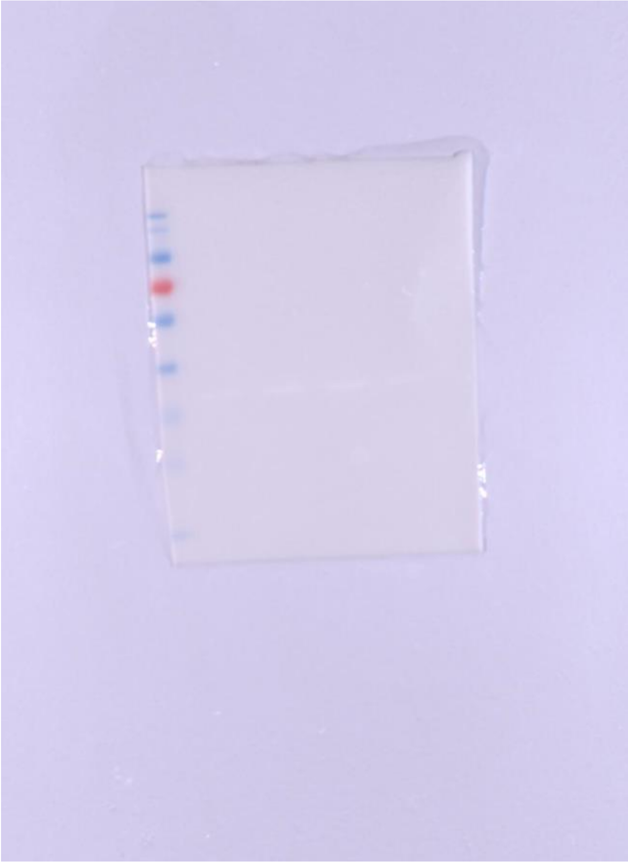

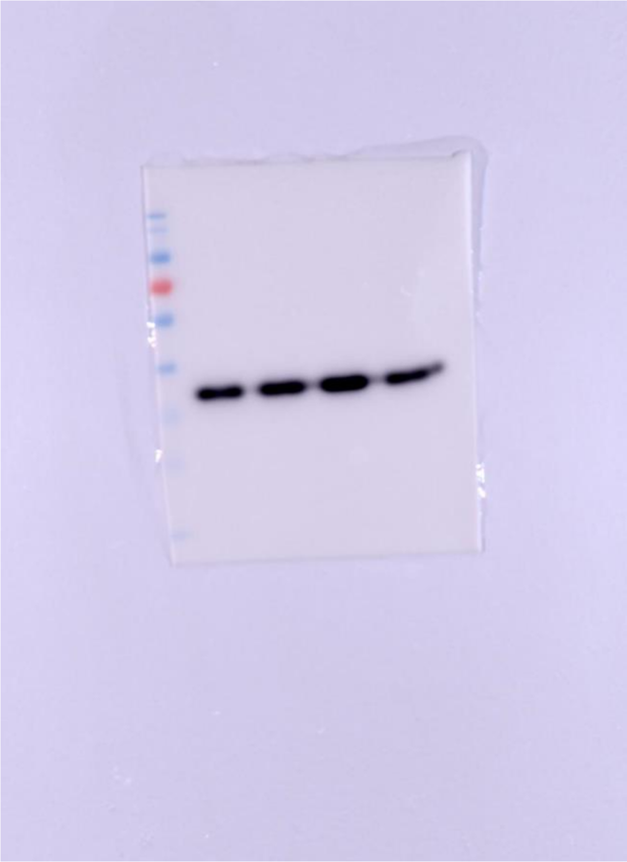

TU212

Fig. 5B

ERK

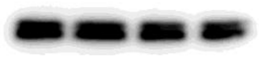

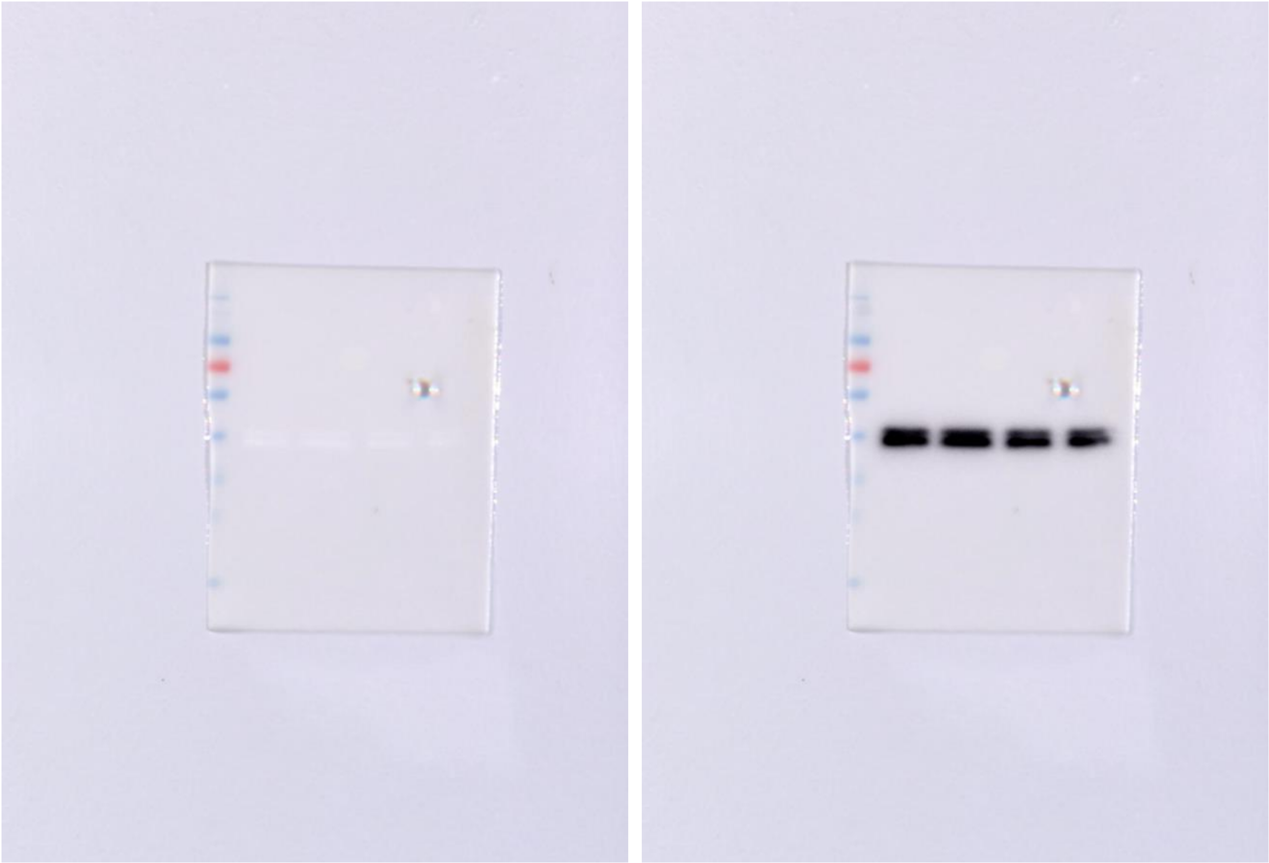

TU212

Fig. 5B

P-ERK

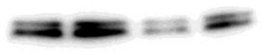

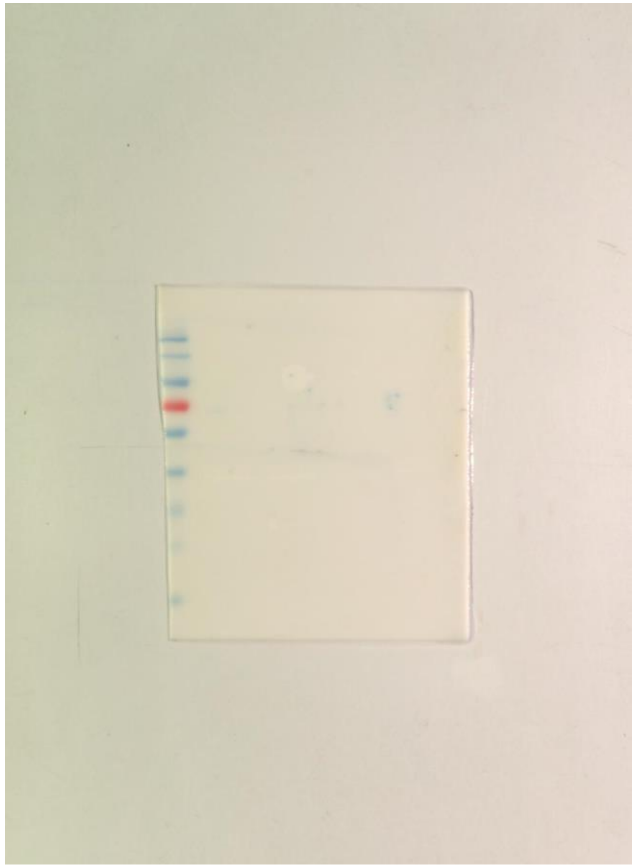

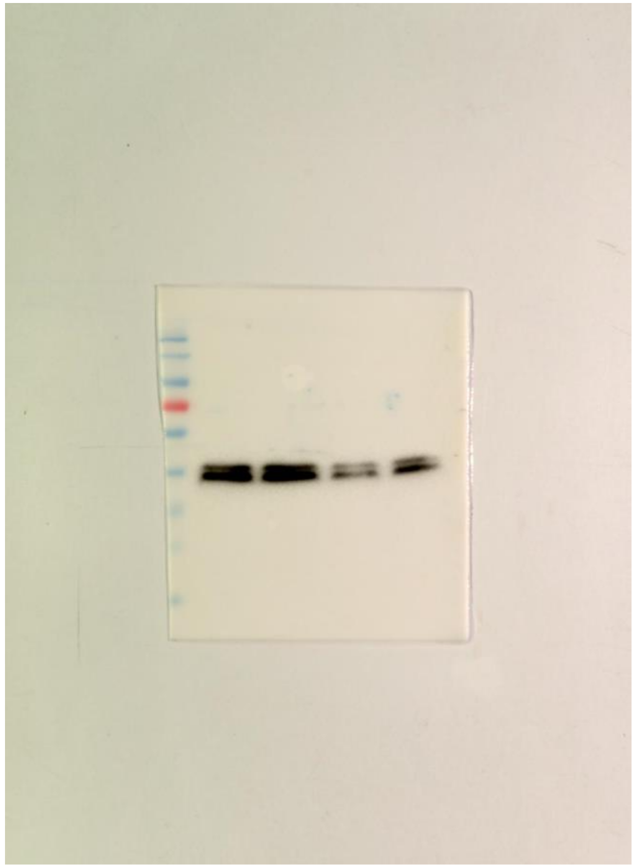

TU212

Fig. 5B

GAPDH

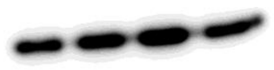

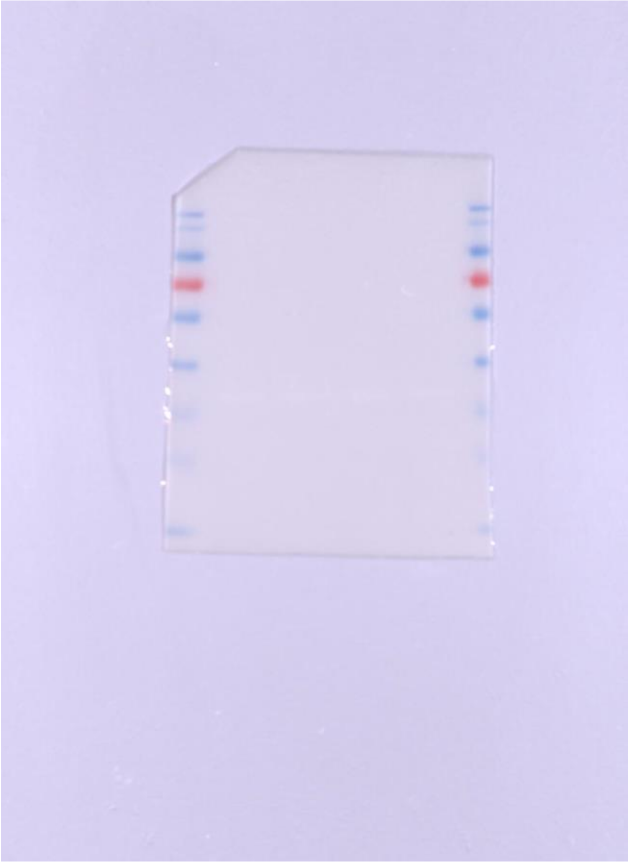

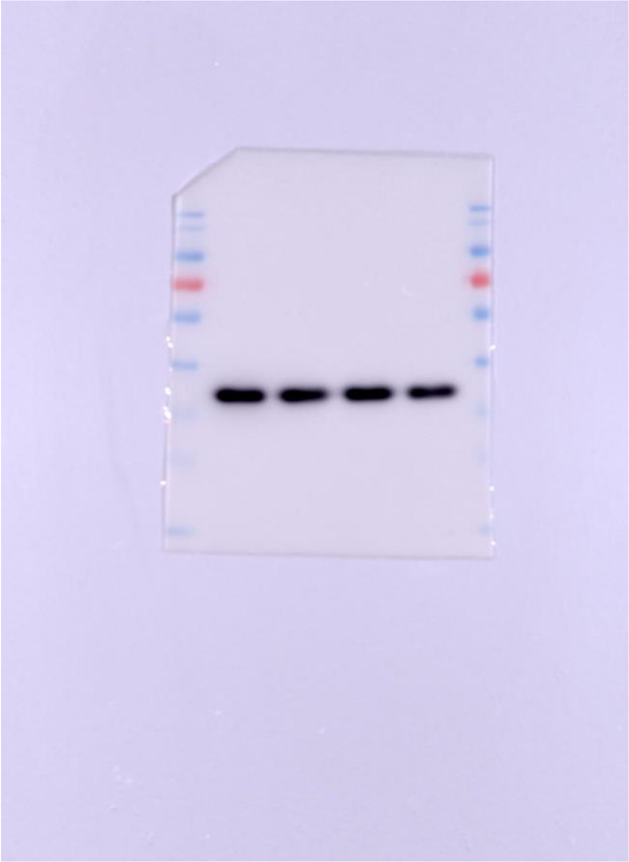

Fig. 5C

AMC-HN-8

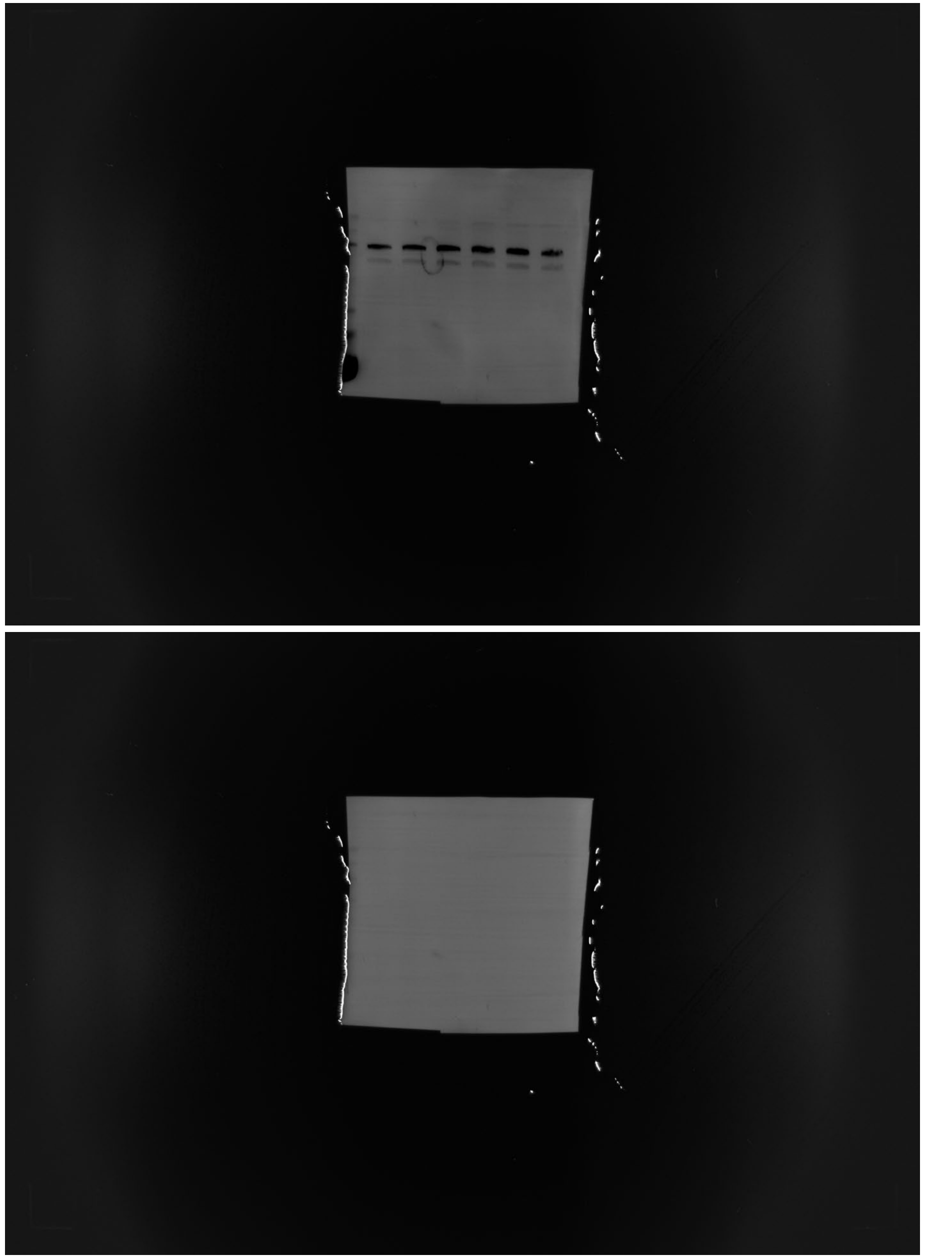

TU212

ERK

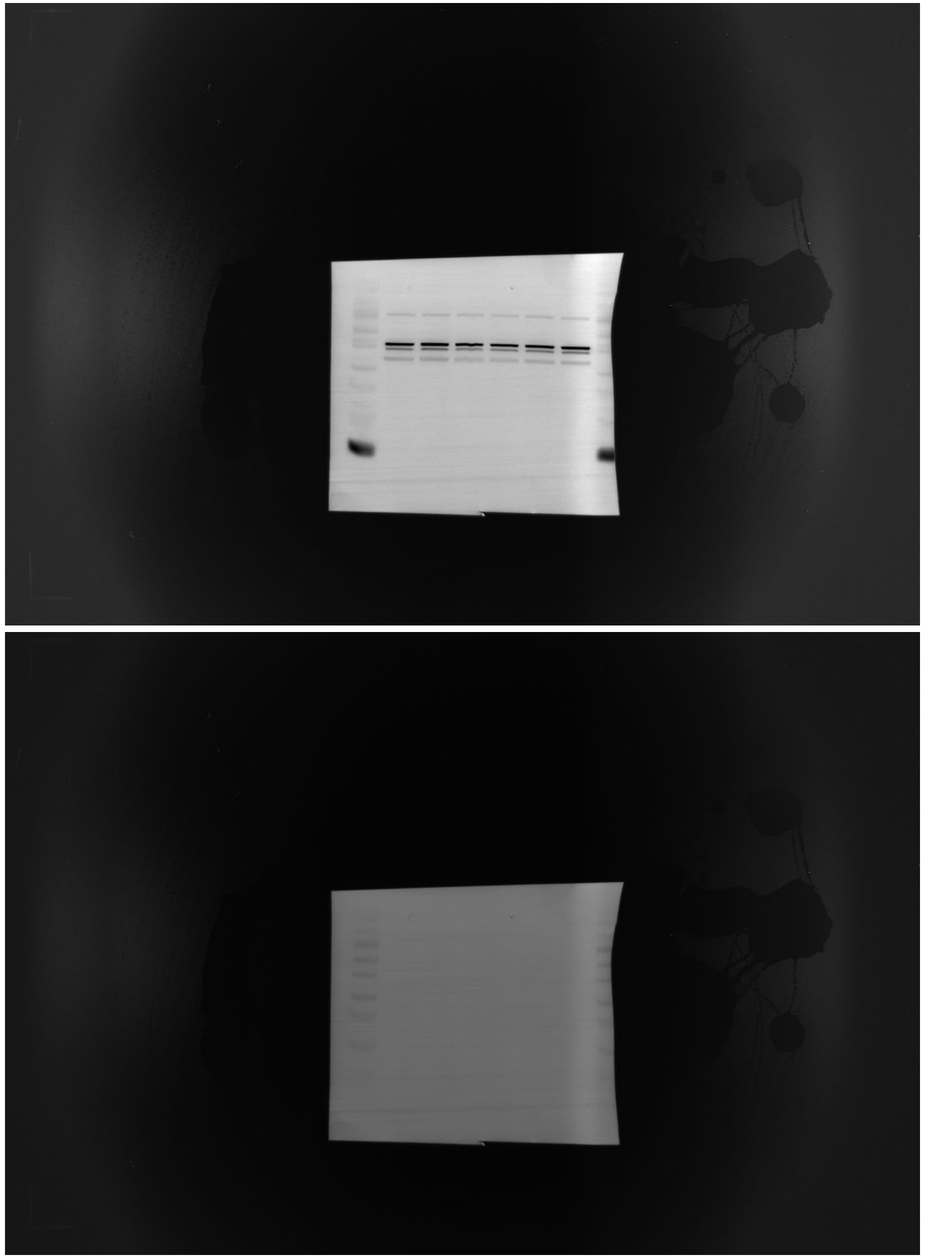

Fig. 5C

AMC-HN-8

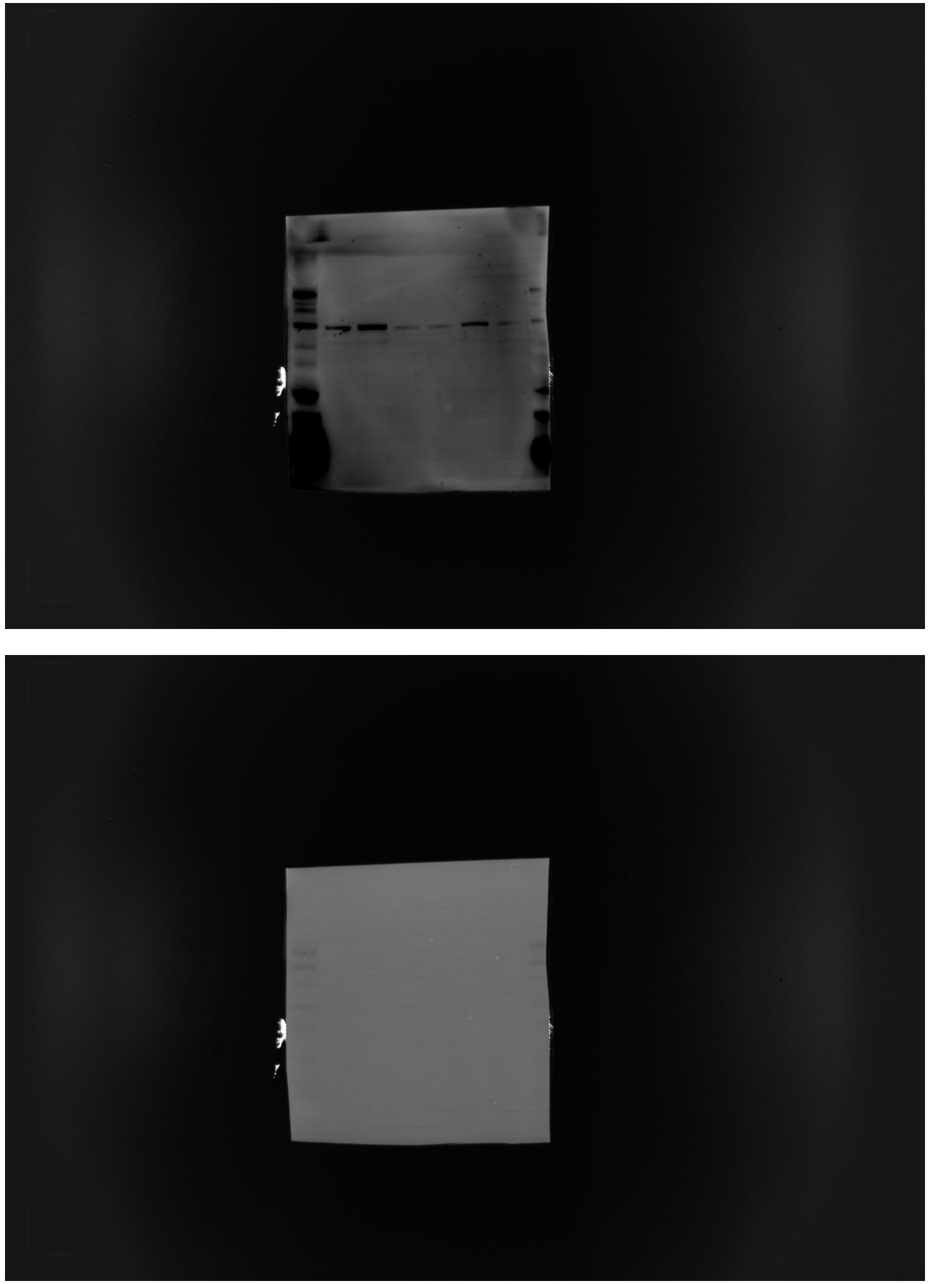

TU212

P-ERK

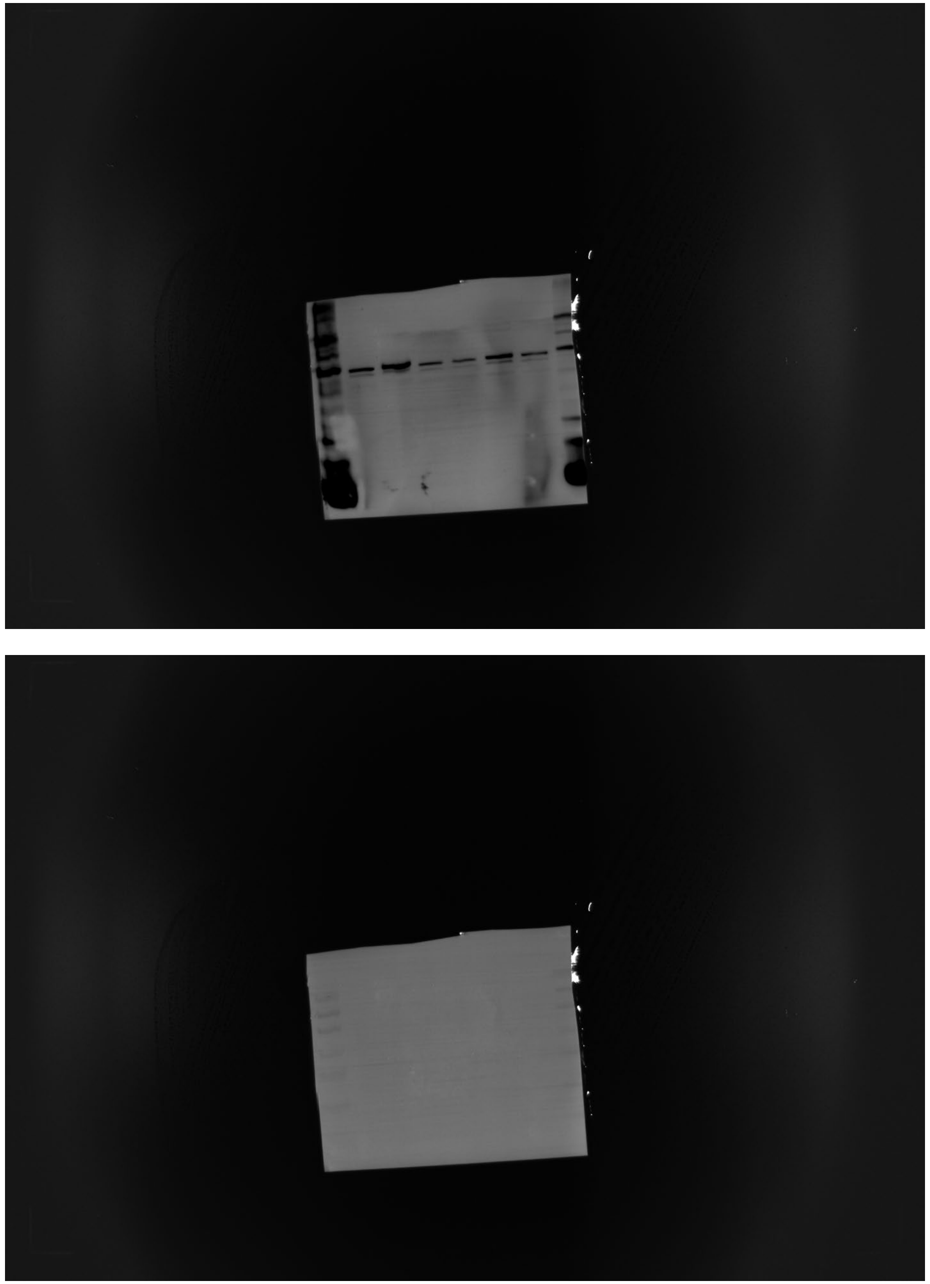

Fig. 5C

AMC-HN-8

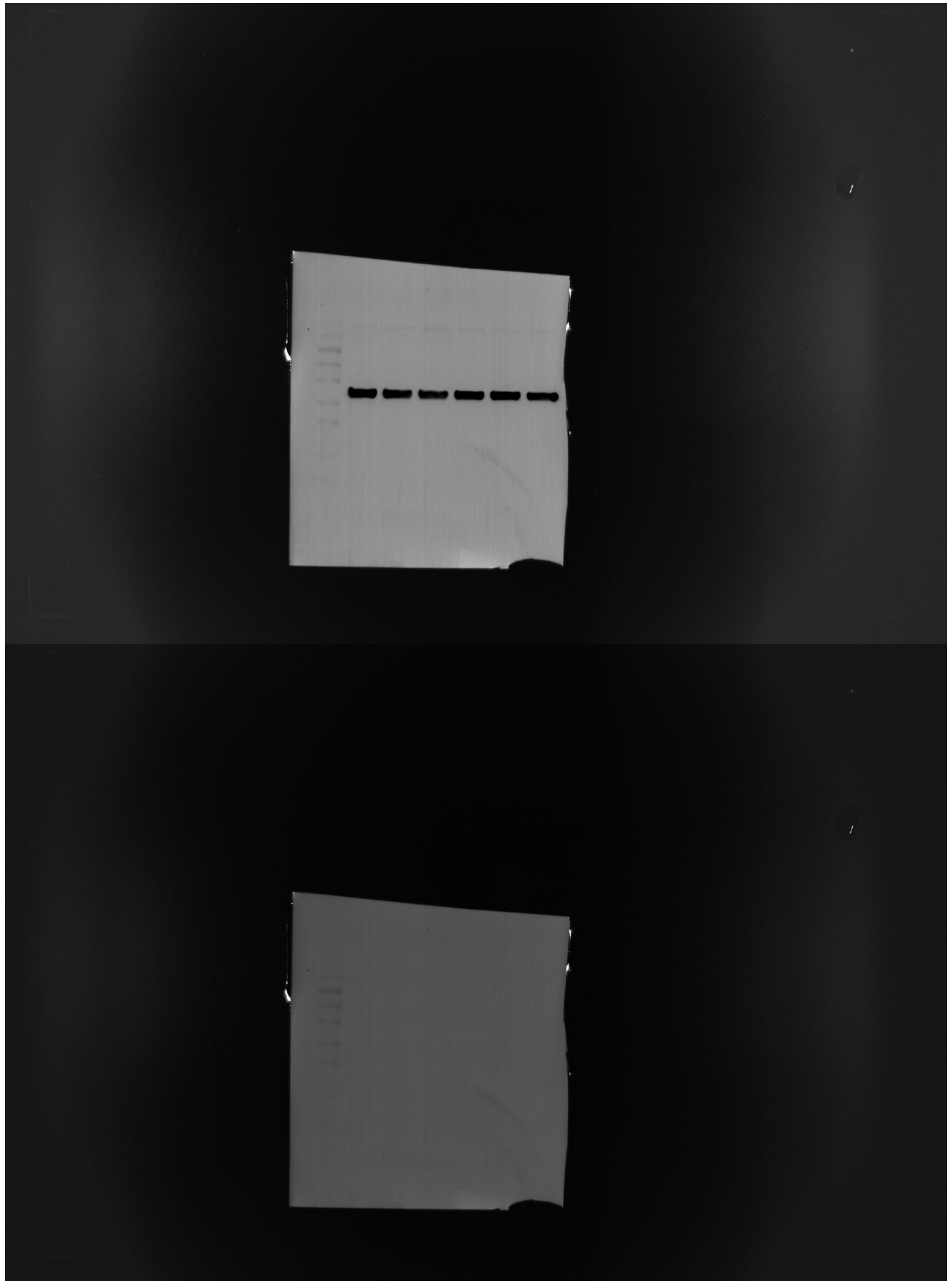

TU212

GAPDH

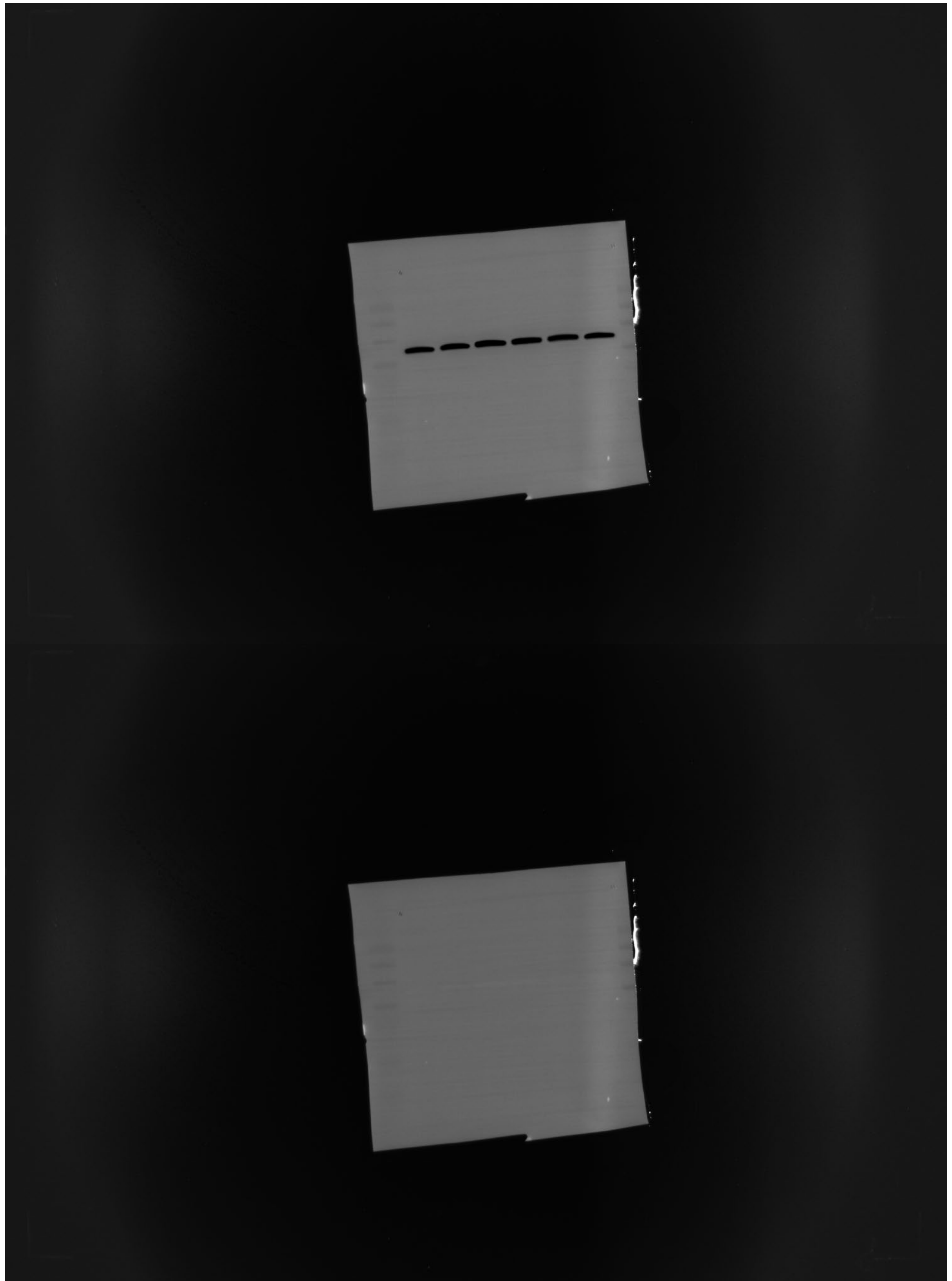

Fig. 5D

AMC-HN-8

TU212

ERK

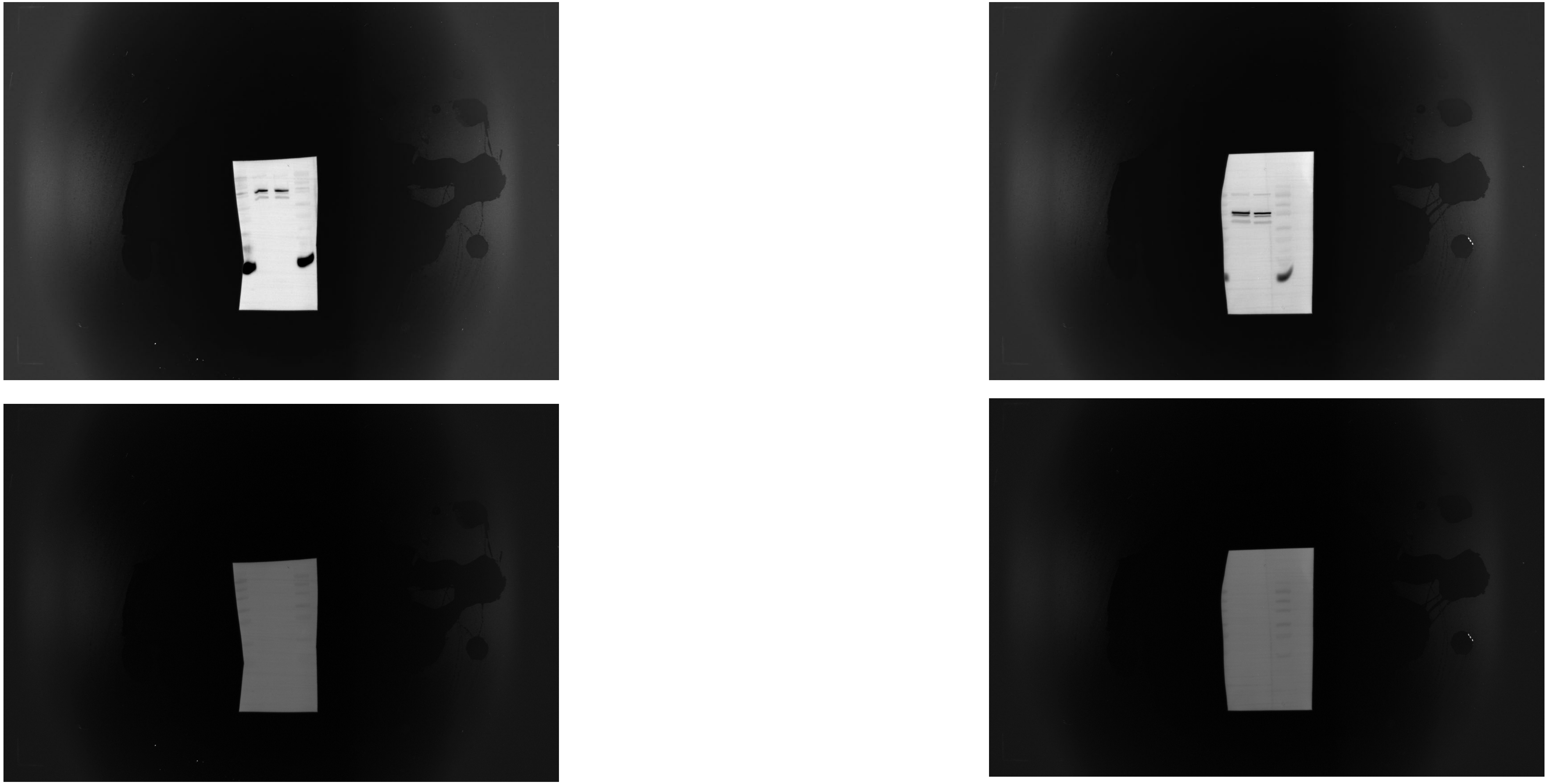

Fig. 5D

AMC-HN-8

TU212

P-ERK

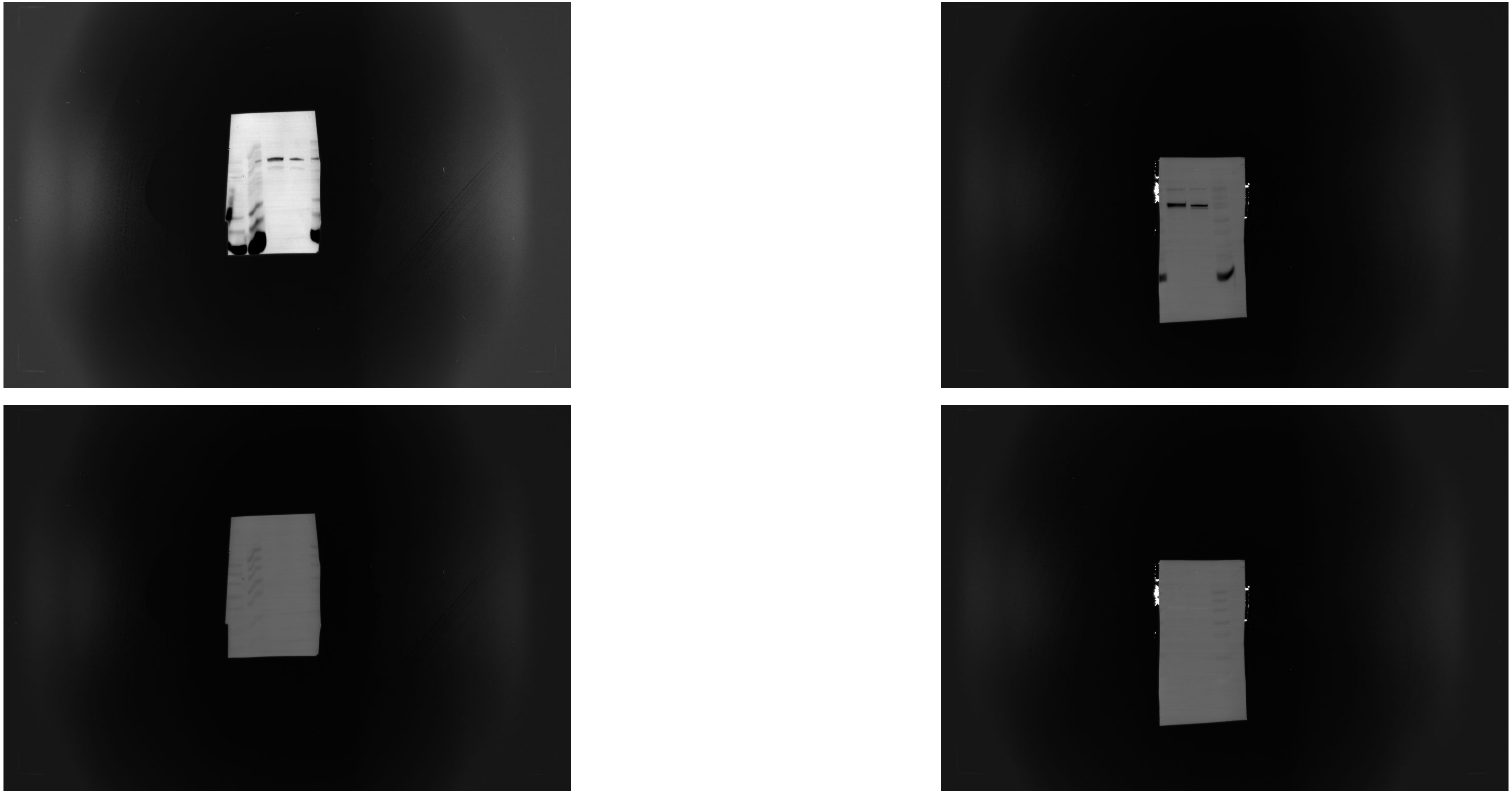

Fig. 5D

AMC-HN-8 **TU212**

GAPDH

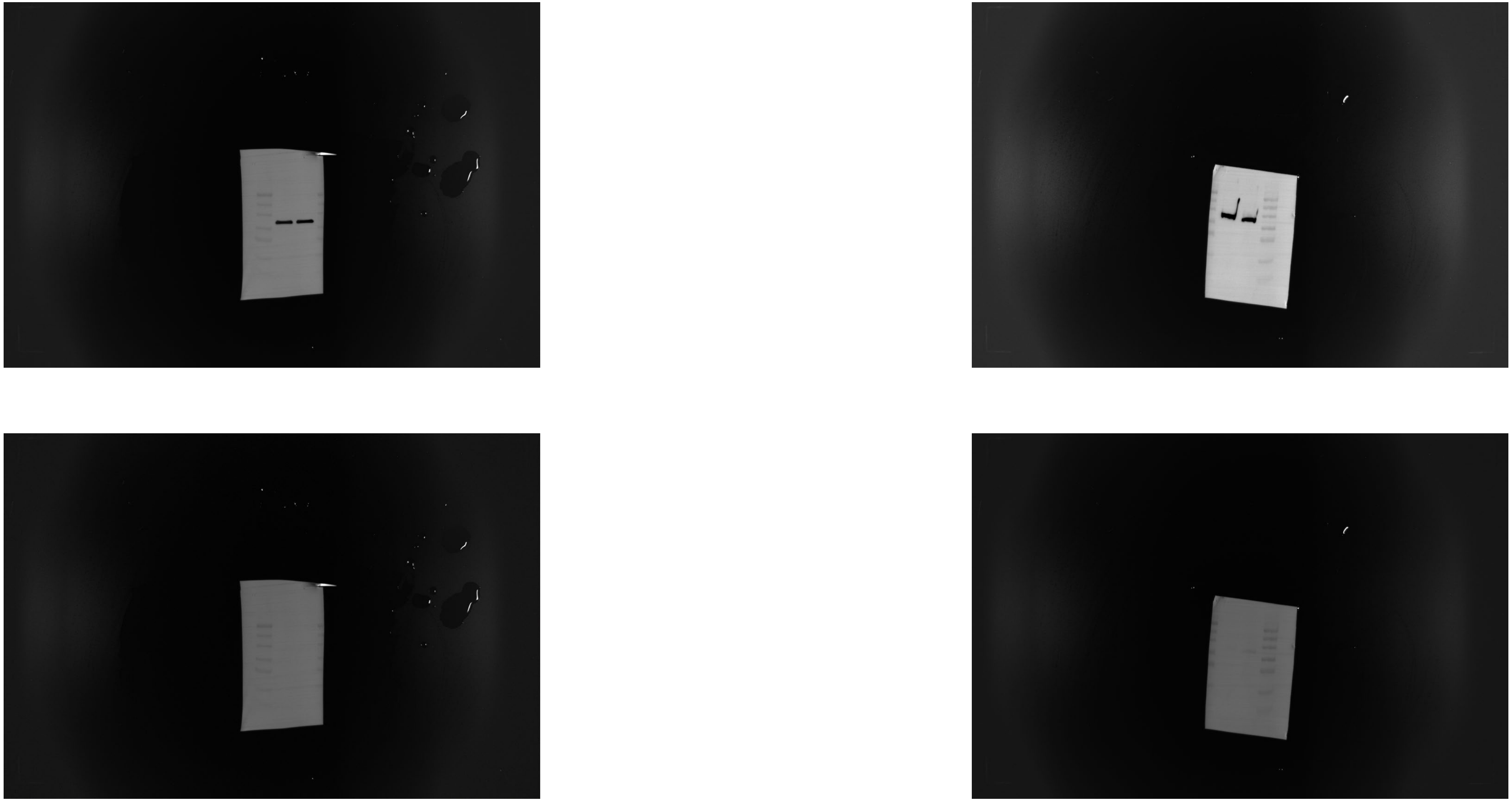

Fig. 5E

AMC-HN-8

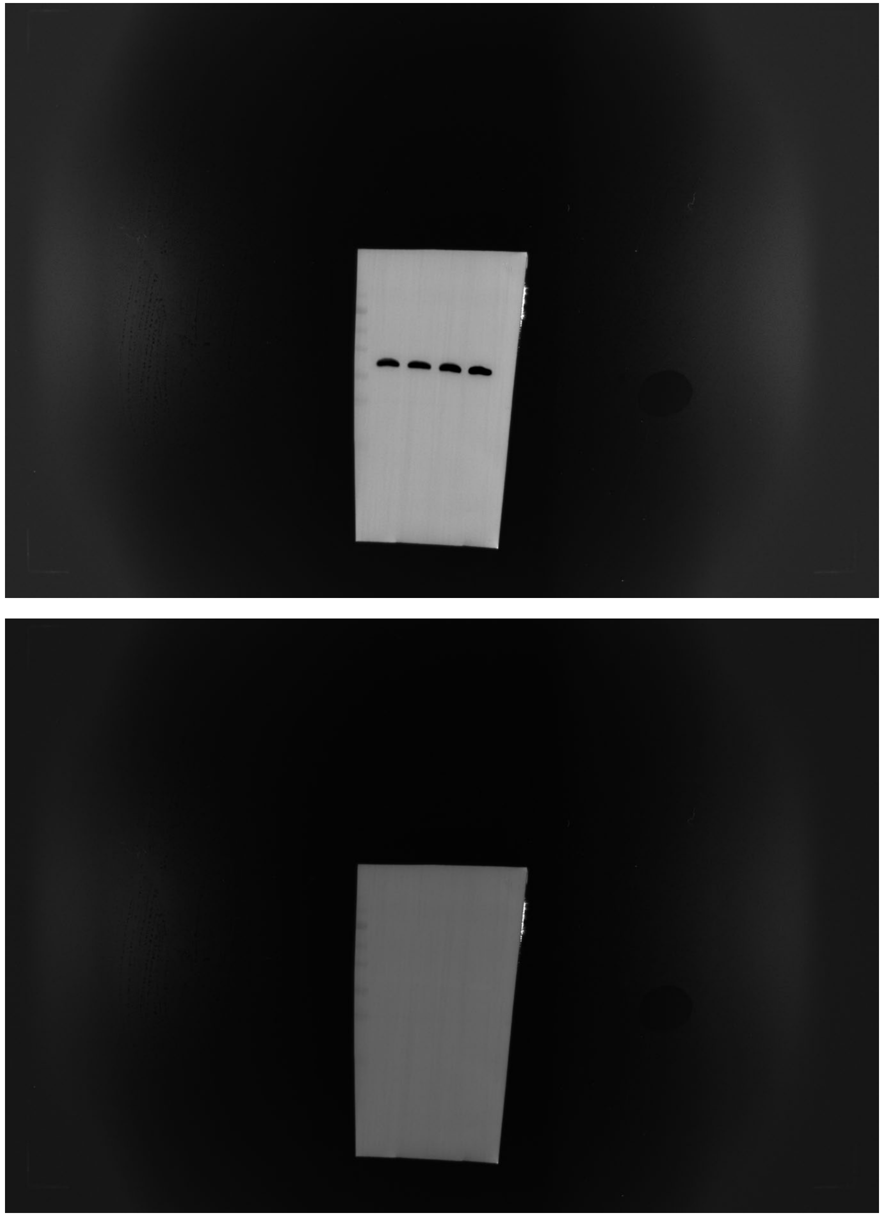

ERK

TU212

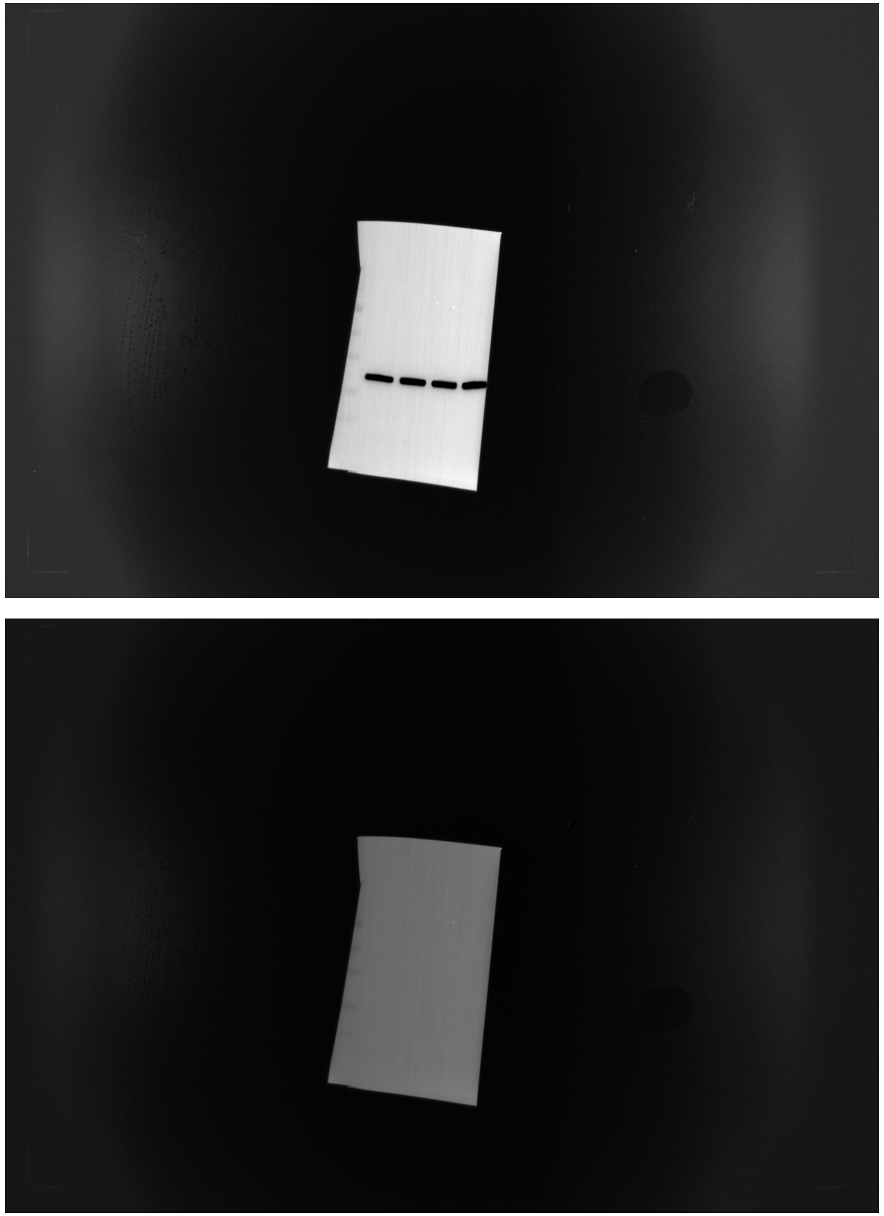

Fig. 5E

AMC-HN-8

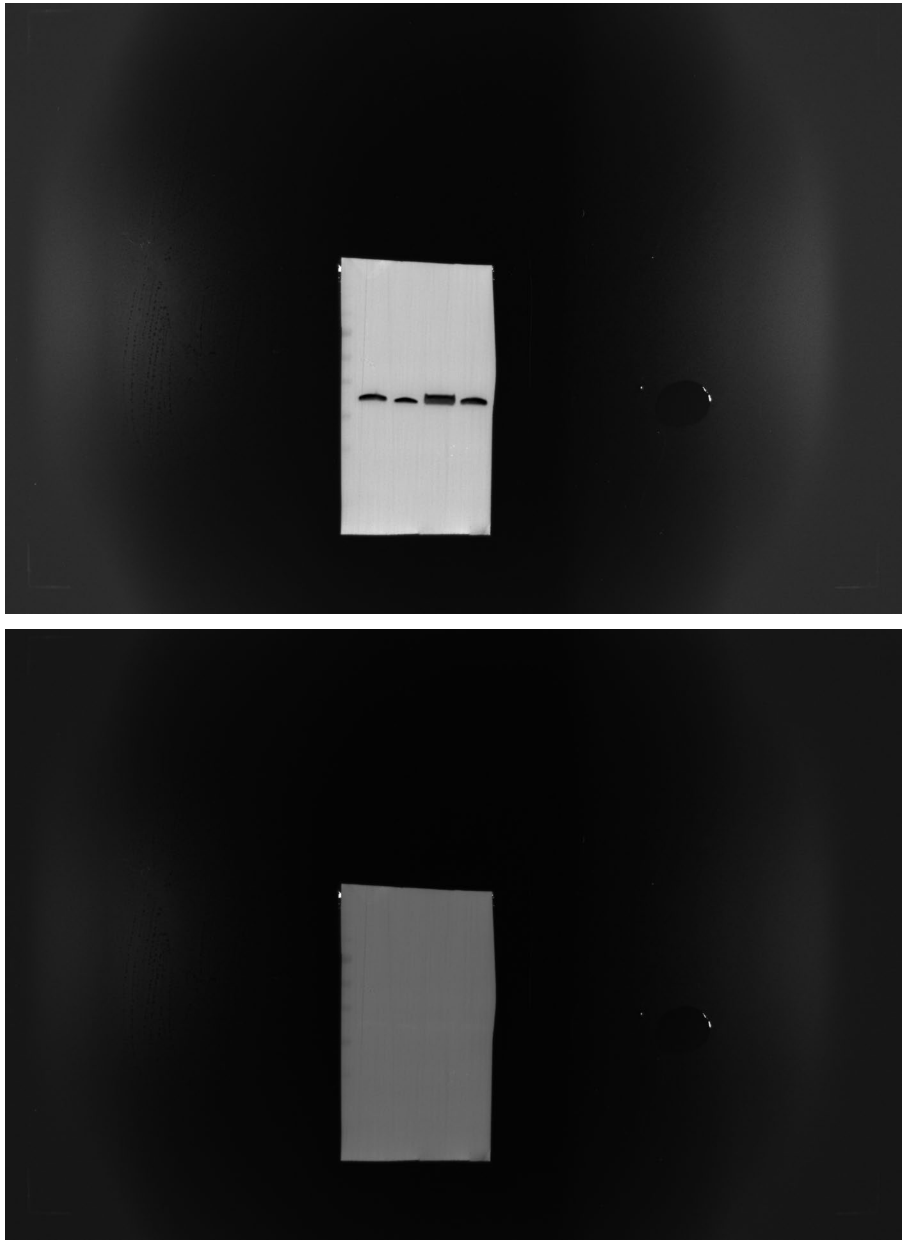

TU212

P-ERK

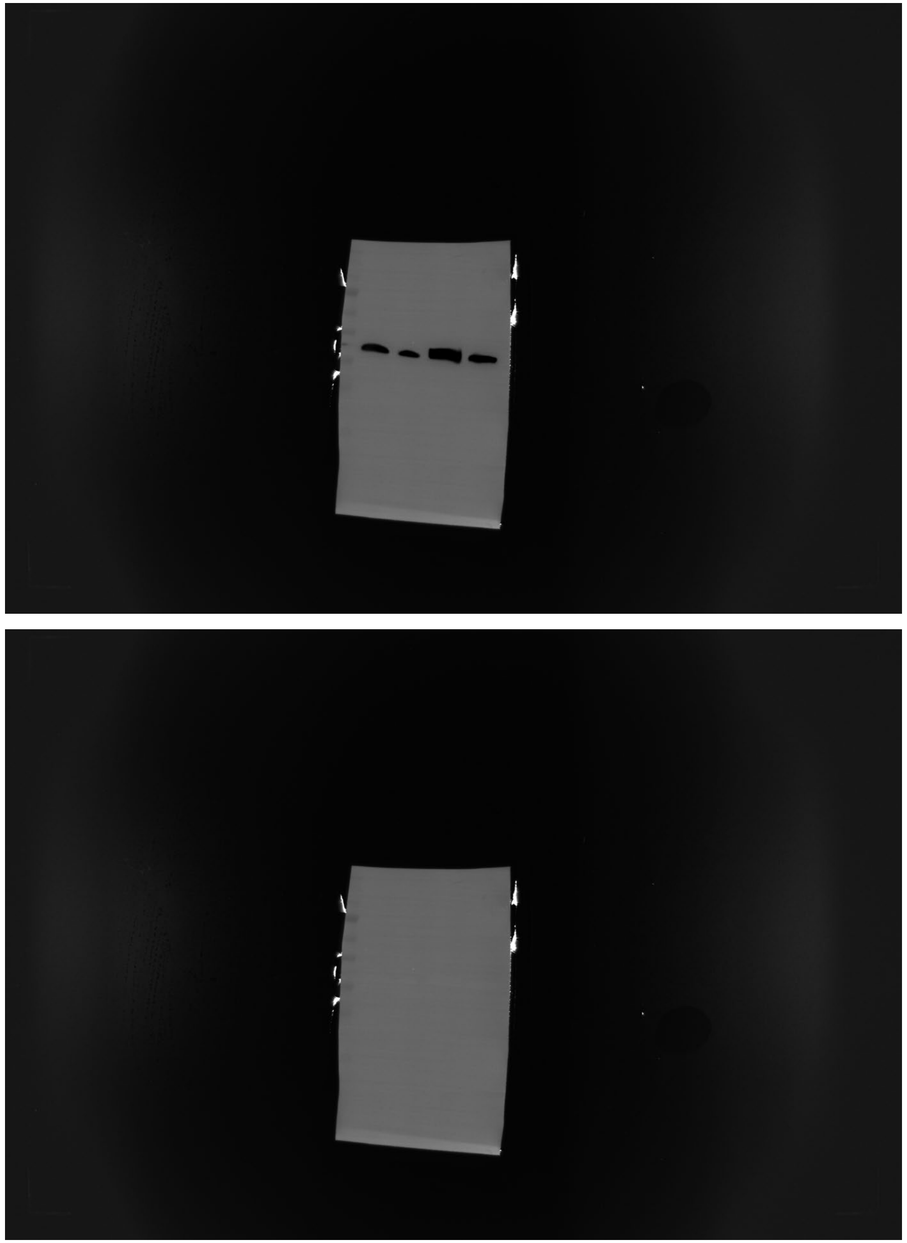

Fig. 5E

AMC-HN-8

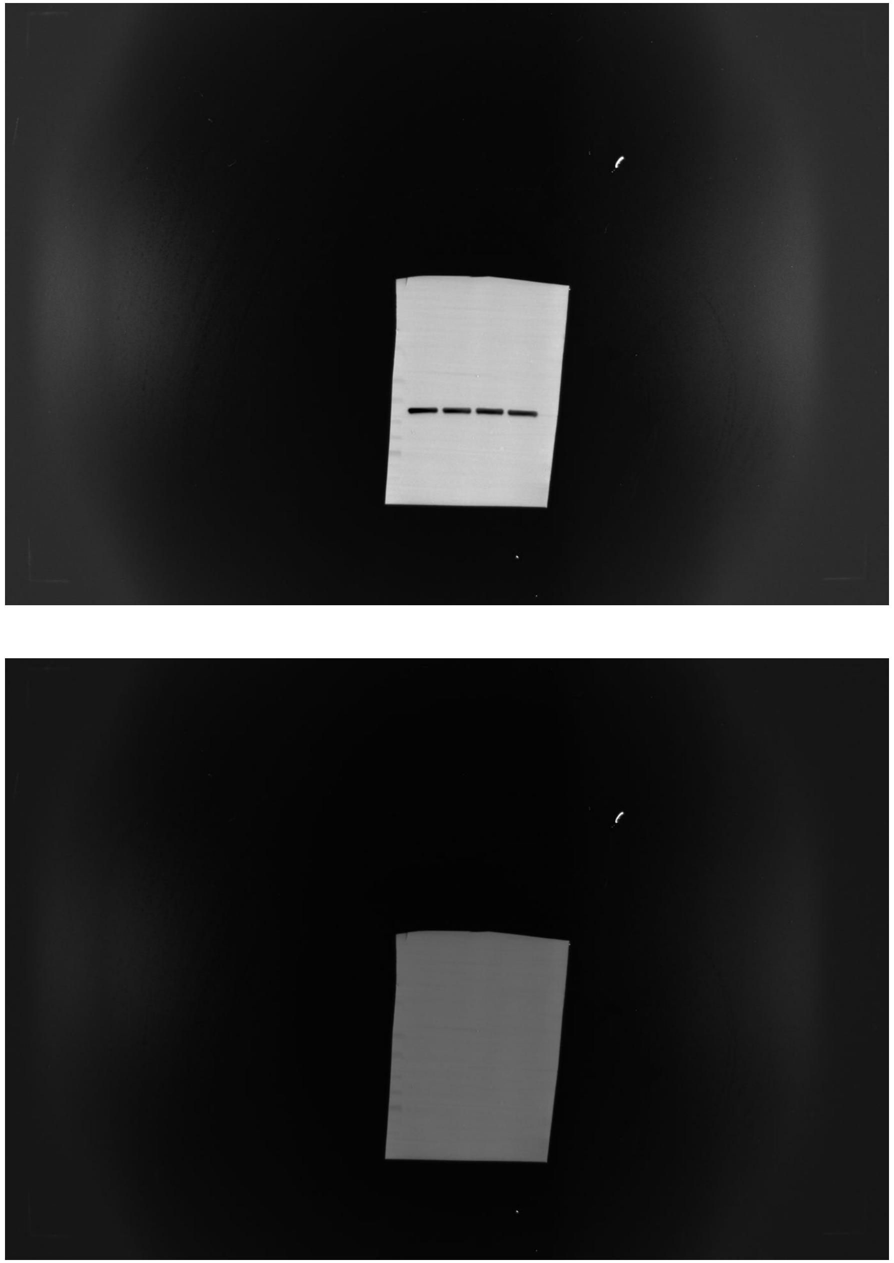

TU212

GAPDH

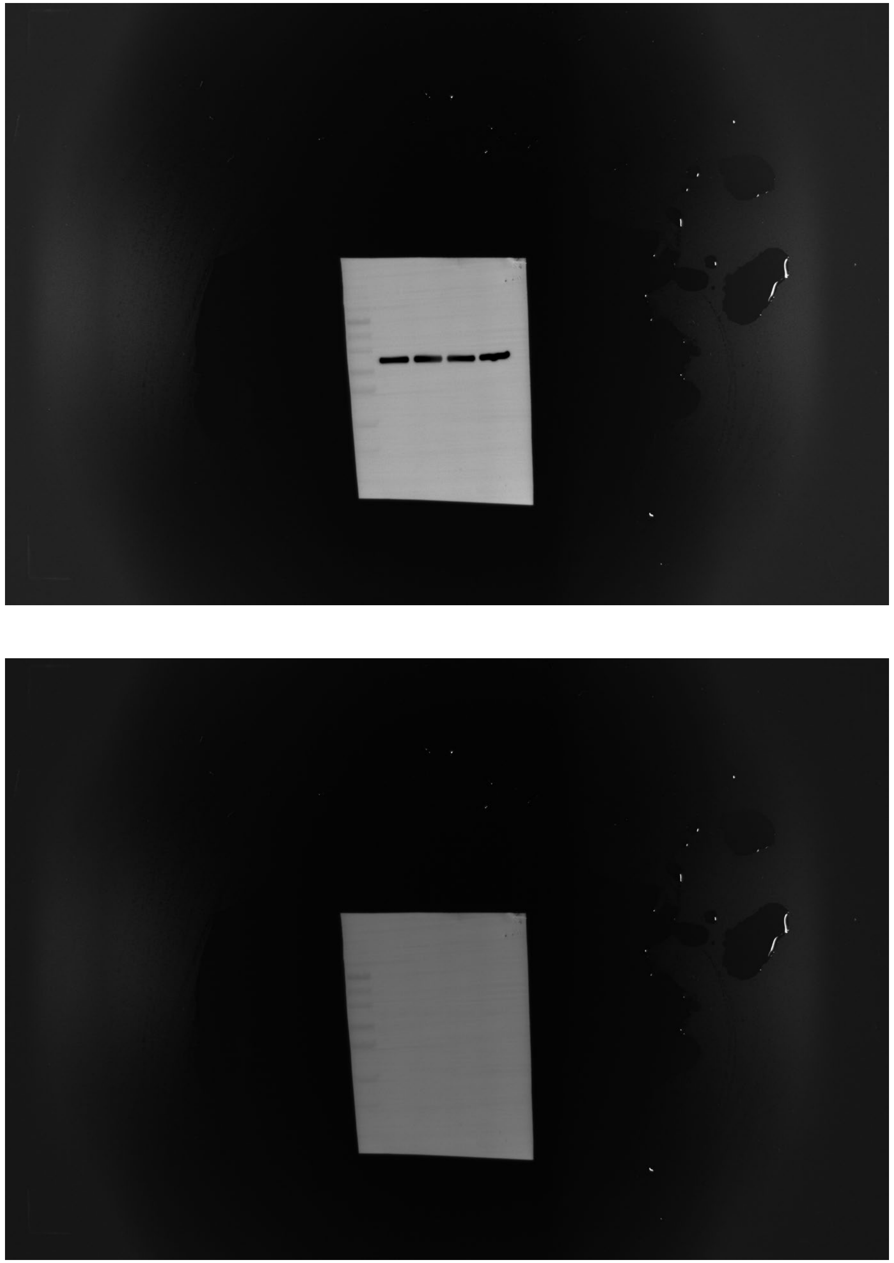

AMC-HN-8

Fig. S2C

RPS6KA1

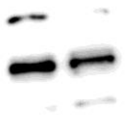

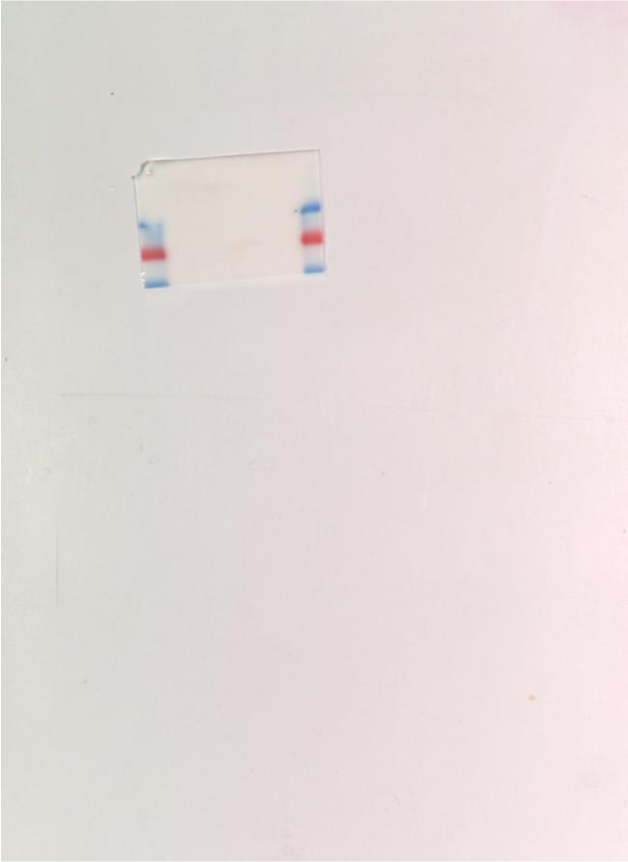

MAPKAP1

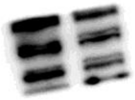

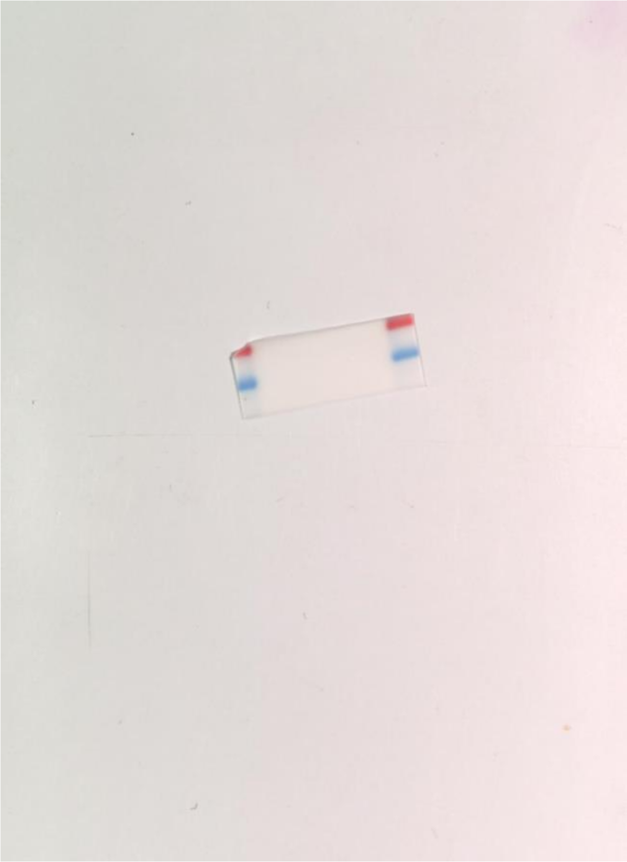

AMC-HN-8

Fig. S2C

EGR1

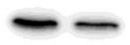

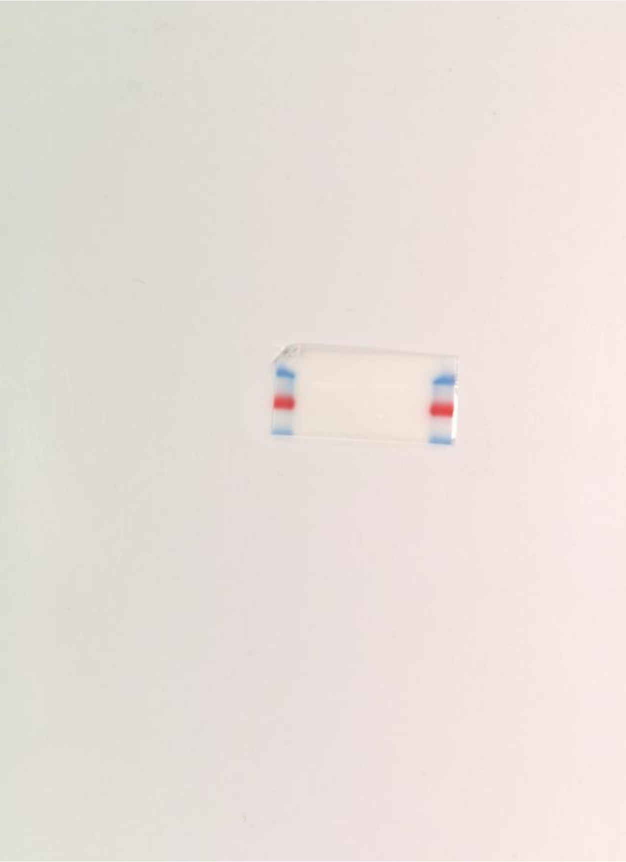

MAP2K2

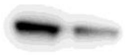

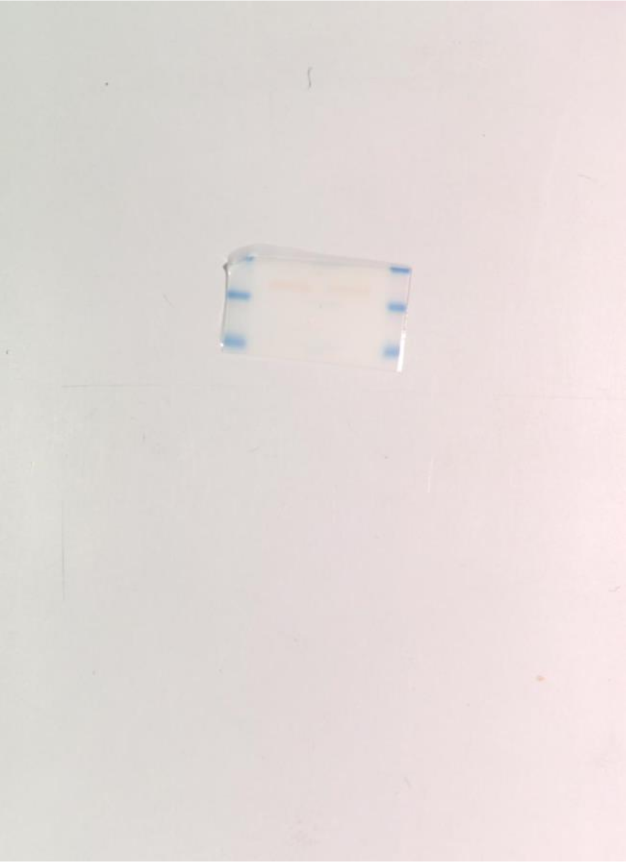

AMC-HN-8

MAPK3

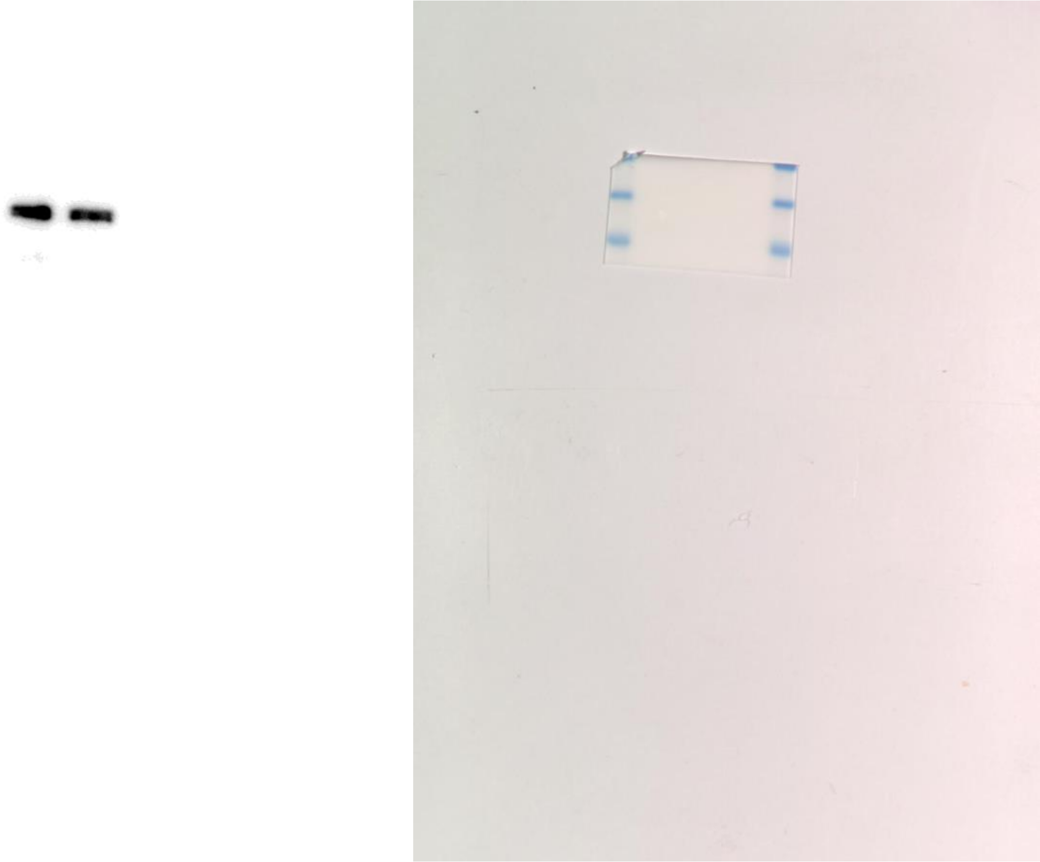

GAPDH

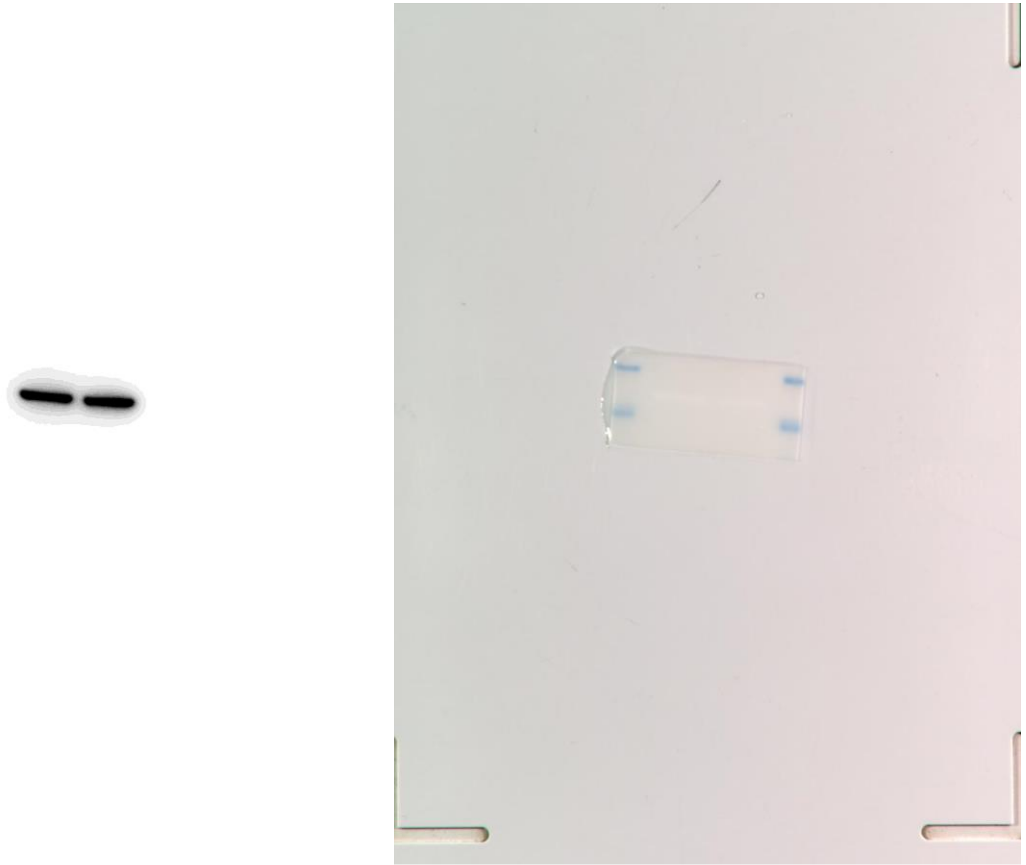

Fig. S3D

MAP2K2

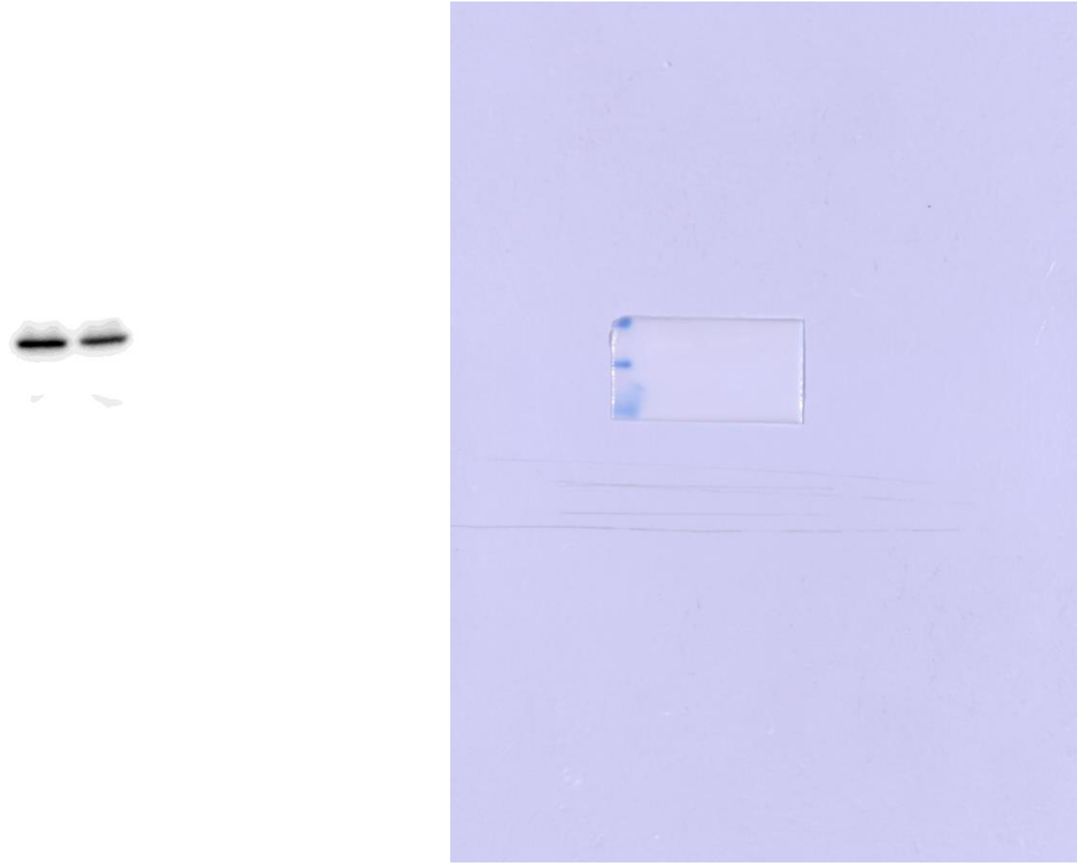

GAPDH

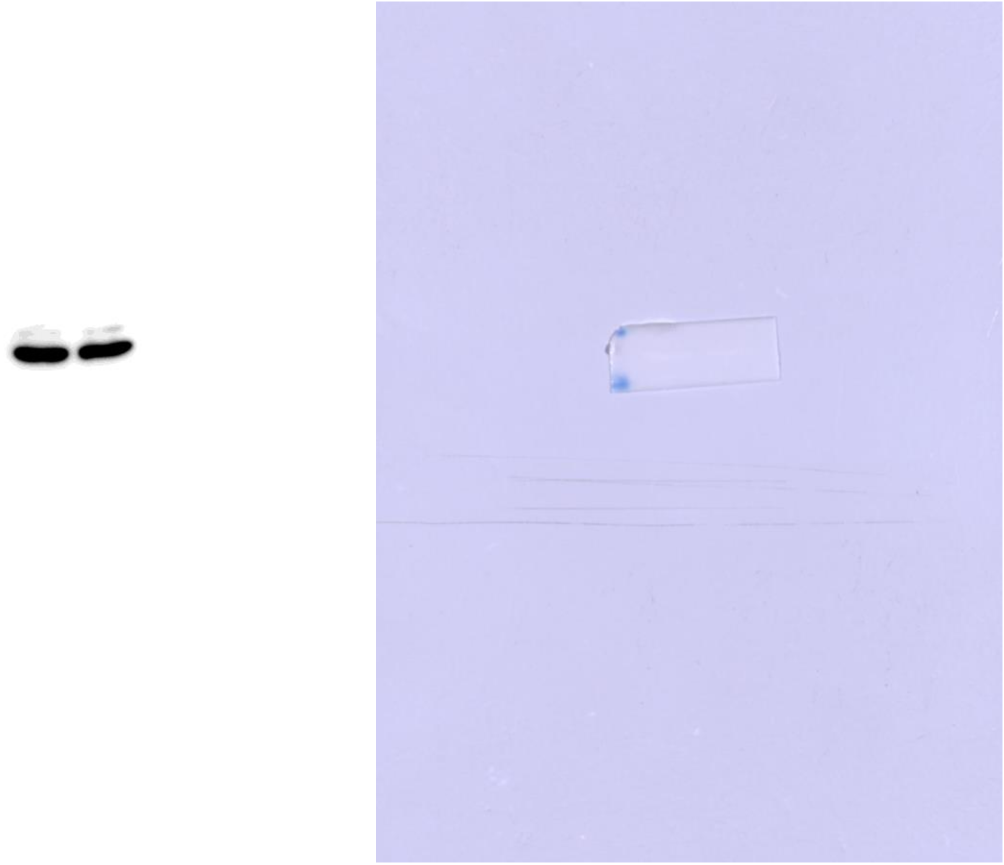

Fig. S3D

EIF3B

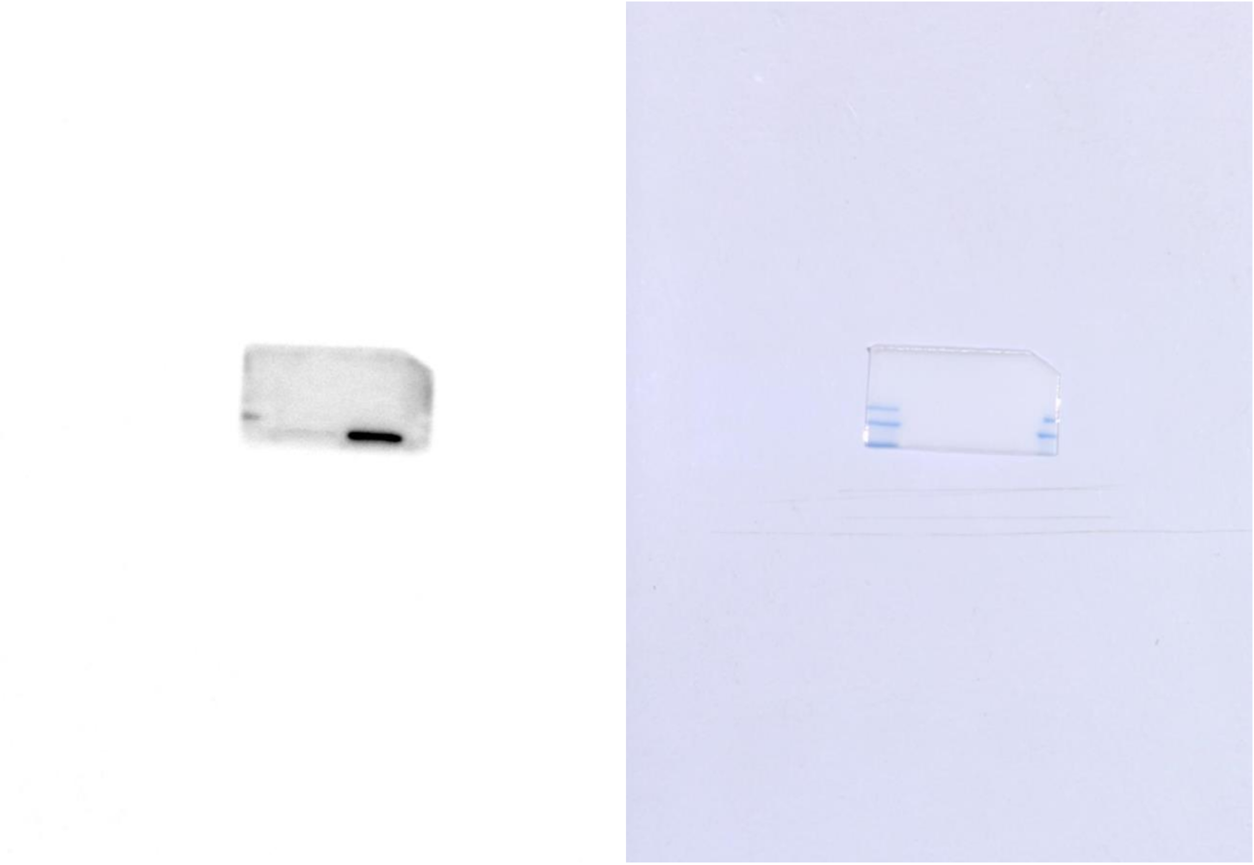

GAPDH

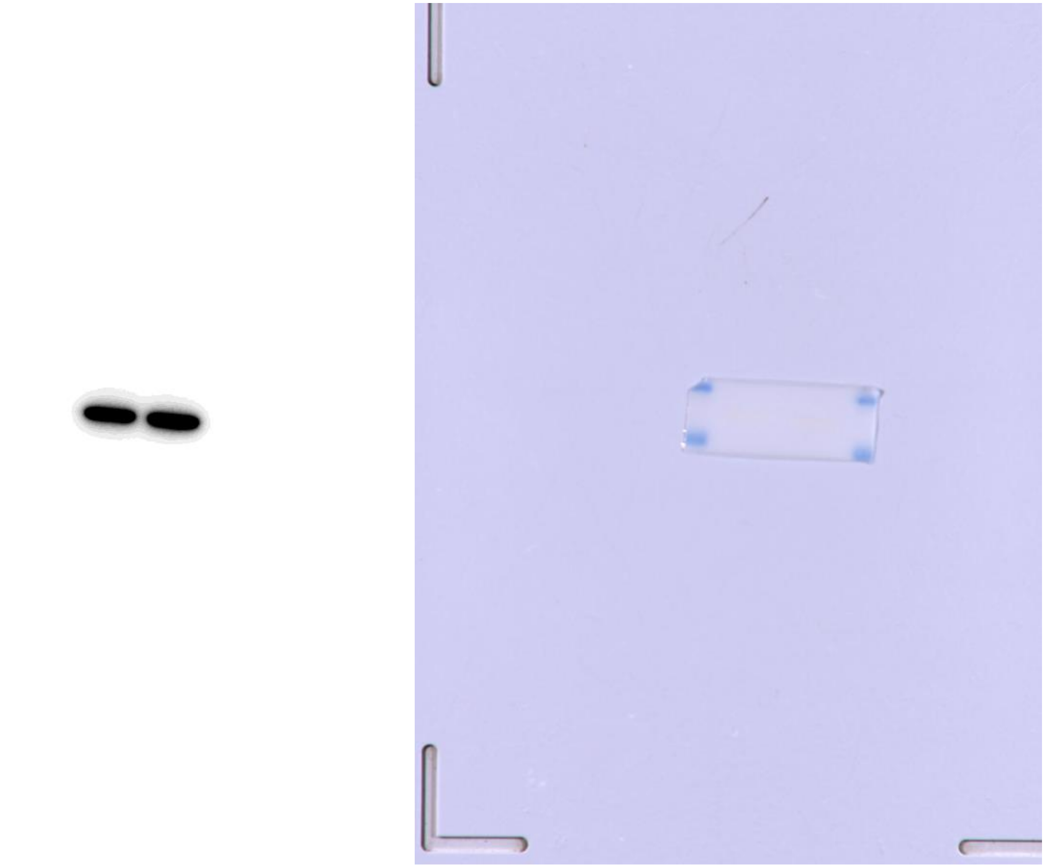

Supplement: Supplementary file 3 — WB images [file 41420_2025_2634_MOESM3_ESM.pdf]
